# A family of small, cyclic peptides buried in preproalbumin since the Eocene epoch

**DOI:** 10.1002/pld3.42

**Published:** 2018-02-28

**Authors:** Mark F. Fisher, Jingjing Zhang, Nicolas L. Taylor, Mark J. Howard, Oliver Berkowitz, Aleksandra W. Debowski, Bahar Behsaz, James Whelan, Pavel A. Pevzner, Joshua S. Mylne

**Affiliations:** ^1^ School of Molecular Sciences The University of Western Australia, Crawley Perth WA Australia; ^2^ ARC Centre of Excellence in Plant Energy Biology The University of Western Australia Crawley Perth WA Australia; ^3^ Centre for Microscopy, Characterisation and Analysis The University of Western Australia Crawley Perth WA Australia; ^4^ Department of Animal, Plant and Soil Sciences School of Life Sciences & ARC Centre of Excellence in Plant Energy Biology AgriBio The Centre for AgriBioscience La Trobe University Bundoora Vic. Australia; ^5^ Marshall Centre for Infectious Disease Research and Training School of Biomedical Sciences The University of Western Australia Crawley Perth WA Australia; ^6^ Department of Computer Science & Engineering University of California La Jolla San Diego CA USA

**Keywords:** asparaginyl endopeptidase, Asteraceae, cyclic peptide, orbitide, seed storage albumin

## Abstract

Orbitides are cyclic ribosomally synthesized and post‐translationally modified peptides from plants; they consist of standard amino acids arranged in an unbroken chain of peptide bonds. These cyclic peptides are stable and range in size and topologies making them potential scaffolds for peptide drugs; some display valuable biological activities. Recently, two orbitides whose sequences were buried in those of seed storage albumin precursors were said to represent the first observable step in the evolution of larger and hydrophilic bicyclic peptides. Here, guided by transcriptome data, we investigated peptide extracts of 40 species specifically for the more hydrophobic orbitides and confirmed 44 peptides by tandem mass spectrometry, as well as obtaining solution structures for four of them by nuclear magnetic resonance. Acquiring transcriptomes from the phylogenetically important Corymbioideae subfamily confirmed the precursor genes for the peptides (called *PawS1‐Like* or *PawL1*) are confined to the Asteroideae, a subfamily of the huge plant family Asteraceae. To be confined to the Asteroideae indicates these peptides arose during the Eocene epoch around 45 Mya. Unlike other orbitides, all PawL‐derived Peptides contain an Asp residue, needed for processing by asparaginyl endopeptidase (AEP). This study has revealed what is likely to be a very large new family of orbitides, uniquely buried alongside albumin and processed by AEP.

## 1 INTRODUCTION

**Figure 1:**
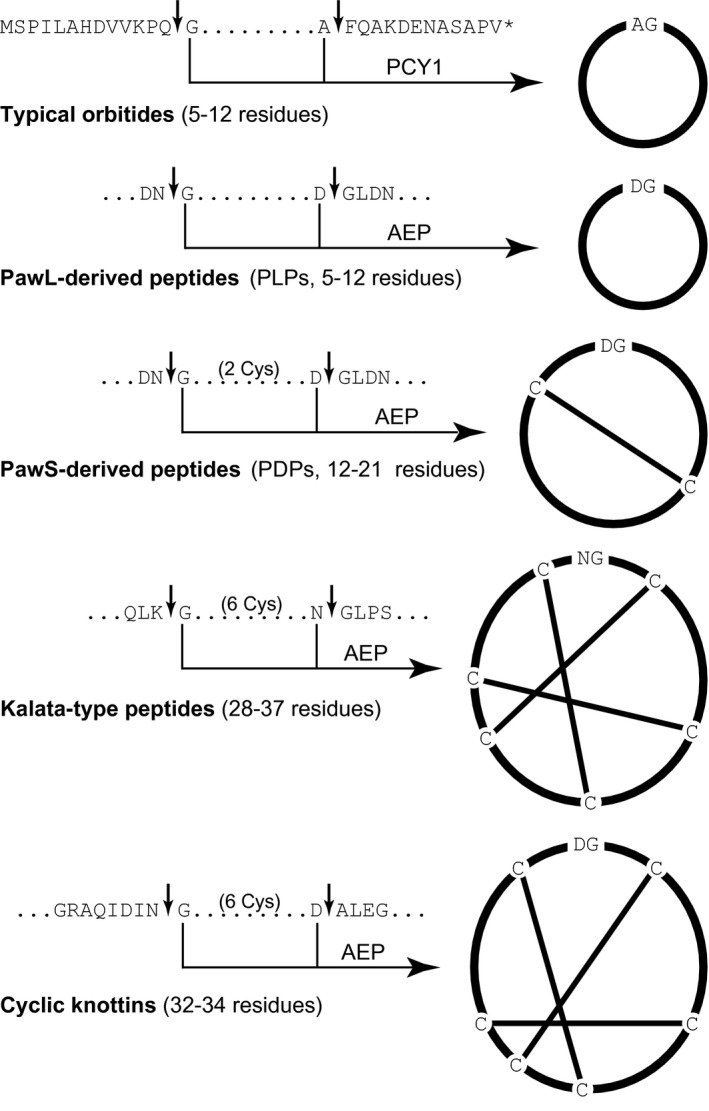
Summary of plant ribosomal cyclic peptides and their biosynthesis. Five classes of ribosomally synthesized cyclic peptides are known to be matured from linear precursor sequences. The points at which the linear sequence is cleaved from the precursor (arrows) and cyclizing enzyme (PCY1 or AEP) are shown. Residues shown are conserved, and dots denote nonconserved sequence. The sequence for the typical orbitides is based on the segetalins of *Vaccaria hispanica*

Cyclic peptides form a class of biomolecules that are found across all domains of life (Wang, Kaas, Chiche, & Craik, 2008). Their structural stability and protease resistance have generated considerable interest in using them as scaffolds for the synthesis of novel bioactive molecules (White & Craik, 2016). Most cyclic peptides have been discovered in plants from pharmacognosy and bioactivity approaches (Figure 1); this includes Cys‐rich cyclic peptides such as kalata B1 and cyclic squash knottins, as well as smaller peptides that contain only a single disulfide bond, such as SFTI‐1 (Hernandez et al., 2000; Luckett et al., 1999; Saether et al., 1995). The first plant cyclic peptides discovered, however, lacked disulfide bonds and were referred to initially as Caryophyllaceae‐type cyclic peptides, later termed orbitides.

Orbitides are homodetic cyclic peptides (i.e., head‐to‐tail cyclic peptides containing only standard amino acids) lacking disulfide bonds, which contain 5–12 amino acid residues (Arnison et al., 2013). Despite being known for decades (Tan & Zhou, 2006), their biosynthetic origins remained elusive and were presumed to be nonribosomal until the first precursor genes were cloned by Condie et al. (2011) from *Vaccaria hispanica* (*Saponaria vaccaria)*. The authors showed how these genes produced short precursor proteins that were processed into orbitides called segetalins by the action of two proteases. The enzyme that cyclizes segetalins is a serine protease called PCY1 (Barber et al., 2013). The crystal structure of PCY1 was recently solved and shown to ligate amino acids by shielding the transamidation site of the enzyme‐substrate intermediate from solvent, inhibiting hydrolysis (Chekan, Estrada, Covello, & Nair, 2017).

Recent work that traced the evolutionary history of a bicyclic protease inhibitor, the prototypic two‐Cys cyclic peptide SFTI‐1, found that the evolutionary step preceding SFTI‐1 and other bicyclic peptides was an orbitide (Jayasena et al., 2017). SFTI‐1 and its related peptides are characterized by being buried within a precursor protein that also encodes a napin‐type seed storage albumin. The first such gene discovered was from the common sunflower *Helianthus annuus* and named *Preproalbumin with SFTI‐1* or *PawS1*. *PawS1* consists of an endoplasmic reticulum signal, a spacer region containing the sequence for SFTI‐1, and then, after a short tail sequence, the remaining two‐thirds of the protein is dedicated to the two subunits of the mature seed storage albumin. Alongside removal of the endoplasmic reticulum sequence and folding of the protein, the buried peptide and albumin are separated by proteolytic processing by the enzyme asparaginyl endopeptidase (AEP). AEP is a Cys protease that, in plants, appears to have been recruited independently multiple times to perform the macrocyclization reaction responsible for different classes of plant cyclic peptides (Mylne et al., 2012). The structure of a plant AEP has been solved (Yang et al., 2017), and the strong conservation of residues trailing cyclic peptide domains (in particular a P2′ Leu) suggests similar interactions between it and the AEP acyl intermediate might help favor macrocyclization reactions as seen previously with PCY1 (Barber et al., 2013; Chekan et al., 2017).

To retrodict the evolutionary history of SFTI‐1, Jayasena et al. (2017) assembled transcriptomes for 110 members of the Asteraceae or sunflower family and searched them for *PawS1* genes and their relatives. Genes whose buried peptide region was shorter and lacked Cys residues were called *PawS1‐Like* genes (*PawL1*). *PawL1* genes are more widespread than *PawS1* genes, being found throughout the Asteroideae, which diverged from a common ancestor 45 Mya. Strong conservation of the region that, in *PawS1*, makes SFTI‐1 or similar PawS‐Derived Peptides (PDPs) suggested a smaller peptide lacking Cys residues was being made from the equivalent region of PawL1 proteins. In general, PDPs are hydrophilic; examining the aqueous phase of peptide extractions for PDPs did not detect masses matching potential PawL‐derived Peptides (Elliott et al., 2014). However, the first two PawL‐derived Peptides (PLPs) were discovered when analyzing peptide extractions made with more hydrophobic solvents: PLP‐1 from *Zinnia haageana* (cyclo‐AIIPGLID) and PLP‐2 from *Senecio pinnatifolius* var. *maritimus* (cyclo‐DLFVPPID) (Jayasena et al., 2017). The conservation seen within the equivalent regions encoded by *PawL1* genes implied a larger family of cyclic peptides awaited discovery.

Here, we re‐extracted peptides from many of the species studied by Jayasena et al. (2017) and used liquid chromatography–coupled tandem mass spectroscopy (LC‐MS/MS) to confirm the amino acid sequences for 44 PLPs. Although MS/MS cannot distinguish the isobaric residues Ile and Leu, we resolved these using the matching transcriptome data as each PLP had a matching precursor transcript. We found no evidence for *PawL1* genes in newly acquired transcriptomes for members of the subfamily Corymbioideae, confirming that *PawL1* genes and their encoded PLPs are restricted to the Asteroideae subfamily, and so evolved during the Eocene epoch approximately 45 million years ago. Using nuclear magnetic resonance (NMR) spectroscopy, we solved the first tertiary structures for orbitides. The five structures (two are *cis* and *trans* conformers of one PLP) showed these peptides adopt a constrained and flat conformation. Based on their abundance within some species, the hypervariability of their sequences and the rapid speciation (and consequently large size) of the Asteraceae, this orbitide family buried in preproalbumin has the potential to encompass thousands of different family members.

## 2 RESULTS

### 2.1 Transcript‐based discovery and sequencing of 44 PLPs

**Figure 2:**
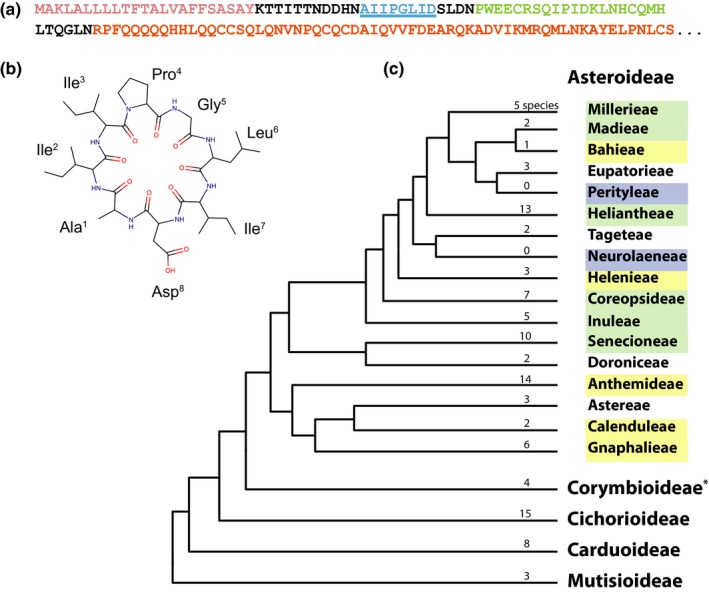
The *PawL1* gene type is confined to the Asteroideae. (a) Translated partial *PawL1b* transcript of *Zinnia haageana* color coded to indicate ER signal (rose), PLP (cyan), predicted albumin small subunit (green), and partial large albumin subunit (orange). (b) PLP‐1 from *Zinnia haageana*, encoded by *PawL1*. (c) Distribution of PLPs in the tribes of the Asteroideae (green shading indicates evidence of PLPs and *PawL1* transcripts, yellow shows *PawL1* transcripts present, blue means no transcriptomes available). Selected subfamilies of the Asteraceae shown in large type. The numbers on each branch represent the number of transcriptomes assembled

In our previous work, Jayasena et al. (2017) extracted PLP‐1 and PLP‐2 using a less polar extraction method than that used routinely for the larger and Cys‐containing PDPs. The conservation of the corresponding region among the predicted proteins encoded by *PawL1* genes indicated that these were the first of a large family. From the transcriptomes of 110 species from throughout the Asteraceae, 168 *PawL1* genes were discovered in 50 species. Species containing *PawL1* genes were limited to the Asteroideae, with no evidence found in two other major Asteraceae subfamilies, the Cichorioideae and Mutisioideae (Figure 2). Analyzing the LC‐MS data of peptide extracts from species containing *PawL1* genes uncovered a number of masses consistent with novel PLPs. To acquire sequences for these masses, we fragmented the putative protonated PLPs by MS/MS and manually sequenced the peptides from the resulting mass spectra.

### 2.2 Peptides sequenced by LC‐MS/MS

The system of ion nomenclature used here for the MS/MS spectra of cyclic peptides is based on that of Ngoka and Gross (1999b), taking into account that the fragmentation of cyclic peptides begins with a single ring‐opening event. Each fragment ion has a label of the form *x*_*nJZ*_, where *x* is the ion type (a or b), *n* is the number of amino acid residues in the ion, *J* is the one‐letter code of the N‐terminal residue at the point of cleavage of the ring, and *Z* is the C‐terminal residue at the cleavage point. Ngoka and Gross (1999b), however, give no guidance on nomenclature where a cyclic peptide has repeated pairs of amino acids, leading to ambiguity in the cleavage point; we have therefore added the number of each residue in the sequence (as defined by linear translation of the gene transcript) in such cases.

**Figure 3:**
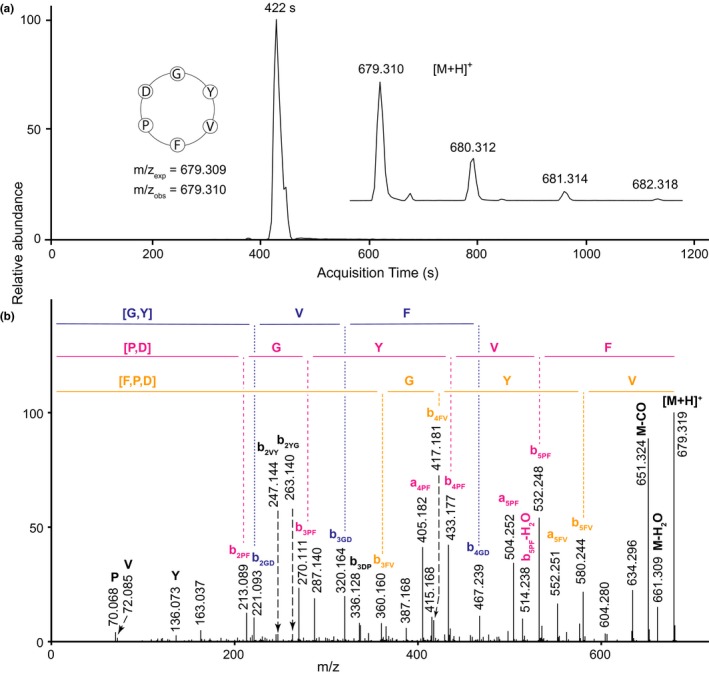
Q‐TOF LC‐MS data for PLP‐21 in *Inula racemosa*. (a) Extracted ion chromatogram showing acquisition time of the peptide, with (inset left) peptide sequence with expected and observed mass‐to‐charge ratios (m/z) and (inset right) peptide mass spectrum. (b) Tandem mass spectrum of the fragmented precursor ion. Sequences represented by selected b‐ion series are shown above the mass spectrum. [M+H]^+^ is the singly charged precursor ion; also shown are the precursor with loss of H_2_O (M‐H_2_O) or CO (M‐CO). Immonium ions are denoted by the one‐letter code of the residue they represent

The LC‐MS/MS data for peptide PLP‐21 from *Inula racemosa* (Figure 3) contained the ion series b_2PF_‐b_5PF_, together with the protonated molecule [M+H]^+^, which gives the residue sequence [P,D]‐G‐Y‐V‐F, the series b_3FV_‐b_5FV_ gives [F,P,D]‐G‐Y‐V and b_2GD_‐b_4GD_ gives [G,Y]‐V‐F (where the residues in square brackets are in unknown order). Together, these support the sequence cyclo‐F‐[P,D]‐G‐Y‐V, in agreement with the sequence GYVFPD from the *PawL1b* transcript of *I. racemosa*.

**Figure 4:**
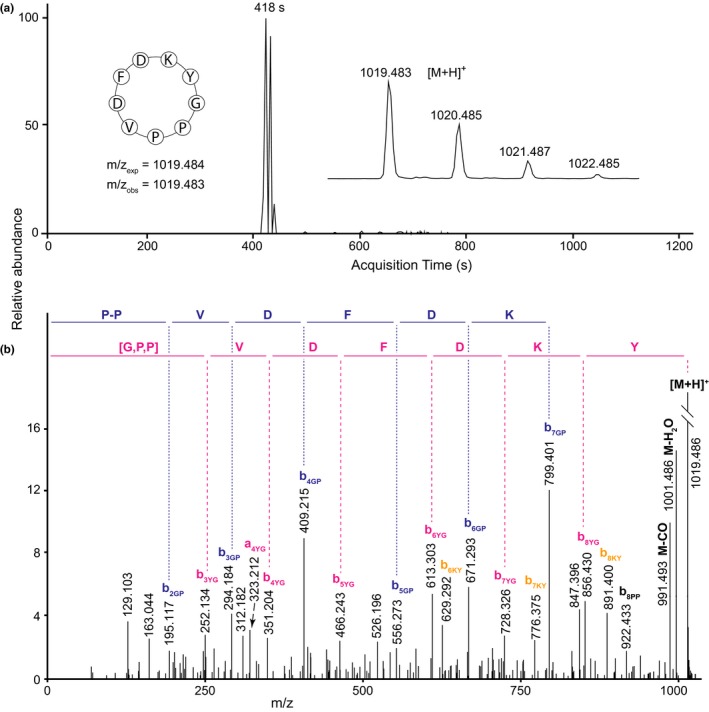
Q‐TOF LC‐MS data for PLP‐24 in *Othonna arborescens*. (a) Extracted ion chromatogram showing acquisition time of the peptide, with (inset left) peptide sequence with expected and observed mass‐to‐charge ratios (m/z) and (inset right) peptide mass spectrum. (b) Tandem mass spectrum of the fragmented precursor ion. Sequences represented by selected b‐ion series are shown above the mass spectrum. [M+H]^+^ is the singly charged precursor ion; also shown are the precursor ion with loss of H_2_O (M‐H_2_O) or CO (M‐CO)

The LC‐MS/MS data for peptide PLP‐24 from *Othonna arborescens* (Figure 4) displayed the ion series b_2PG_‐b_7PG_ confirming the residue sequence P‐P‐V‐D‐F‐D‐K and the series b_3GY_‐b_8GY_, with the [M+H]^+^ ion, providing the sequence [G,P]‐P‐V‐D‐F‐D‐K‐Y. Together, these form cyclo‐G‐P‐P‐V‐D‐F‐D‐K‐Y, matching the sequence KYGPPVDFD from the *PawL1b* transcript of *O. arborescens*.

A similar analysis of the LC‐MS/MS data from other species of the Asteroideae (Figures S1–S48) revealed another 42 unique PLPs making a total of 46 unique PLPs known across 17 species (Table 1). In this work, 50 PLPs were sequenced as six PLPs were found in either two species or subspecies. Of the 17 species containing PLPs, most contained more than one. Only five species contained a single detectable PLP, namely *Dahlia variabilis*,* Cosmos bipinnatus*,* Engelmannia peristenia*,* Arnica chamissonis*,* and Parthenium argentatum*. Of these five species, all except *A. chamissonis* had more than one *PawL1* gene so they may well have more than one PLP, but they fall below our limit of detection. Overall, these findings suggest most species contain multiple *PawL1* copies and multiple PLPs.

**Table 1:** List of PawL‐derived peptides. Listed by name and including sequence (as encoded), mass (monoisotopic), and genetic origin. The column on the right indicates the supplemental figure (or other location) where the LC‐MS data for each peptide are shown

Name	Encoded sequence	Mass (mono.)	Gene	Species	Supp. fig. no.
PLP‐1	AIIPGLID	792.475	*PawL1b*	*Zinnia haageana*	Jayasena et al. (2017)
PLP‐2	DLFVPPID	896.464	*PawL1c*	*Senecio pinnatifolius* var *maritimus*	Jayasena et al. (2017)
			*PawL1c*	*Senecio pinnatifolius* ssp *latilobus*	1
PLP‐3	GSLVYQID	875.439	*PawL1a*	*Senecio pinnatifolius* var *maritimus*	2
			*PawL1d*	*Senecio pinnatifolius* var *maritimus*	2
			*PawL1a*	*Senecio pinnatifolius* ssp *latilobus*	3
PLP‐4	GLLGITD	669.370	*PawL1d*	*Senecio pinnatifolius* var *maritimus*	4
			*PawL1a*	*Senecio pinnatifolius* ssp *latilobus*	5
PLP‐5	GLFVD	531.269	*PawL1e*	*Senecio pinnatifolius* var *maritimus*	6
PLP‐6	FFDAAKID	907.444	*PawL1b*	*Senecio pinnatifolius* var *maritimus*	7
			*PawL1b*	*Senecio pinnatifolius* ssp *latilobus*	8
PLP‐7	GLLDVVD	711.380	*PawL1a*	*Senecio pinnatifolius* var *maritimus*	9
PLP‐8	GYPPYYQD	983.402	*PawL1a*	*Zinnia haageana*	10
PLP‐9	GLLPPIID	818.490	*PawL1a*	*Zinnia elegans*	11
PLP‐10	GSPLFD	616.286	*PawL1b*	*Zinnia elegans*	12
PLP‐11	GVYPLGD	701.338	*PawL1b*	*Zinnia elegans*	13
PLP‐12	FVGGTSFD	810.355	*PawL1b*	*Senecio vulgaris*	14
PLP‐13	TFGVVIAD	802.422	*PawL1c*	*Senecio vulgaris*	15
PLP‐14	FVDTTGYD	898.371	*PawL1c*	*Senecio vulgaris*	16
PLP‐15	ALVVGLD	667.390	*Pawl1d*	*Senecio vulgaris*	17
PLP‐16	GLFPYGPD	846.391	*PawL1b*	*Dahlia variabilis*	18
PLP‐17	GFPPYVD	775.354	*PawL1a*	*Buphthalum salicifolium*	19
PLP‐18	GAIPFPD	697.344	*PawL1b*	*Buphthalum salicifolium*	20
PLP‐19	GVLFFPD	775.390	*PawL1b*	*Buphthalum salicifolium*	21
PLP‐20	GYLFPD	692.317	*PawL1a*	*Inula racemosa*	22
			*PawL1a*	*Inula helenium*	23
PLP‐21	GYVFPD	678.301	*PawL1b*	*Inula racemosa*	Figure 3
PLP‐22	GLPPYVD	741.370	*PawL1c*	*Inula racemosa*	24
			*PawL1b*	*Inula helenium*	25
PLP‐23	YFEEYIHD	1,096.450	*PawL1a*	*Othonna arborescens*	26
PLP‐24	KYGPPVDFD	1,018.476	*PawL1b*	*Othonna arborescens*	Figure 4
PLP‐25	YYEEYIHD	1,112.445	*PawL1c*	*Othonna arborescens*	27
PLP‐26	GFPWAPWD	956.418	*PawL1c*	*Melampodium paludosum*	28
PLP‐27	AVEPWIPFD	1,054.512	*PawL1b*	*Melampodium paludosum*	29
PLP‐28	FVETTAGLLD	1,046.528	*PawL1e*	*Steirodiscus tagetes*	30
PLP‐29	GYFPVGVD	834.391	*PawL1 g*	*Steirodiscus tagetes*	31
PLP‐30	YIDPAIGKRFGD	1,332.683	*PawL1b*	*Cosmos bipinnatus*	32
PLP‐31	GVPFPLITHD	1,076.565	*PawL1b*	*Engelmannia peristenia*	33
PLP‐32	GVLPPMLD	822.431	*Pawl1a*	*Arnica chamissonis*	34
PLP‐33	GIIVPIVD	806.490	*PawL1b*	*Rudbeckia hirta*	35
PLP‐34	GIIIPIVD	820.506	*PawL1d*	*Rudbeckia hirta*	36
PLP‐35	GLKFPVVD	855.485	*PawL1e*	*Rudbeckia hirta*	37
PLP‐36	AILIPIVD	834.521	*PawL1e*	*Rudbeckia hirta*	38
PLP‐37	GFFPADGD	806.323	*PawL1 g*	*Rudbeckia hirta*	39
PLP‐38	GLYPYPD	805.365	*PawL1 g*	*Rudbeckia hirta*	40
PLP‐39	IIHLSTPFD	1,023.539	*PawL1j*	*Rudbeckia hirta*	41
PLP‐40	GILFPIAD	826.459	*PawL1 m*	*Rudbeckia hirta*	42
PLP‐41	GDVTSPFD	818.345	*PawL1a*	*Sanvitalia procumbens*	43
PLP‐42	TFFNPVID	933.460	*PawL1c*	*Sanvitalia procumbens*	44
PLP‐43	TLVIPIID	864.532	*PawL1d*	*Sanvitalia procumbens*	45
PLP‐44	GWGTPID	726.334	*PawL1d*	*Sanvitalia procumbens*	46
PLP‐45	GWITGPWD	912.413	*PawL1e*	*Sanvitalia procumbens*	47
PLP‐46	GYITPLD	759.380	*PawL1c*	*Parthenium argentatum*	48

### 2.3 Analysis of the primary structure of PLPs and their flanking precursor sequence

**Figure 5:**
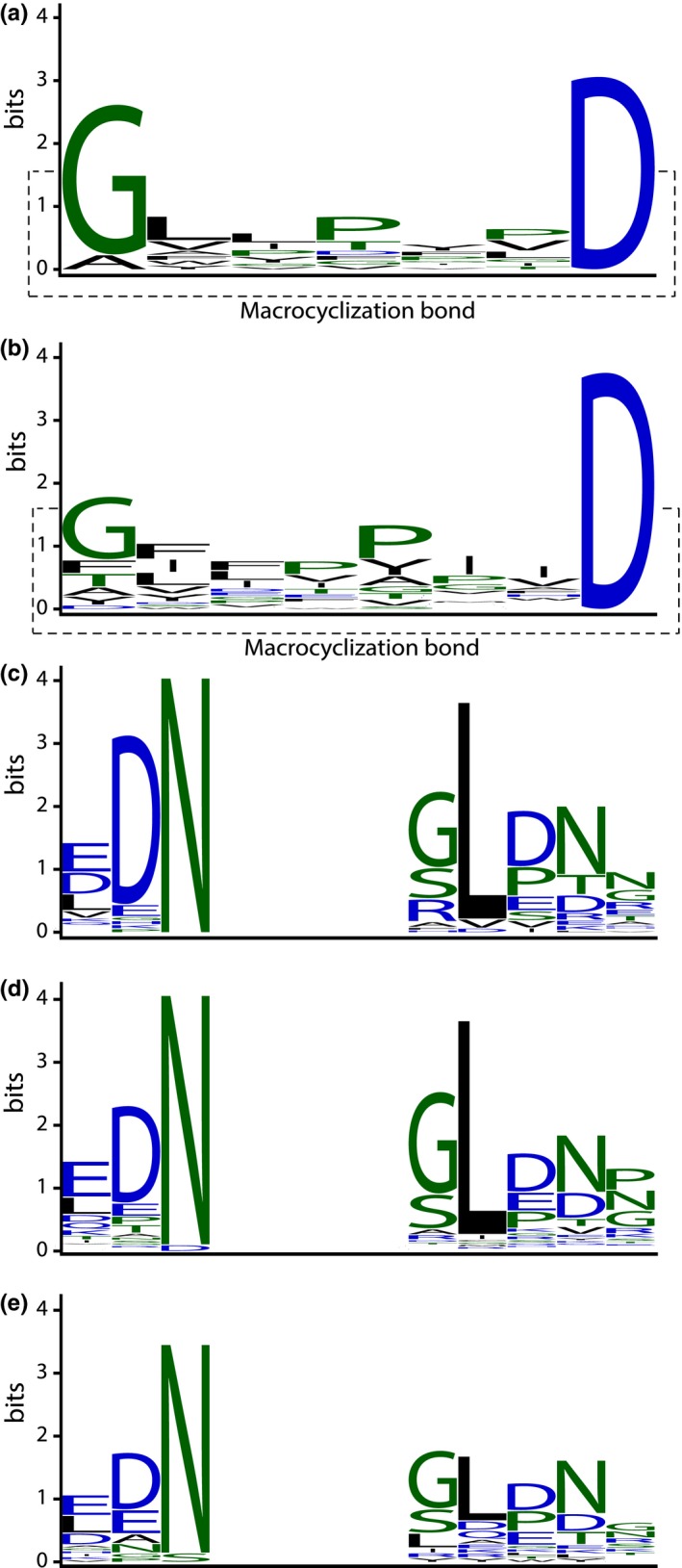
Sequence logo for PLPs consisting of (a) seven residues (*n* = 11) or (b) eight amino acid residues (*n* = 25). Together, these logos represent 78% (36 of 46) of PLPs, which range in size from five to twelve residues. Hydrophilic residues are shown in blue, hydrophobic in black, and neutral in green. Image created with WebLogo (Crooks et al., 2004). (c) The region flanking PLPs encoded by *PawL1* genes (*n* = 46) is conserved, indicating PLPs are matured in a similar fashion to PDPs. The absolutely conserved Asn (N) preceding each PLP is recognized by AEP, and the residues trailing the PLP region are consistent with what is required for macrocyclization reactions, especially the P2′ Leu. (d) Flanking region for putative PLPs with C‐terminal Asp which were not detected (*n* = 189). (e) Flanking region for putative PLPs without a C‐terminal Asp, none of which were detected (*n* = 23)

PawL‐derived Peptides were found to vary in length from five to twelve amino acid residues, with the majority having seven residues (11 PLPs) or eight residues (25 PLPs). PLPs contain an absolutely conserved Asp residue, and 28 of the 45 PLPs contain a Gly residue at the first position, which is bonded to the Asp during transpeptidation to form the macrocycle. A few contain Phe, Thr, Tyr, or Ala in place of this Gly^1^; the Gly^1^ residue is substituted by Lys, Asp, or Ile in one case each. Some PLPs contain the Pro‐Pro hinge which is a common feature of the PDPs. Most of the residues found in PLPs are hydrophobic, with Ile, Leu, Val, Pro, and Phe being especially common. The distribution of amino acids in each position for those PLPs containing seven or eight residues is shown in a sequence logo (Figure 5). A small number of PLPs contain polar residues such as Arg, Ser, Thr, Glu, Gln, and His, but these residues are not common across the family as a whole. The amino acid Trp is present in only two species and Met in one.

The sequence flanking each PLP we detected is conserved (Figure 5c). The residue immediately preceding the PLP region is an absolutely conserved Asn residue, which is the target of AEP. Trailing each PLP within the precursor is a conserved sequence similar to PDPs (Jayasena et al., 2017). The P1′ residue is most often one with a small side chain (Gly, Ser), but Arg and other residues are also found here. The P2′ residue is typically a more highly conserved Leu residue that, in some systems where macrocyclization is conducted by AEP, has been shown to be essential to produce a cyclic product (Conlan et al., 2012; Mylne et al., 2012). The P3′ and P4′ residues are ones that typically precede the small subunit of seed storage albumin, which is another point of maturation by AEP. Although hydrophobic like known orbitides, the PLPs are unique due to their omnipresent Asp that serves as its cyclization point by AEP. The flanking sequences for putative PLPs we could not detect still showed similar conservation to regions in transcripts for PLPs we could detect (Figure 5d,e).

### 2.4 *PawL1* and PLPs are absent from the family Corymbioideae

Corymbioideae is a small subfamily in the Asteraceae whose phylogenetic position is controversial; it is currently thought to predate the Asteroideae, but appeared following the divergence of the Cichorioideae and Mutisioideae. To determine whether PLPs are restricted to the Asteroideae, we obtained seeds of four different Corymbioideae species, extracted their RNA, assembled their transcriptomes de novo, and checked the quality of each assembly (Table S1). Searching these transcriptomes as previously described (Jayasena et al., 2017), we were unable to find any candidate *PawL1* genes, although the presence of seed storage albumin genes and a set of six “core genes,” as described by Jayasena et al. (2014) indicated that the transcriptome assembly would have been adequate to find any *PawL1* genes present (Table S2). As *PawL1* genes are confined to the Asteroideae, it indicates the age of this gene type is 45 million years.

### 2.5 Structural analysis of PLPs

We searched the Protein Data Bank and were unable to find any structures for orbitides, despite orbitide secondary structures being published in the literature, prompting us to solve the solution structures for a number of different PLPs using NMR spectroscopy. As it was not feasible to extract sufficient native peptide for these studies, we analyzed synthetic peptides produced by solid‐phase peptide synthesis. These peptides were cyclo‐DLFVPPID (PLP‐2), cyclo‐GLLGITD (PLP‐4), cyclo‐GSPLFD (PLP‐10), and cyclo‐FVGGTSFD (PLP‐12), which were chosen as they represented a range of different sequences, amino acid types, and molecular masses. Each synthetic peptide was subjected to LC‐MS/MS and confirmed to be identical to its native counterpart by matching retention time in LC, molecular mass, isotope profile, and MS/MS fragmentation pattern (Figures S49–S52).

**Figure 6:**
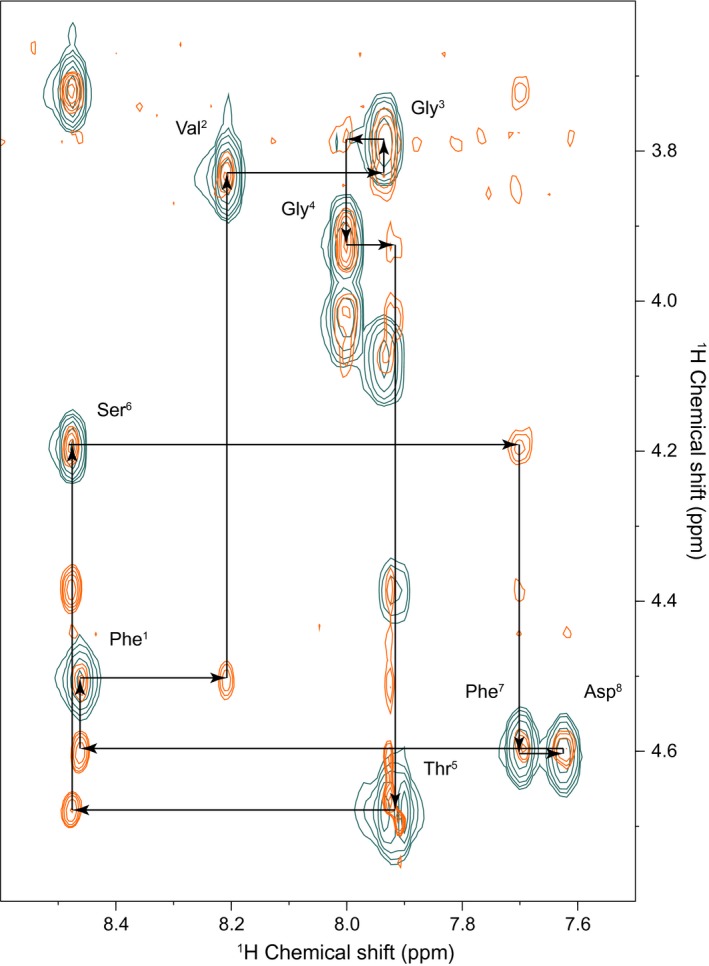
Sequential walk of the fingerprint region of ^1^H‐^1^H TOCSY (blue‐green) and ^1^H‐^1^H ROESY (orange) spectra of PLP‐12 (cyclo‐FVGGTSFD) from *Senecio vulgaris*, identifying its residue sequence

The amino acid residues in synthetic PLP‐12 were identified by a sequential walk of the H_α_ and H_N_ shifts of its ^1^H‐^1^H TOCSY and ROESY spectra (Figure 6). The same procedure was followed for synthetic PLP‐2, PLP‐4, and PLP‐10 (Figures S53–S56). Following this, ROESY peaks (NOESY in the case of PLP‐2) were identified and used as restraints for structural modeling.

**Figure 7:**
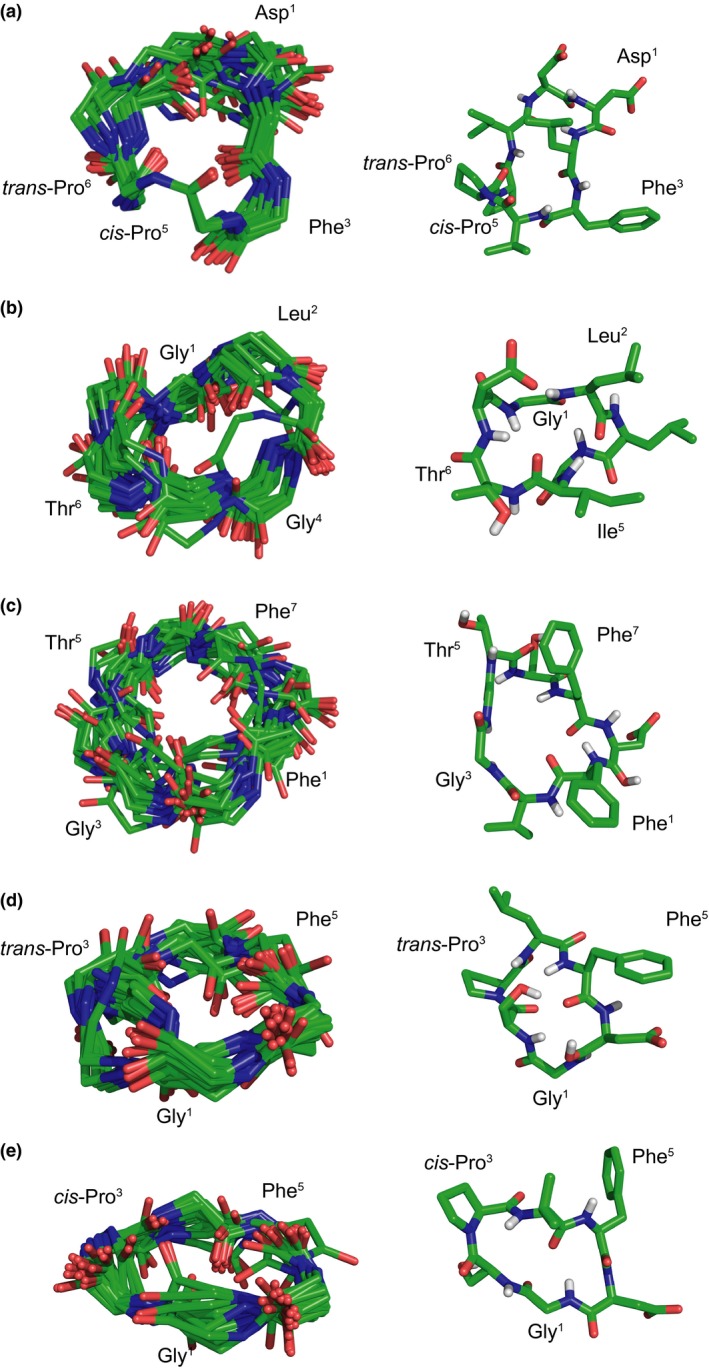
NMR solution structures of PLPs. NMR solution backbone structure ensembles (left) and most representative structures (right) for four PLPs colored by element (green = C, blue = N, red = O, white = H. Only polar hydrogen atoms shown). The most representative structure is the one closest to the mean of the ensemble, as measured by RMSD. (a) PLP‐2 (cyclo‐DLFVPPID), (b) PLP‐4 (cyclo‐GLLGITD), (c) PLP‐12 (cyclo‐FVGGTSFD), (d) PLP‐10 (cyclo‐GSPLFD) *trans*‐Pro conformer, and (e) PLP‐10, *cis*‐Pro conformer

Ensembles of the lowest energy 20 structures for each peptide were calculated and included the ensemble member closest to the mean, as measured by root mean square deviation (RMSD) (Figure 7). None of the peptides displayed evidence of classical secondary structure, even though several displayed strong deviation of their chemical shifts from random coil values (Figure S57).

Structure statistics for all the peptides were calculated and reported (Table S3). Analysis of PLP‐2, which was soluble only in DMSO, showed *cis* Val‐Pro and *trans‐*Pro‐Pro bonds in its major form in solution, with other conformations present only to a minor degree (Figure 7); the ensemble backbone RMSD was 0.5 Å. PLP‐4 and PLP‐12 had ensemble backbone RMSDs of 0.8 and 0.9 Å, respectively.

A notable feature of PLP‐10 was that it adopted two conformations in solution, differing by whether the Ser^2^‐Pro^3^ bond was in a *trans* (Figure 7d) or *cis* (Figure 7e) orientation. The NMR spectra showed both conformations to be present in approximately equal proportions. The backbone RMSD for the *trans* form was 0.6 Å, whereas the *cis* conformer had an RMSD of 0.5 Å. These data indicate that the PLPs examined exhibit a constrained and flat conformation.

### 2.6 PLPs show no antibacterial or antifungal activity

Although some studies on orbitides have indicated they possess antibiotic activity, an inspection of LB agar plates after overnight incubation at 37°C showed no inhibition of the growth of the Gram‐negative bacteria, *Escherichia coli* B and *Pseudomonas aeruginosa*, nor of the Gram‐positive bacteria, *Bacillus cereus* and *Staphylococcus aureus*, by any of six disks containing between 0.5 μg and 25 μg of synthetic PLP‐2, PLP‐18, or PLP‐20, nor did a negative control disk, treated only with water, inhibit growth (Figure S59A–D). By contrast, there was a clear zone of growth inhibition around a positive control disk containing kanamycin, except in the case of *P. aeruginosa*, which is known to be more resistant to kanamycin than the other bacterial species (Morita, Tomida, & Kawamura, 2014). However, a separate control plate with disks containing larger amounts of kanamycin did show growth inhibition of *P. aeruginosa* (Figure S59E).

Although some orbitides have been claimed to be antifungal, we found no difference in growth of three different fungi in wells of a 96‐well plate containing a culture only and those also inoculated with the synthetic PLP‐12 at any of nine concentrations between 1,250 and 9.8 μg/ml. There was no growth in a series of no inoculum‐negative controls.

## 3 DISCUSSION

Elliott et al. (2014) and Jayasena et al. (2017) identified 168 *PawL1* genes in 50 Asteroideae species, which appear to encode ~230 unique PLP sequences. We were unable to work on five species previously studied by Jayasena et al. (2017) because their import into Western Australia is prohibited, and another five because specimens were no longer on hand; these ten excluded species contained a total of 29 *PawL1* genes. In a search of the remaining 40 species, 44 unique PLPs matching 39 *PawL1* genes were detected at the peptide level in 17 species in this study, in addition to the two peptides identified by Jayasena et al. (2017). Combined this brings the total number of unique PLPs to 46.

Of the undetected peptide sequences, 23 did not include a proto‐C‐terminal Asp, whereas this Asp was absolutely conserved in all the observed peptides; this supports the hypothesis that PLPs, such as PDPs, are processed by an AEP that cleaves only when an Asp is present in the P1 position. The rarity of these sequences and their confinement within a phylogenetic span of species that have PLPs lead us to hypothesize that these genes lacking a proto‐C‐terminal Asp were likely to have been PLP‐containing proteins at one point, but have lost their PLP‐coding capability by random mutation in or near the sequence encoding the proto‐C‐terminal Asp. Most are still open reading frames and may well still make functional seed storage albumins.

Other than the conserved Asp, there was no discernible pattern as to which PLPs were detected by mass spectrometry. We observed that some predicted peptides were found in one species, but not in a closely related one. For example, transcript data predicted the presence of PLP‐32 in both *Arnica montana* and *A. chamissonis*, but PLP‐32 was found only in the latter. It is also noteworthy that PLPs found in a particular batch of seeds were sometimes undetectable in another batch of the same species and variety. Although cyclic peptide expression in plants is strongly influenced by environmental factors (Gui et al., 2012; Narayani, Chadha, & Srivastava, 2017), being coupled to seed storage albumin precursors means the more likely explanation for this is that in some species the peptides are close to the detection limit. It is therefore likely that at least some of the “missing” peptides do exist, but were either not expressed or expressed at levels in our seed samples below the limit of detection.

### 3.1 Sequencing of PLPs by LC‐MS/MS

Sequencing of cyclic peptides is challenging because MS/MS fragmentation may initiate at any peptide bond in the ring leading to a multiplicity of ions, the number of which is proportional to the square of the number of amino acid residues present. However, in those cases where the peptide contains a Pro residue, ring cleavage tends to occur predominantly at the N‐terminal side of that amino acid (Tomer, Crow, Gross, & Kopple, 1984), simplifying analysis of the mass spectrum, although collision energy may also influence this process (Ngoka & Gross, 1999a). Examples of PLPs that undergo this type of cleavage are PLP‐1 and PLP‐2 (Jayasena et al., 2017; Figure S1), as well as PLP‐9, 11, 17, 31, 33–38, 40, 41, and 43–46 (Figures S11, S13, S19, S33, S35–S40, S43, S44, S45–S48). In other cases, sequencing required careful analysis of multiple ion series from several ring‐opening events, none of which was strongly favored over another (Eckart, Schwarz, Tomer, & Gross, 1985). We did find cleavage at an Ile‐Asp or Leu‐Asp bond was often preferred when present, as in PLP‐3 (Figures S2–S3), PLP‐6 (Figures S7–S8), and PLP‐15 (Figure S17), although there were exceptions to this (PLP‐28, Figure S30; PLP‐30, Figure S32). It is perhaps worth noting that PLP‐28 and PLP 30 are the two largest PLPs, with 10 and 12 residues, respectively.

Some authors have suggested multistage tandem mass spectrometry (MS^*n*^) as an alternative sequencing method for cyclic peptides (Jia, Qi, He, & Qiao, 2006; Ngoka & Gross, 1999a), but we found that ion abundance beyond MS^3^ was too low to usefully employ stepwise sequencing; even though MS/MS sequencing was sometimes difficult, it remained our preferred method. Our task was made easier by having access to transcriptomic data, allowing us to predict what ions would be present in the mass spectra and to distinguish between isobaric Ile and Leu residues.

Although we matched four synthetic, head‐to‐tail cyclized PLPs to the native PLP in LC‐MS and MS/MS, it has been assumed based on conservation that all PLPs are head‐to‐tail cyclic. Some PLPs contain residues with amino groups in their side chain that could conceivably take the place of the proto‐amino terminus in the transpeptidation reaction performed by AEP. Most conspicuous of these is PLP‐24, which has a Lys residue at its proto‐N‐terminus. PLP‐6, PLP‐30, and PLP‐35 have internal Lys residues that, if side chain cyclized, would form “lasso” peptides of the same mass as backbone cyclic. With limited plant material, it was not possible to purify enough native material of these four to confirm they had the conserved, AEP‐mediated backbone cyclic structure as the four native PLPs for which a synthetic version was matched (Figures S49–S52). Several native PDPs have similarly been confirmed as backbone cyclic and for SFTI‐1 and SFT‐L1 from sunflower, native material was used to solve their solution structures (Elliott et al., 2014; Luckett et al., 1999; Mylne et al., 2011).

### 3.2 Phylogenetic distribution and evolution of PLPs

The first peptide found buried in a seed storage albumin, SFTI‐1, has been known for almost two decades (Luckett et al., 1999), but its genetic provenance was discovered only recently in the *PawS1* gene of *H. annuus* (Mylne et al., 2011). Further work revealed a family of such PDPs within the Asteroideae subfamily of the daisy family (Elliott et al., 2014; Jayasena et al., 2017). These peptides are distinguished by their backbone cyclization and a single disulfide bond. Jayasena et al. (2017) also discovered the first two members of a peptide family which is the ancestor of the PDPs; these small cyclic peptides were named the PLPs.

In this study, we investigated the distribution and properties of the PLPs showing that, like the PDPs, they are found in tribes within the Asteroideae subfamily, but have a wider distribution than the PDPs; the latter are concentrated in the Heliantheae tribe. Like the PDPs, PLPs are not found in other Asteraceae subfamilies, as evidenced by their absence in the Corymbioideae, the subfamily most closely related to the Asteroideae.

We found *PawL1* transcripts in 11 of 17 Asteroideae tribes we examined and peptide evidence of PLPs in six tribes (Figure 2): the Millerieae (1 of 5 species studied), Madieae (1 of 2), Heliantheae (6 of 13), Coreopsideae (2 of 7), Inuleae (3 of 5), and Senecioneae (4 of 9). These results show no clear evolutionary pattern; some tribes closely related to those containing PLPs appear to contain none, in contrast to more distant relatives that also contain PLPs. This could be due to the small numbers of species sampled in some tribes. For example, the largest concentration of PLPs was found in the Heliantheae, Inuleae, Coreopsideae, and Senecioneae tribes, yet none were found in the closely related Tageteae and Helenieae. This may be due to only five species in total being sampled in the last two tribes. Further studies on a larger number of species in each tribe are necessary to more clearly delineate the distribution of PLPs in the Asteroideae, which contains ~17,000 species.

### 3.3 NMR solution structures

In a search of the Protein Data Bank, we found no tertiary structures of orbitides, although some studies looking at secondary structure have been published, namely NMR studies with ribifolin (Pinto et al., 2015), cherimolacyclopeptide A (Wélé, Zhang, Ndoye, et al., 2004), annomuricatin A (Wélé, Zhang, Caux, et al., 2004), cycloreticulin B (Wélé et al., 2008), mahafacyclin A (Baraguey et al., 2000), and segetalins D and E (Morita, Yun, Takeya, & Itokawa, 1997). Other orbitide structural information has been obtained by X‐ray diffraction, such as for cycloreticulin A (Wélé et al., 2008), annomuricatin A (Wu et al., 2007), and squamin A (Jiang, Lu, Min, & Zheng, 2003). Both solution and crystal structural studies have been carried out on pseudostellarin D (Morita, Kayashita, Takeya, & Itokawa, 1996), yunnanin A (Morita, Kayashita, Takeya, Itokawa, & Shiro, 1997), and evolidine (Eggleston, Baures, Peishoff, & Kopple, 1991).

With no tertiary structures of orbitides available, we were interested in solving the structure of some PLPs in solution, to determine whether they shared secondary structural features with other orbitides or had any distinct structural features which would give them differing properties.

In contrast to other orbitides, which display small structural motifs in solution, such as β turns and β bulges (Baraguey et al., 2000; Wélé, et al., 2008; Wélé, Zhang, Caux, et al., 2004; Wélé, Zhang, Ndoye, et al., 2004) or γ turns (Pinto et al., 2015), we found no evidence of secondary structure in the four peptides studied, despite some NMR chemical shifts displaying deviations from random coil values (Figure S57). A previous study found that cyclic peptides with six, ten, or 14 residues often contain small secondary structure motifs, whereas those with eight, 12, or 16 do not (Gibbs et al., 1998). The peptides we studied contained six, seven, or eight residues, but we found no evidence of classical α or β structural elements, nor of any small structural motifs such as turns and bulges.

Contact maps of NOESY or ROESY interactions between residues show few long‐range interactions in any of the peptides studied (Figure S58). PLP‐2 has more contacts between nonsequential residues than the other peptides (Table S3) and also has the lowest backbone RMSD. The Pro‐Pro hinge is likely responsible for the greater degree of order in this peptide. Although PLP‐4 has the next largest number of nonsequential contacts, its backbone RMSD is greater than that of PLP‐10 in either *trans* or *cis* conformation. Again this may be due to the stability conferred by the Pro residue, which is present in PLP‐10 but not in PLP‐4. PLP‐12 also has more nonsequential contacts than PLP‐10 but lacks a Pro residue; it has the largest backbone RMSD of the four peptides.

### 3.4 Antibacterial and antifungal assays

The literature contains a number of studies of orbitides that have a diverse range of activities when tested in vitro; recent examples include antifungal (Tian et al., 2010), antimalarial (Pinto et al., 2015), and cytotoxic activities (Beirigo et al., 2016; Cândido‐Bacani, Figueiredo, Matos, Garcez, & Garcez, 2015; Okinyo‐Owiti, Young, Burnett, & Reaney, 2014). Such activities might be useful as a defensive mechanism for plant seeds. We tested several synthetic PLPs in assays against bacteria and fungi, but found none had effects on their growth in our experiments. As most Asteroideae species produce more than one PLP, it is possible that they could act as multicomponent antimicrobials that only work in combination, but not on their own. This behavior has been observed in the two‐component bacteriocins produced by lactic acid bacteria (Garneau, Martin, & Vederas, 2002), in which two peptides act synergistically against other Gram‐positive bacteria. They can be divided into two classes: the lantibiotics, which comprise two ribosomally synthesized and heavily post‐translationally modified lanthipeptides, and the nonlantibiotics, which contain few post‐translational modifications other than disulfide bonds. These bacteriocins differ from the PLPs in that they are not head‐to‐tail cyclic and tend to be larger in size (30–60 amino acids). With limited seed material, we had to synthesize PLPs to acquire sufficient peptide to characterize them and so were unable to perform these assays with PLPs in combination.

### 3.5 Comparison with other orbitides

The orbitides are a class of backbone cyclic peptides containing between five and 12 amino acid residues. They do not contain disulfide bonds and are ribosomally synthesized (although the genes encoding orbitides are known only in a few instances). Following discovery of the PLPs, orbitides are now found in nine plant families, with the majority of known peptides concentrated in the Caryophyllaceae (hence their earlier name: Caryophyllaceae‐type peptides) (Table 2).

**Table 2:** Known orbitides. The Asteraceae orbitides are all PLPs

Plant family	Known orbitides	Cyclization enzyme	Size range	Known precursor	NMR analyses	X‐ray structures
Asteraceae	46	AEP	5–12	46	4	—
Caryophyllaceae	104	PCY1	5–11	6^a^^,^ b	—	—
Annonaceae	28	Unknown	6–9	—	3	3
Rutaceae	17	Unknown	6–8	3^a^^,^ b	—	—
Euphorbiaceae	20	Unknown	7–12	—	2	—
Lamiaceae	10	Unknown	6–12	—	—	—
Linaceae	17^e^	Unknown	8–10	11^a^^,^ c ^,^ d	—	—
Solanaceae	4	Unknown	8	—	—	—
Santalaceae	1	Unknown	5	—	—	—

a Covello et al. (2010).

b Condie et al. (2011).

c Okinyo‐Owiti et al. (2014).

d Burnett et al. (2015).

e Excludes variants containing modified amino acids, such as methionine sulfoxide.

In *Linum usitatissimum* (Linaceae), the genes encoding the cyclolinopeptides contain multiple copies of a single orbitide sequence or closely related sequences (Burnett, Jadhav, Okinyo‐Owiti, Poth, & Reaney, 2015; Covello et al., 2010; Okinyo‐Owiti et al., 2014), whereas orbitide genes in the Caryophyllaceae and Rutaceae are short and encode only a single peptide (Condie et al., 2011; Covello et al., 2010). The PLPs differ from these in that their sequences are buried within genes that also code for a completely unrelated protein, namely a napin‐type seed storage albumin.

Although the amino acid sequences of PLPs are similar to those of other orbitides, there is one striking difference: whereas Asp and Glu are rare in most orbitides (<1% of total residues each), the PLPs contain an absolutely conserved Asp. As the PLPs rarely contain basic residues, this makes nearly all PLPs acidic. As with PDPs, kalata‐type cyclic peptides and cyclic knottins, the linear peptide sequences of PLPs often contain an N‐terminal Gly residue, which may be a consequence of Gly facilitating cyclization by AEP (Bernath‐Levin et al., 2015), although *Citrus* orbitides and the segetalins also favor Gly in this position; cyclolinopeptides, however, rarely contain a Gly residue at the N‐terminus of their linear sequences.

A common feature of the PLP family and other orbitides is that the following hydrophobic residues each amount to more than 5% of the total amino acid abundance in both groups: Gly, Phe, Ile, Leu, Pro, Val, and Tyr. The residue Ala is also common (4.0% of PLP residues and 6.1% for other orbitides).

The absolutely conserved Asp in PLPs suggests that, like PDPs and cyclotides, PLPs are cleaved from the corresponding precursor by an AEP which also performs the cyclization reaction by transpeptidation (Bernath‐Levin et al., 2015; Gillon et al., 2008; Mylne et al., 2011; Nguyen et al., 2014; Saska et al., 2007). AEP cleaves at a conserved Asn before the N‐terminus of the peptide and either the same or another AEP cleaves the conserved Asp at the C‐terminus and ligates the two termini into a macrocycle (Bernath‐Levin et al., 2015; Nguyen et al., 2014). It is likely that two different AEPs catalyze PLP formation; one favoring cleavage at the Asn residue preceding the peptide sequence and another at the proto‐C‐terminal Asp; prior work has shown that particular AEPs tend to favor one of these residues over the other (Bernath‐Levin et al., 2015; Harris et al., 2015; Nguyen et al., 2014). A C‐terminal recognition sequence (consensus GLDN) is necessary for this reaction to take place (Bernath‐Levin et al., 2015). This contrasts with the segetalin orbitides of *V. hispanica*, which are cleaved at the N‐terminus by an oligopeptidase, and cleaved at the C‐terminus and cyclized by the enzyme PCY1 (Barber et al., 2013). These reactions require both an N‐terminal sequence and a C‐terminal recognition sequence, whose consensus sequences are MSPILAHDVVKPQ and AKDVENASAPV, respectively (Condie et al., 2011). Similarly, the linear peptide precursors of orbitides in *Citrus* species (Rutaceae) and *Linus usitatissimum* (Linaceae) contain conserved N‐terminal and C‐terminal sequences bracketing the orbitide sequence, suggesting these peptides may be processed in a manner similar to the segetalins (Condie et al., 2011; Covello et al., 2010; Okinyo‐Owiti et al., 2014). This implies a difference between the processing of the PLPs (which have more in common with PDPs) and orbitides in other plant families.

The aforementioned differences, notably the absolutely conserved Asp in the PLPs, their different method of post‐translational processing, and the buried nature of the peptide alongside an unrelated protein denote the PLPs as a distinct class of orbitide. We have used a transcriptome‐guided and MS/MS‐based approach to unearth 44 new members of a potentially very large family of homodetic cyclic peptides. This means that, with 46 PLPs, the Asteraceae contains more known orbitides than any plant family other than the Caryophyllaceae and due to the size and rapid recent evolution of the Asteraceae, the PLP family has the potential to have members numbering in the thousands. We have also greatly expanded the number of peptides known to be buried within protein precursors; it is becoming ever more apparent that evolution of peptides within existing protein hosts is an important phenomenon meriting further investigation.

## 4 METHODS

### 4.1 Synthetic peptides and seed peptide extraction

Synthetic peptides cyclo‐GLLGITD (PLP‐4), cyclo‐GSPLFD (PLP‐10), and cyclo‐GYLFPD (PLP‐20) were obtained from Purar Chemicals (Melbourne, VIC, Australia). All other synthetic peptides were purchased from GenScript (Piscataway, New Jersey, USA). Peptides were synthesized to a purity ≥95%, except for PLP‐20, which was approximately 50% pure. Peptides from GenScript had trifluoroacetate residue removed and replaced by formate.

Seed peptides were extracted as described by Jayasena et al. (2017). Briefly, about 1 ml of seeds was ground with mortar and pestle and peptides extracted with a 4:4:1 mixture of methanol, dichloromethane and 0.05% (v/v) trifluoroacetic acid. Phases were separated by alternate addition of chloroform and 0.05% trifluoroacetic acid followed by centrifugation. The upper (aqueous) phase was dried in a vacuum centrifuge (Labconco), and then the dried peptide extracts were dissolved in 50–100 μl of HPLC‐grade water containing 5% (v/v) acetonitrile and 0.1% (v/v) formic acid (Honeywell).

### 4.2 LC‐MS/MS and peptide sequencing

Data collection using the Agilent Technologies 1100 or 1200 series HPLC system coupled to a 6510, 6520, or 6550 Q‐TOF mass spectrometer was performed using a method similar to that of Jayasena et al. (2017). Briefly, a gradient elution was run with HPLC‐grade solvents (Honeywell) from 5% solvent B to 95% solvent B over 15 min at a flow rate of 300 nl/min using a C18 high‐capacity nano‐LC chip (Part No. G4240‐62010, Agilent Technologies) comprising a trapping column (160 nl) and an analytical column (75 μm × 150 mm); solvent A was 0.1% (v/v) formic acid in water and solvent B was 0.1% (v/v) formic acid in acetonitrile. MS data were collected at one spectrum per second. LC‐MS/MS experiments were carried out at two scans per second, targeted to PLP masses predicted from transcriptome data. The putative peptides were fragmented using collision‐induced dissociation at voltages between 31 and 36 V. In some cases, the voltage was adjusted to optimize fragmentation.

For data collection using the Thermo Scientific Orbitrap Fusion, samples were analyzed by LC‐MS/MS on a Dionex UltiMate 3000 Nano‐UHPLC system (Thermo Scientific) using an EASY‐Spray PepMap C18 column heated to 35°C (75 μm × 150 mm, 3 μm particle size, 10 nm pores; Thermo Scientific) coupled to an Orbitrap Fusion mass spectrometer (Thermo Scientific). A gradient elution was run with HPLC‐grade solvents (Fisher Chemical) from 5% solvent B to 95% solvent B over 50 min at a flow rate of 200 nl/min; solvent A was 0.1% (v/v) formic acid in water and solvent B was 0.1% (v/v) formic acid in acetonitrile. “Microliter pickup” mode was used to inject 2–5 μl of each sample. The mass spectrometer was run in positive, data‐dependent, “top speed” MS/MS mode, with two alternating stepped HCD fragmentation energies to optimize fragmentation: 14% ± 3% and 25% ± 3%. Both MS and MS/MS scans were carried out in the Orbitrap mass analyzer with resolution set to 60,000; the MS scan range was from 400 to 1,600 m/z; MS/MS was carried out only for peaks above 1 × 10^5^ intensity (arbitrary units) with an isolation window of 1.6 m/z, an AGC target of 5 × 10^5^, minimum m/z 50, maximum injection time 250 ms, and 2 microscans. The source voltage was 1,800 V.

Peptides were sequenced de novo by visual examination of MS/MS spectra, aided by fragment predictions from the program mMass version 5.5.0 (Niedermeyer & Strohalm, 2012). The margin of error in fragment m/z accepted for Q‐TOF data was ±0.02, and for Orbitrap data, ±0.01. The parameters used for mMass allowed for fragmentation into a‐ions, b‐ions, and immonium ions. Permitted neutral losses were water or ammonia. Other parameters were set at their default values.

### 4.3 *Corymbium* spp. seed RNA extraction and transcriptome assembly

Seeds were ground with mortar and pestle to a fine powder under liquid nitrogen. One hundred milligrams of the powder was used for total RNA isolation using the Spectrum Plant Total RNA kit (Sigma‐Aldrich). For RNA‐seq analysis, libraries were prepared from the total RNA using the TruSeq Stranded Total RNA with Ribo‐Zero Plant kit according to the manufacturer's instructions (Illumina). Sequencing runs were performed on a NextSeq550 platform (Illumina), generating single‐end reads with a length of 150 bp and an average quality score (Q30) of above 95%.

Raw reads were preprocessed using the FASTX toolkit. Parameters were set to trim reads with quality threshold at 30 and minimum length at 50 (Q30, l50) and filter low‐quality reads with quality cut‐off at 22 and a minimum of 90% bases that must have 22 quality score (q22, p90). Filtered reads were then assembled de novo on CLC Genomics Workbench 10.0.3 (Qiagen), as previously described (Jayasena et al., 2014). Four different word sizes (i.e., 23, 30, 50 and 64) were assembled, keeping all other parameters as default. To check the quality of the assembled transcriptomes, we checked the coverage of six “core genes” selected for being ubiquitous among the Asteraceae and present in a wide range of abundance, following the method of Jayasena et al. (2014).

### 4.4 Sequence logo construction

As the majority of PLPs (36 of 46) contain seven or eight amino acid residues, we constructed two sequence logos (Schneider & Stephens, 1990): one with only seven‐residue PLPs and one with eight‐residue peptides. This allowed us to avoid bias introduced by sequence alignment, which inserts too many gaps into such short sequences. WebLogo version 2.8.2 was used (Crooks, Hon, Chandonia, & Brenner, 2004). We also constructed three sequence logos of the regions flanking the peptide: one for the PLPs that we could detect, one for undetected putative PLPs containing the highly conserved Asp residue found in all the detected peptides, and a third for undetected sequences that did not contain that Asp.

### 4.5 NMR spectroscopy and PLP structure determination

NMR spectroscopy data were collected with a 500 or 600 MHz Bruker Avance IIIHD NMR spectrometer equipped with a 5‐mm BBFO probe, using TopSpin software (v3.5; Bruker Daltonics). Peptides were dissolved to a concentration of 1.0–1.5 mg/ml in 90% H_2_O/10% D_2_O containing 50 μM 4,4‐dimethyl‐4‐silapentane‐1‐sulfonic acid (DSS) as a zero‐shift reference, except for cyclo‐GLLGITD, (PLP‐4) which was dissolved in 70% H_2_O/20% DMSO‐d6/10% D_2_O (v/v) with 50 μM DSS. A volume of 700 to 800 μl was contained in a 5‐mm NMR tube (Bruker). Spectra were measured at 25°C and pH 4.5 ± 0.5. With the exception of PLP‐2 (cyclo‐DLFVPPID), data were acquired using ^1^H TOCSY (20 ms and 80 ms spin‐lock mixing times), ^1^H DQF‐COSY, ^1^H ROESY (300 ms mixing time), and ^1^H‐^13^C HSQC.

Because PLP‐2 was not sufficiently soluble in water, spectra for this compound were obtained at a concentration of 2 mg/ml in DMSO‐d6 containing 50 μM DSS. Data were acquired using ^1^H COSY, ^1^H NOESY (300 ms mixing time), ^1^H TOCSY (80 ms spin‐lock mixing time), ^1^H‐^13^C HSQC, and ^1^H‐^13^C HMBC. PLP‐20 was not subjected to NMR analysis as it was insufficiently pure.

CcpNmr Analysis 2.4.2 (Vranken et al., 2005) was used to analyze and assign the NMR spectra of each peptide. Distance restraints were generated from NOESY or ROESY peak lists as single distance bins from 1.8 to 6.0 Å. A single distance bin was used to account for spin diffusion that can be evident in closed cyclic structures. Distance restraints created in CcpNmr Analysis were exported in CNS/XPLOR format and used for structural modeling in YASARA Structure version 16.7.22 (YASARA Biosciences) by running the predefined macros *nmr_fold* to generate a starting ensemble of structures, and *nmr_refine* to perform *in vacuo* refinement followed by in solvent refinement. Initial ensembles were calculated from 50, 100, or 200 structures, which were subsequently refined and solvent minimized. The 20 lowest energy structures were selected for the final ensemble. Ensemble average energy and restraint violations were calculated using the macro *nmr_analyze*. Ramachandran statistics and clashscore were generated with MolProbity (Chen et al., 2010) via the PSVS website (Bhattacharya, Tejero, & Montelione, 2007). PSVS was also used to calculate backbone and heavy atom RMSDs.

### 4.6 Antibacterial activity assay

Plates were prepared with 25 ml LB agar and incubated overnight at 37°C to confirm their sterility. LB medium (5 ml) was inoculated with three to five single colonies from agar plates of the Gram‐negative bacteria *E. coli* B or *P. aeruginosa* (ATCC 19429), or the Gram‐positive bacteria *B. cereus* (ATCC 10876) or *S. aureus* (ATCC 25923), and incubated with shaking at 37°C overnight. A 50 μl aliquot of each overnight culture was used to inoculate fresh 5 ml aliquots of LB, and the cultures were incubated at 37°C with shaking for 2.5 hr. The final cultures were diluted with sterile saline to an OD_600_ of 0.1. This turbidity level was established by colony count to fall within the range of 1 × 10^7^ to 2 × 10^7^ colony‐forming units (CFU)/ml for the Gram‐positive strains, and 5 × 10^7^ to 7 × 10^7^ CFU/ml for the Gram‐negative strains. Standardized bacterial suspensions were seeded evenly onto sterile LB agar plates using a sterile swab. Synthetic peptides PLP‐2, PLP‐18, and PLP‐20 were dissolved in dimethyl sulfoxide to a concentration of 5 mg/ml; an aliquot of each of these solutions was diluted with water to a concentration of 1 mg/ml. Each solution was dispensed onto sterile 8 mm diameter filter papers in the following amounts: 0.5, 1.25, 2.5, 5, 12.5, and 25 μg. Two controls were set up as follows: one containing 5 μl of water, the other 10 μg of kanamycin. All the filter papers were allowed to dry completely before placing them on the inoculated LB plates previously prepared. The plates were incubated overnight at 37°C, after which they were inspected for bacterial growth.

### 4.7 Antifungal activity assay

Serial dilutions of synthetic PLP‐12 were prepared in water at the following concentrations: 2,500, 1,250, 625, 313, 156, 78.1, 39.1, 19.5, and 9.8 μg/ml. A culture of the filamentous fungus *Zymoseptoria tritici* was grown on a V8PDA (V‐8 juice, potato, dextrose, and agar) plate. Cultures of *Aspergillus fumigatus* and *Saccharomyces cerevisiae* were grown on YPD (yeast extract, peptone, dextrose) agar plates. Spores were harvested from each plate, dispensed into YPD medium, and diluted to an OD_600_ of 0.05. Sixteen aliquots (180 μl) of each fungal culture were dispensed onto 96‐well plates and 20 μl aliquots of one of the two cyclic peptides added at each of the eight concentrations. In addition, a set of eight no inoculum controls was set up for each peptide. The plates were incubated at 37°C (*A. fumigatus*) or 23°C (*Z. tritici* and *S. cerevisiae*). After 4 days, the plates were inspected for fungal growth.

### 4.8 Accession numbers

Peptide structures, NMR‐derived restraints, peak lists, and chemical shifts have been deposited with the Protein Data Bank (PDB) and the Biological Magnetic Resonance Bank (BRMB) under the following accession codes: PLP‐2: 6AXI (PDB), 30341 (BMRB); PLP‐4: 6AWM, 30338; PLP‐10: 6AZF, 30343 (*trans*) and 6AZG, 30344 (*cis*); PLP‐12: 6AWK, 30337. The raw reads for the *Corymbium* transcriptomes have been deposited with the Sequence Read Archive under accession SRP118817.

## AUTHOR CONTRIBUTIONS

J.S.M. conceived the study; M.F.F performed seed peptide extractions, analyzed transcriptomic data, and performed antifungal assays; M.F.F and A.W.D. performed antibacterial assays; M.F.F and N.L.T. performed LC‐MS/MS; M.F.F. and M.J.H. performed NMR and structural studies; O.B and J.W. extracted RNA, prepared libraries, and performed sequencing; J.Z. assembled transcriptomic data; B.B. and P.A.P. helped with PLP discovery; all authors analyzed the data; M.F.F. and J.S.M. wrote the manuscript with contributions from all authors.
