# Supplementary material for: A family of small, cyclic peptides buried in preproalbumin since the Eocene epoch
Source: Plant Direct. 2018 Feb 28;2(2):e00042. doi: 10.1002/pld3.42 (PMC6223261; doi:10.1002/pld3.42)
Supplement: Supplementary file 1 [file PLD3-2-e00042-s001.pdf]

SUPPLEMENTAL TABLES

Supplemental Table 1. Summary of RNA-seq data and assemblies by CLC Genomics. Filtered reads were assembled with four word sizes (23, 30, 50, and 64 ws) and the 64 word size transcriptomes gave the highest number of contigs among all four word sizes; The N50 is the value at which it can be said that half of the nucleotides in the transcriptome assembly belongs to contigs with this length or longer.

**Species**	**Read type; length**	**raw reads**	**filtered reads**	**Word Size**	**Contigs**	**N50**
C. africanum	Single-end; 150	77,866,705	61,353,656	64 ws	181,000	343
				23 ws	124,078	470
C. cymosum	Single-end; 150	76,354,311	62,893,285	64 ws	339,198	432
				23 ws	286,984	537
C. glabrum	Single-end; 150	88,280,319	72,580,001	64 ws	206,958	406
				23 ws	119,996	563
C. villosum	Single-end; 150	87,131,011	71,397,475	64 ws	166,263	445
				23 ws	101,265	626

Supplemental Table 2: Quality statistics for *Corymbium* transcriptome core gene assemblies. Quality statistics are shown for only two of four assemblies that differ in their word size (ws=23 or ws=64). Under each gene name the first line is the average coverage (the sum of the bases of the aligned part of all the reads divided by the length of the reference sequence) and the second line is the percentage coverage of the reference protein-coding sequence.

Species	ws	*LEA*	*OLE*	*AP*	*PR*	*CP-1*	*PK*
C. glabrum	23	5,341	16,004	1,990	8,693	156	135
		81%	92%	87%	58%	90%	100%
	64	5,246	15,972	3,133	9,363	139	81
		62%	64%	97%	100%	55%	100%
C. cymosum	23	345	32	57	30,312	146	99
		93%	92%	100%	70%	26%	97%
	64	318	521	57	38,591	195	95
		100%	98%	87%	100%	100%	100%
C. vilosum	23	10,450	20,997	3,931	2,166	111	119
		98%	82%	99%	100%	73%	33%
	64	5,991	5,598	138	2,556	392	101
		100%	81%	100%	100%	100%	100%
C. africanum	23	2,028	9	56	1,892	4,134	136
		100%	0%[a]	94%	100%	78%	100%
	64	2,532	2,333	56	480	119	163
		62%	100%	97%	100%	68%	100%

[a] The assembled sequence covered only the 3' untranslated region of the transcript.

Supplemental Table 3: NMR structure statistics. Summary of restraints included in the CYANA calculations. Root-mean-square deviation values were calculated over the entire structure. Stereochemical quality was assessed via MolProbity (Chen et al., 2010). Clashscore is the number of steric overlaps > 0.4 Å per 1,000 atoms.

	**PLP-2**	**PLP-4**	**PLP-10 (*trans*)**	**PLP-10 (*cis*)**	**PLP-12**
**Sequence**	cyclo-DLFVPPID	cyclo-GLLGITD	cyclo-GSPLFD	cyclo-GSPLFD	cyclo-FVGGTSFD
**Experimental restraints**					
Distance restraints:					
Intra-residue	37	54	32	26	34
Inter-residue sequential	24	23	14	12	24
Inter-residue non-sequential	7	4	0	1	2
Dihedral-angle restraints	2	0	0	0	0
Energy (kJ/mol)	-2538	-2161	-1584	-1623	-2193
Structure statistics					
Violations	0	0	0	0	0
Ramachandran favored, %	85.8	80.0	86.3	78.7	91.7
Ramachandran allowed, %	14.20	15.0	11.3	15.0	8.3
Ramachandran disallowed, %	0	5.0	2.5	6.2	0.0
Mean RMSD (Å) backbone	0.5	0.8	0.6	0.5	0.9
Mean RMSD (Å) heavy atoms	1.3	1.6	1.8	1.5	1.9
MolProbity clash score	1.2	1.0	1.2	0.6	0.0

SUPPLEMENTAL FIGURES

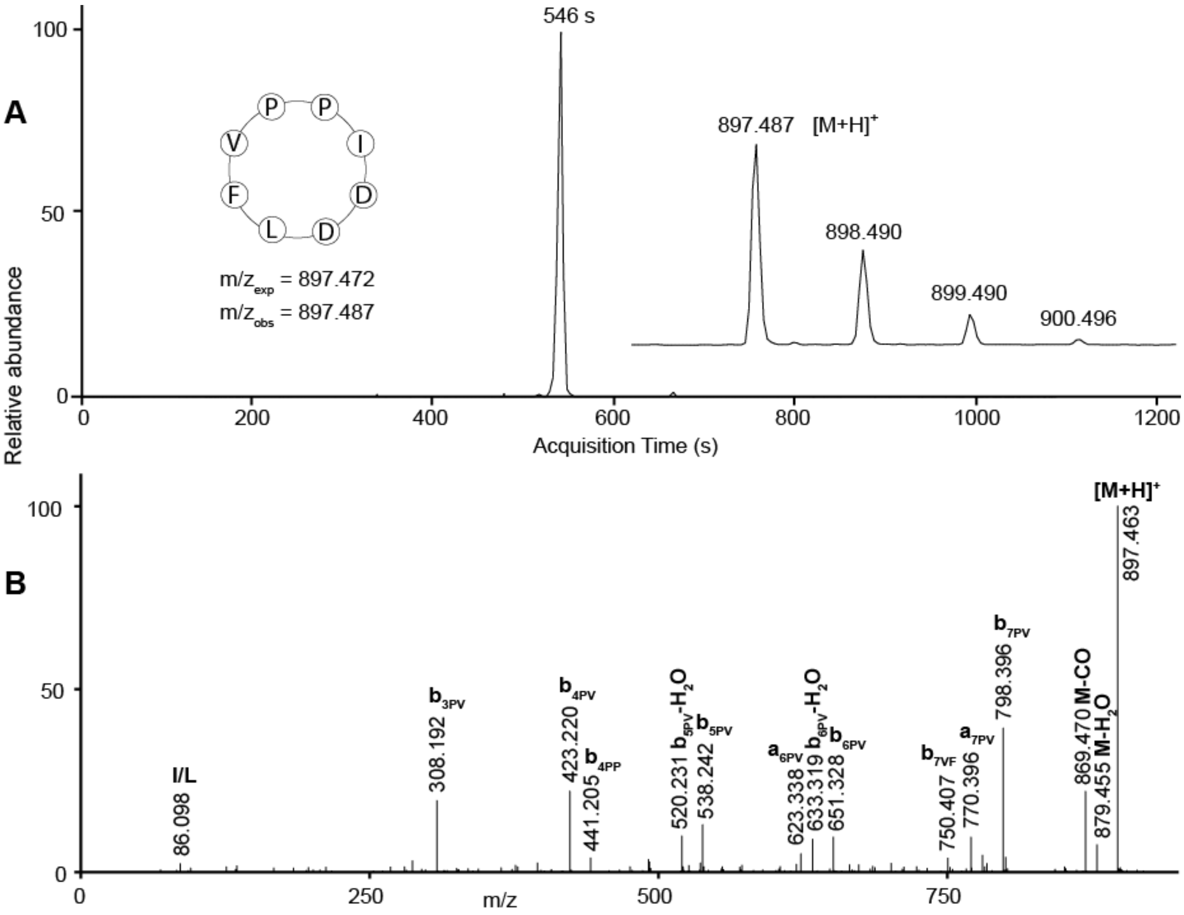

C

	D-D	L-D	F-L	V-F	P-V	P-P	I-P	D-I
D	116.035	897.472	784.388	637.320	***538.251***	***441.199***	344.146	231.062
L	229.119	114.092	897.472	***750.404***	***651.335***	554.283	457.230	344.146
F	376.187	261.160	148.076	897.472	***798.404***	701.351	604.298	491.214
V	475.256	360.229	247.145	100.076	***897.472***	800.419	703.367	590.283
P	572.308	457.281	344.197	197.129	98.061	897.472	800.419	687.335
P	669.361	554.334	441.250	294.182	195.113	98.061	897.472	784.388
I	782.445	667.418	554.334	407.266	***308.197***	211.145	114.092	897.472
D	897.472	782.445	669.361	522.293	***423.224***	326.172	229.119	116.035

Supplemental Figure 1. Q-TOF LC-MS data for PLP-2 in *Senecio pinnatifolius* ssp *latilobus*. A: Extracted ion chromatogram showing acquisition time of the peptide, with (inset left) peptide sequence with expected and observed mass-to-charge ratios (m/z) and (inset right) peptide mass spectrum. B: Tandem mass spectrum of the fragmented precursor ion. Immonium ions are denoted by the one-letter code of the residue they represent. C: Predicted b-ions following ring cleavage. Columns are for each cleavage point. Rows show the mass of the b-ion which contains the residue on the left at its C-terminus. Ions identified in the mass spectrum (either directly, or with loss of H_2O (subtract 18.011) or CO (subtract 27.995)) are shown in ***bold italics***.

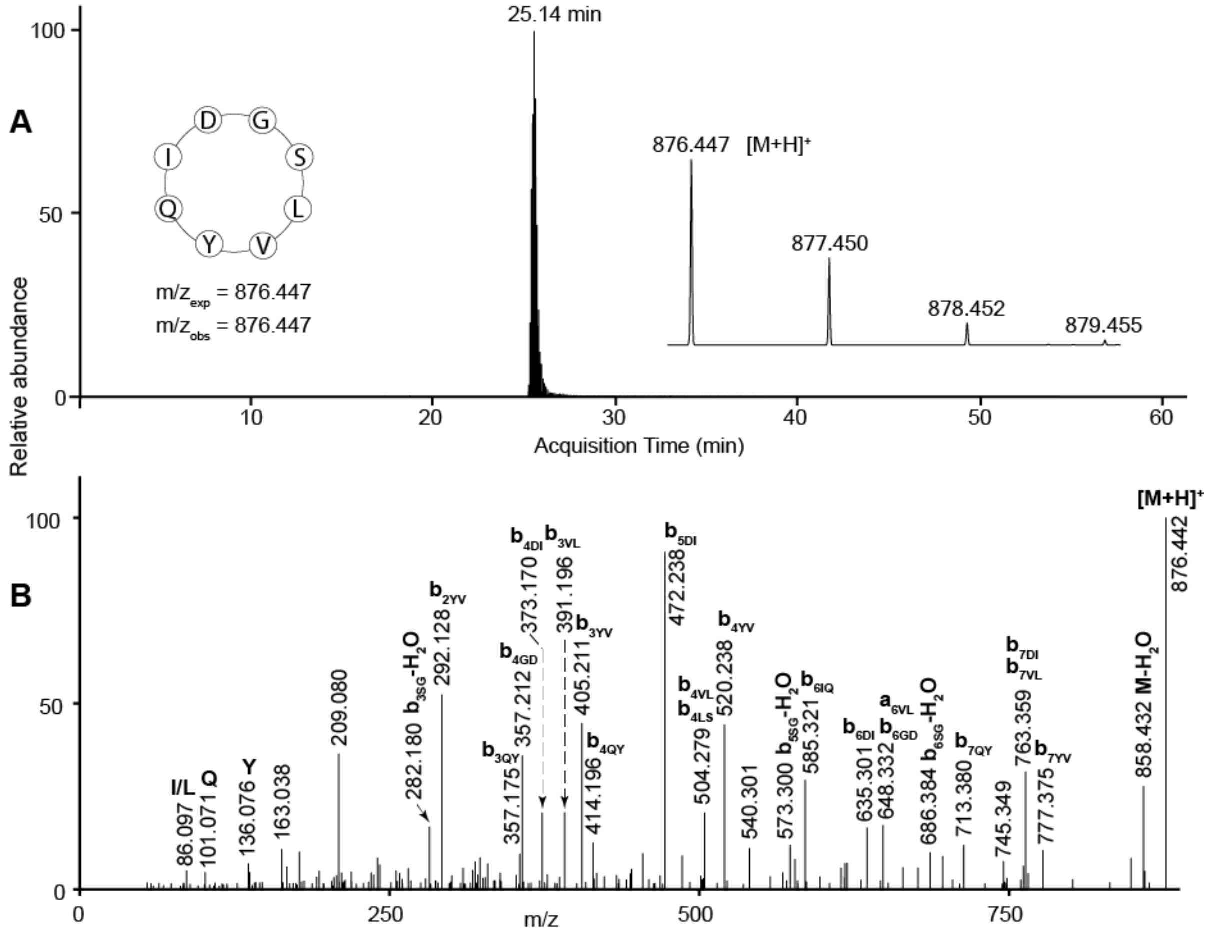

C	G-D	S-G	L-S	V-L	Y-V	Q-Y	I-Q	D-I
G	58.029	876.447	789.415	676.331	577.262	***414.199***	286.140	173.056
S	145.061	88.040	876.447	***763.363***	664.294	501.231	373.172	260.088
L	258.145	201.124	114.092	876.447	***777.378***	614.315	486.256	***373.172***
V	***357.214***	***300.192***	213.160	100.076	876.447	713.383	***585.325***	***472.241***
Y	520.277	463.256	376.224	263.140	164.071	876.447	748.388	***635.304***
Q	***648.336***	591.314	***504.282***	***391.198***	***292.130***	129.066	876.447	***763.363***
I	761.420	***704.398***	617.366	***504.282***	***405.214***	242.150	114.092	***876.447***
D	876.447	819.425	732.393	619.309	***520.241***	***357.177***	229.119	116.035

Supplemental Figure 2. Orbitrap LC-MS data for PLP-3 in *Senecio pinnatifolius* var *maritimus*. (**A**) Extracted ion chromatogram showing acquisition time of the peptide, with (inset left) peptide sequence with expected and observed mass-to-charge ratios (m/z) and (inset right) peptide mass spectrum. (**B**) Tandem mass spectrum of the fragmented precursor ion. Immonium ions are denoted by the one-letter code of the residue they represent. (**C**) Predicted b-ions following ring cleavage. Columns are for each cleavage point. Rows show the mass of the b-ion which contains the residue on the left at its C-terminus. Ions identified in the mass spectrum (either directly, or with loss of H_2O (subtract 18.011)) are shown in ***bold italics***.

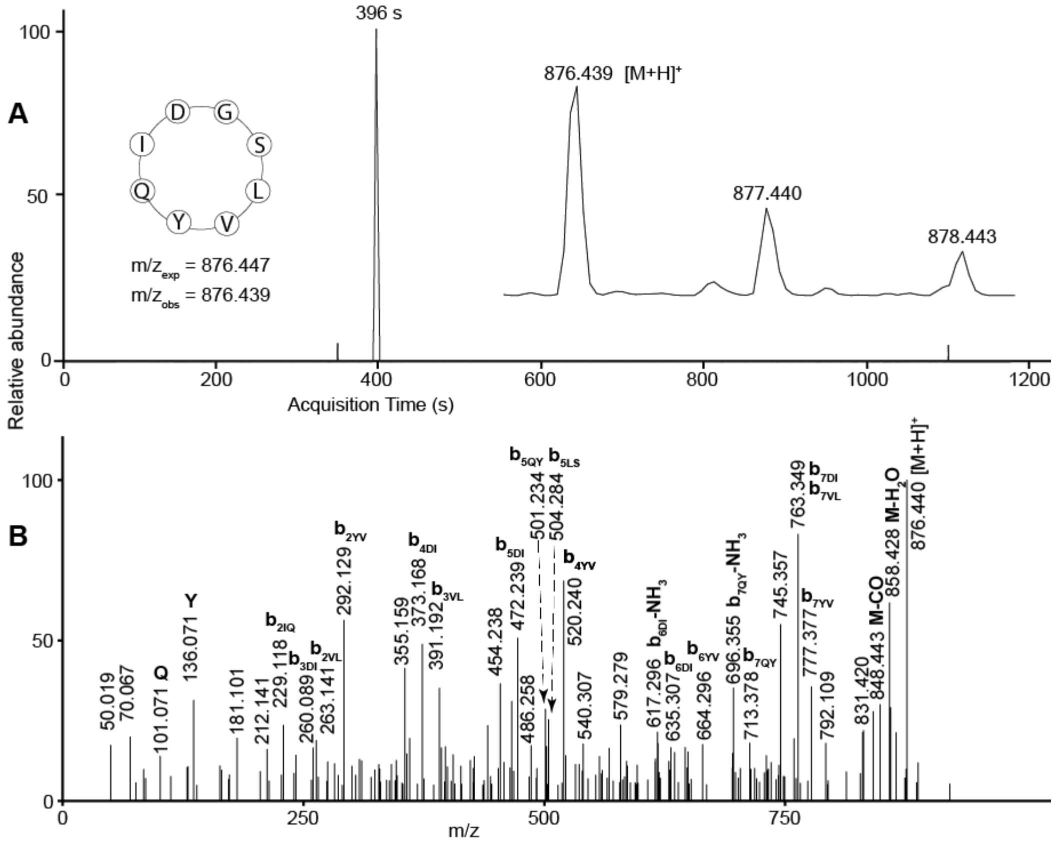

C	G-D	S-G	L-S	V-L	Y-V	Q-Y	I-Q	D-I
G	58.029	876.447	789.415	676.331	577.262	414.199	286.140	173.056
S	145.061	88.040	876.447	***763.363***	***664.294***	***501.231***	373.172	***260.088***
L	258.145	201.124	114.092	876.447	***777.378***	614.315	486.256	***373.172***
V	357.214	300.192	213.160	100.076	876.447	***713.383***	585.325	***472.241***
Y	520.277	463.256	376.224	***263.140***	164.071	876.447	748.388	***635.304***
Q	648.336	591.314	***504.282***	***391.198***	***292.130***	129.066	876.447	***763.363***
I	761.420	704.398	617.366	504.282	405.214	242.150	114.092	***876.447***
D	876.447	819.425	732.393	619.309	***520.241***	357.177	***229.119***	116.035

Supplemental Figure 3. Q-TOF LC-MS data for PLP-3 in *Senecio pinnatifolius* ssp *latilobus*. (**A**) Extracted ion chromatogram showing acquisition time of the peptide, peptide sequence with expected and observed mass-to-charge ratios (m/z) and peptide mass spectrum. (**B**) tandem mass spectrum of the fragmented precursor ion. Immonium ions are denoted by the one-letter code of the residue they represent. (**C**) Predicted b-ions following ring cleavage. Columns are for each cleavage point. Rows show the m/z of the b-ion which contains the residue on the left at its C-terminus. Ions identified in the mass spectrum (either directly, or with loss of H_2O (subtract 18.011), CO (subtract 27.995) or NH_3 (subtract 17.027)) are shown in ***bold italics***.

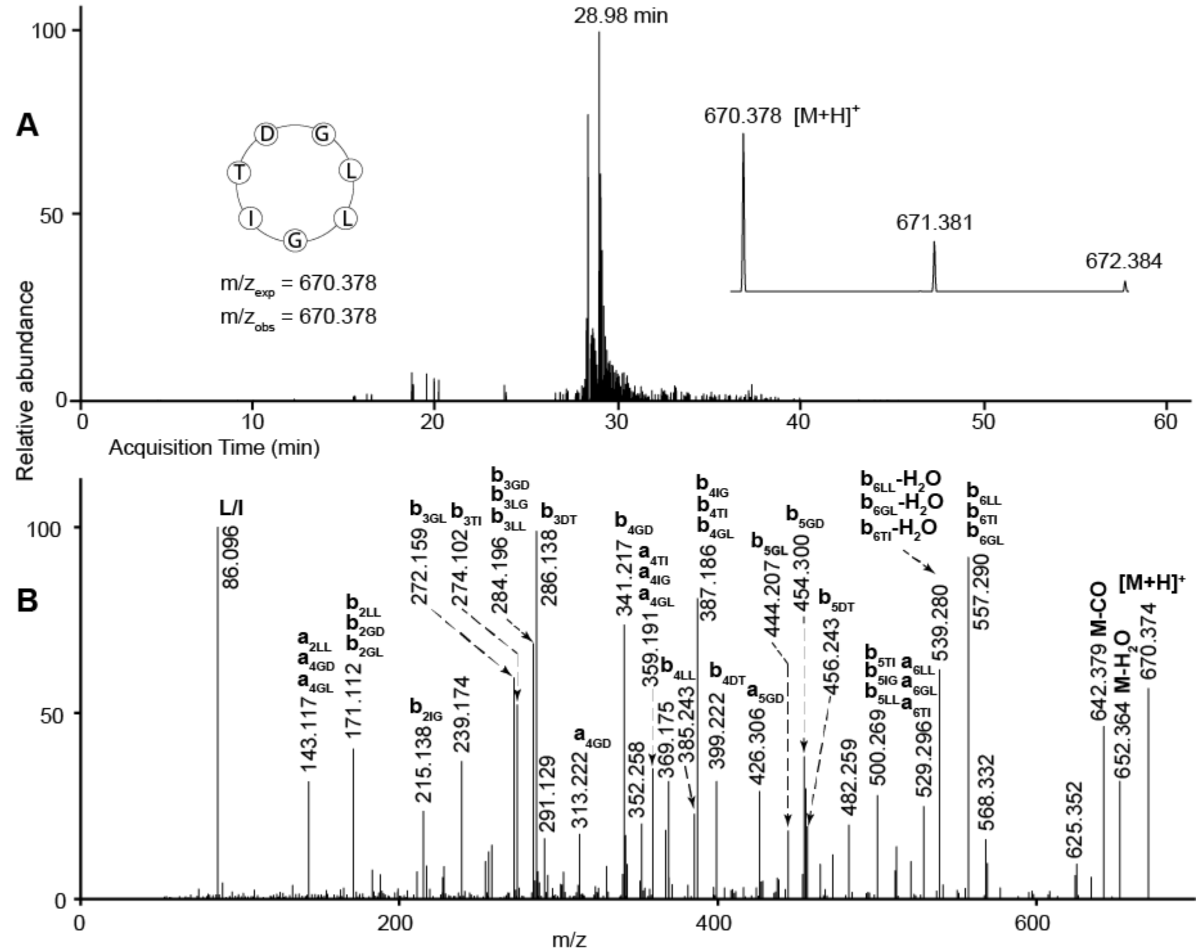

C	G-D	L-G	L-L	G-L	I-G	T-I	D-T
G	58.029	670.378	***557.293***	444.209	***387.188***	***274.104***	173.056
L	***171.113***	114.092	670. 378	***557.293***	***500.272***	***387.188***	***286.140***
L	***284.197***	227.176	114.092	670.378	613.356	***500.272***	***399.224***
G	***341.219***	***284.197***	***171.113***	58.029	670.378	***557.293***	***456.246***
I	***454.303***	397.281	***284.197***	***171.113***	114.092	670.378	569.330
T	555.351	498.329	***385.245***	***272.161***	***215.140***	102.056	670.378
D	670.378	613.356	***500.272***	***387.188***	330.167	217.082	116.035

Supplemental Figure 4. Orbitrap LC-MS data for PLP-4 in *Senecio pinnatifolius* var *maritimus*. (**A**) Extracted ion chromatogram showing acquisition time of the peptide, peptide sequence with expected and observed mass-to-charge ratios (m/z) and peptide mass spectrum. (**B**) Tandem mass spectrum of the fragmented precursor ion. Immonium ions are denoted by their one-letter residue code. (**C**) Predicted b-ions following ring cleavage. Columns are for each cleavage point. Rows show the mass of the b-ion which contains the residue on the left at its C-terminus. Ions identified in the mass spectrum (either directly, or with loss of H_2O or CO) are shown in ***bold italics***.

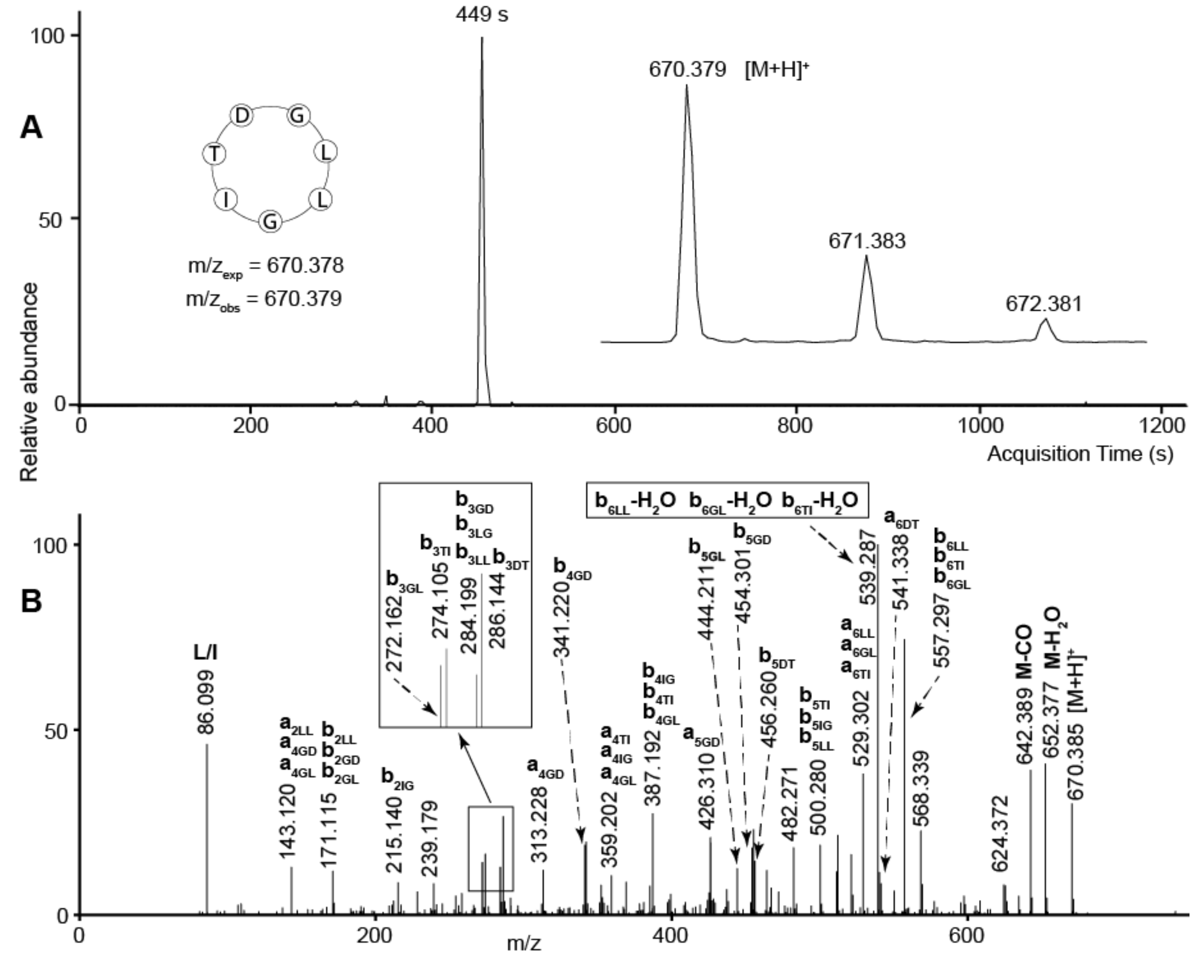

C	G-D	L-G	L-L	G-L	I-G	T-I	D-T
G	58.029	670.378	***557.293***	***444.209***	***387.188***	***274.104***	173.056
L	*171.113*	114.092	670.378	***557.293***	***500.272***	***387.188***	***286.140***
L	***284.197***	227.176	114.092	670.378	613.356	***500.272***	399.224
G	***341.219***	***284.197***	***171.113***	58.029	670.378	***557.293***	***456.246***
I	***454.303***	397.281	***284.197***	***171.113***	114.092	670.378	569.330
T	555.351	498.329	385.245	***272.161***	***215.140***	102.056	670.378
D	670.378	613.356	***500.272***	***387.188***	330.167	217.082	116.035

Supplemental Figure 5. Q-TOF LC-MS data for PLP-4 in *Senecio pinnatifolius* ssp *latilobus*. (**A**) Extracted ion chromatogram showing acquisition time of the peptide, peptide sequence with expected and observed mass-to-charge ratios (m/z) and peptide mass spectrum. (**B**) Tandem mass spectrum of the fragmented precursor ion. Immonium ions are denoted by their one-letter residue code. (**C**) Predicted b-ions following ring cleavage. Columns are for each cleavage point. Rows show the mass of the b-ion which contains the residue on the left at its C-terminus. Ions identified in the mass spectrum (either directly, or with loss of H_2O or CO) are shown in ***bold italics***.

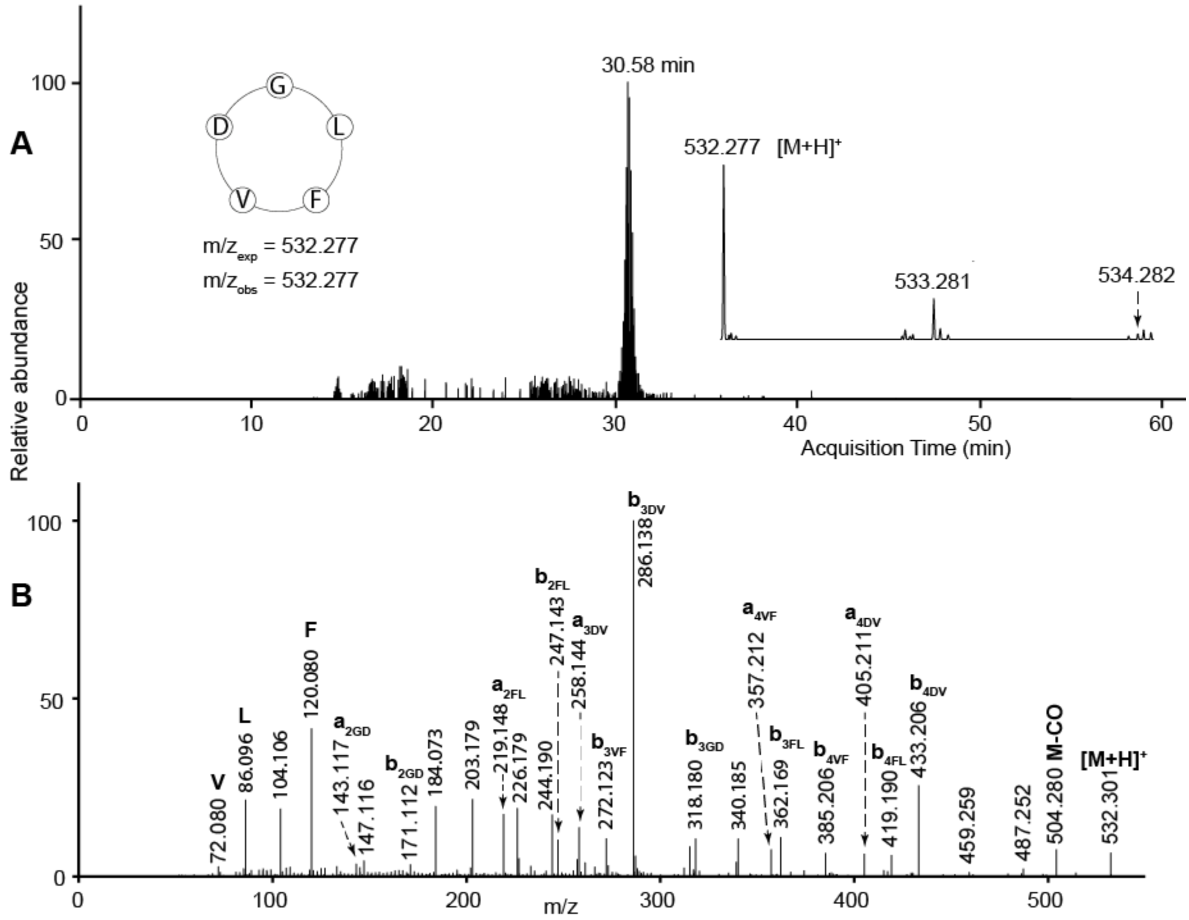

C

	G-D	L-G	F-L	V-F	D-V
G	58.029	532.277	***419.193***	***272.125***	173.056
L	***171.113***	114.092	532.277	***385.209***	286.140
F	***318.182***	261.160	148.076	532.277	***433.209***
V	417.250	360.229	***247.145***	100.076	532.277
D	532.277	475.256	***362.172***	215.103	116.035

Supplemental Figure 6. Orbitrap LC-MS data for PLP-5 in *Senecio pinnatifolius* var *maritimus*. (**A**) Extracted ion chromatogram showing acquisition time of the peptide, peptide sequence with expected and observed mass-to-charge ratios (m/z) and peptide mass spectrum. (**B**) Tandem mass spectrum of the fragmented precursor ion. Immonium ions are denoted by their one-letter residue code. (**C**) Predicted b-ions following ring cleavage. Columns are for each cleavage point. Rows show the mass of the b-ion which contains the residue on the left at its C-terminus. Ions identified in the mass spectrum (either directly, or with loss of H_2O or CO) are shown in ***bold italics***.

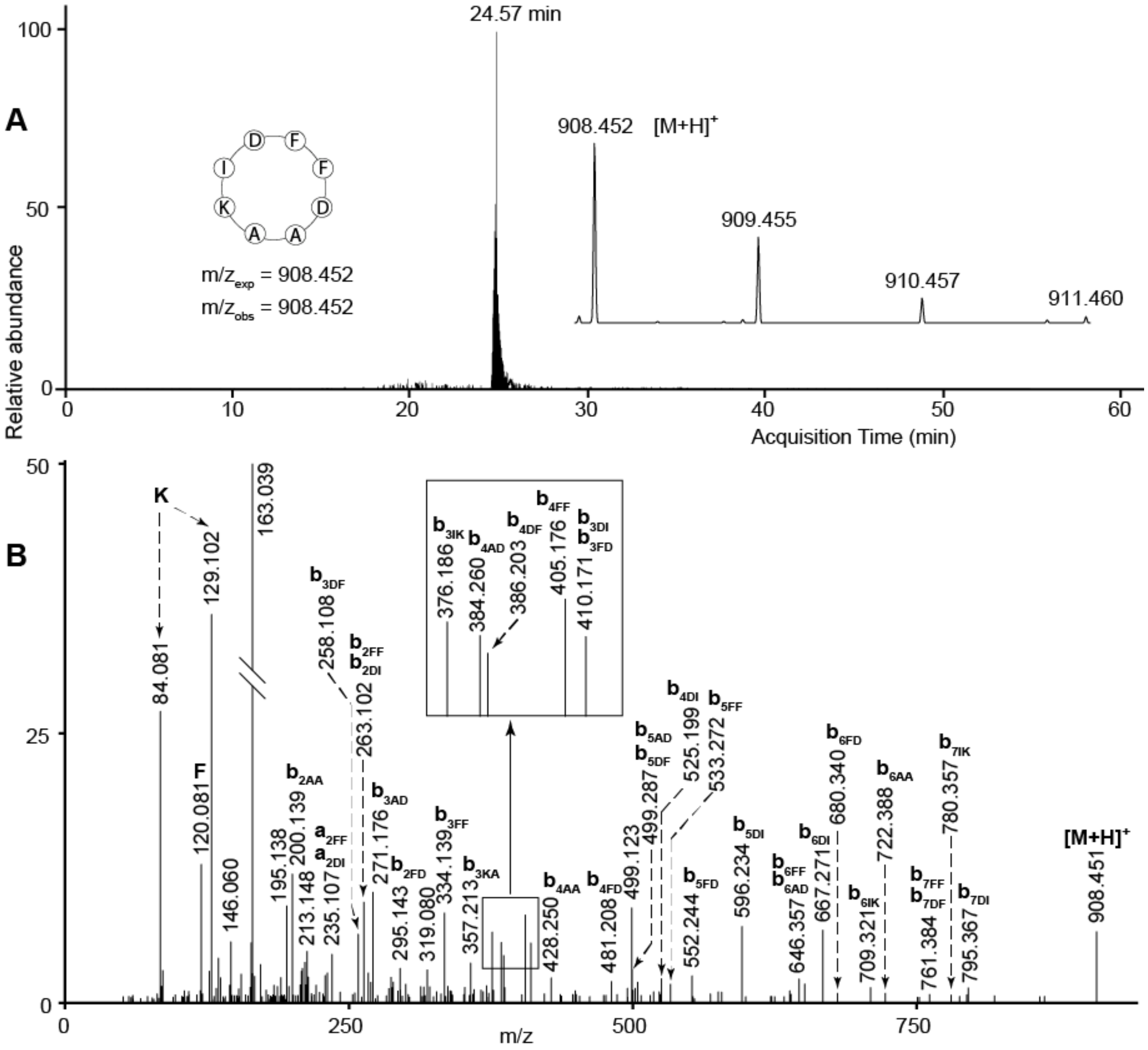

C

	F-D	F-F	D-F	A-D	A-A	K-A	I-K	D-I
F	148.076	908.452	***761.383***	***646.356***	575.319	504.282	***376.187***	***263.103***
F	***295.145***	148.076	908.452	793.425	***722.388***	651.351	523.256	***410.172***
D	***410.172***	***263.103***	116.035	908.452	837.415	766.378	638.283	***525.199***
A	***481.209***	***334.140***	187.072	72.045	908.452	837.415	***709.320***	***596.236***
A	***552.246***	***405.177***	***258.109***	143.082	72.045	908.452	***780.357***	***667.273***
K	***680.341***	***533.272***	***386.204***	***271.177***	***200.140***	129.103	908.452	***795.368***
I	793.425	***646.356***	***499.288***	***384.261***	313.224	242.187	114.092	908.452
D	908.452	***761.383***	614.315	***499.288***	428.251	***357.214***	229.119	116.035

Supplemental Figure 7. Orbitrap LC-MS data for PLP-6 in *Senecio pinnatifolius* var *maritimus*. (**A**) Extracted ion chromatogram showing acquisition time of the peptide, peptide sequence with expected and observed mass-to-charge ratios (m/z) and peptide mass spectrum. (**B**) Tandem mass spectrum of the fragmented precursor ion. Immonium ions are denoted by their one-letter residue code. (**C**) Predicted b-ions following ring cleavage. Columns are for each cleavage point. Rows show the mass of the b-ion which contains the residue on the left at its C-terminus. Ions identified in the mass spectrum (either directly, or with loss of H_2O or CO) are shown in ***bold italics***.

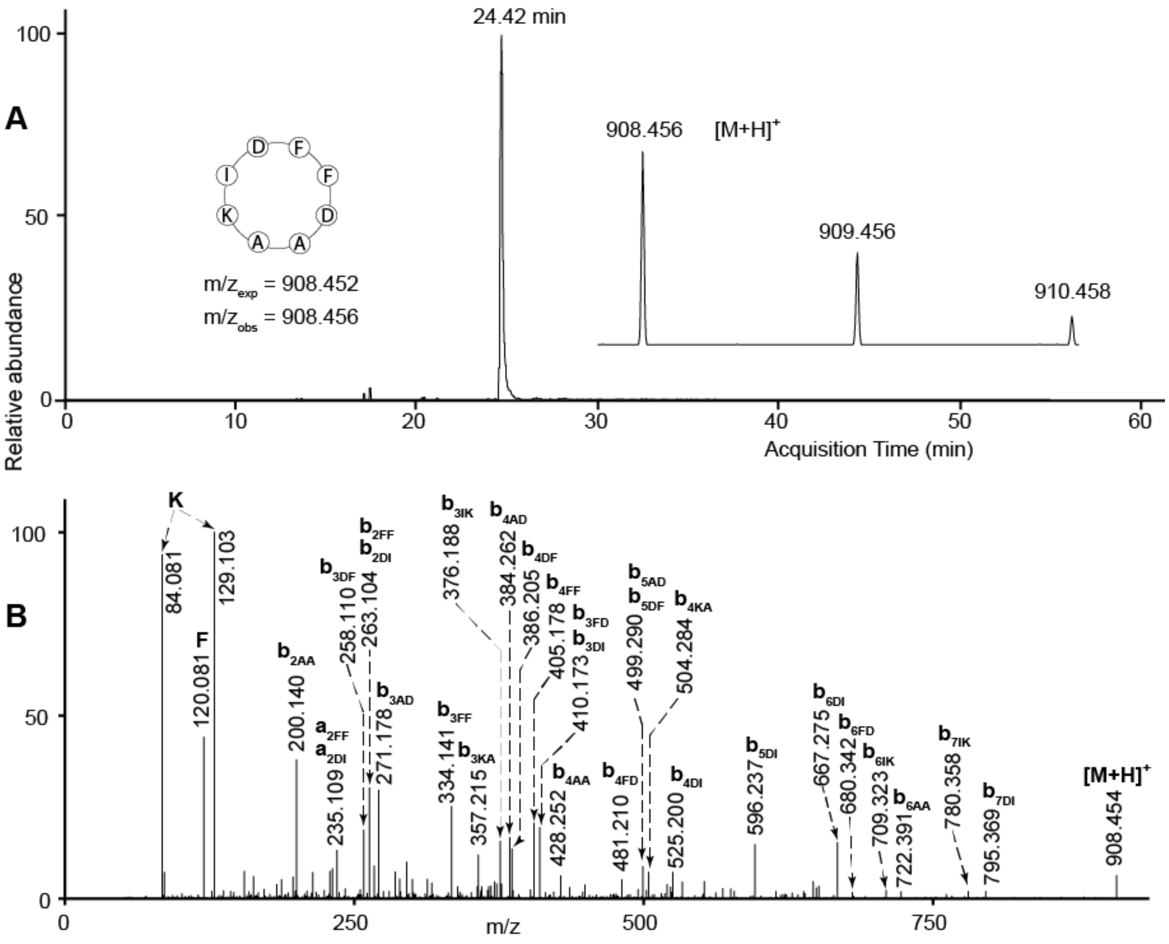

C	F-D	F-F	D-F	A-D	A-A	K-A	I-K	D-I
F	148.076	908.452	761.383	646.356	575.319	***504.282***	***376.187***	***263.103***
F	295.145	148.076	908.452	793.425	***722.388***	651.351	523.256	***410.172***
D	***410.172***	***263.103***	116.035	908.452	837.415	766.378	638.283	***525.199***
A	***481.209***	***334.140***	187.072	72.045	908.452	837.415	***709.320***	***596.236***
A	552.246	***405.177***	***258.109***	143.082	72.045	908.452	***780.357***	***667.273***
K	***680.341***	533.272	***386.204***	***271.177***	***200.140***	129.103	908.452	***795.368***
I	793.425	646.356	***499.288***	***384.261***	313.224	242.187	114.092	908.452
D	908.452	761.383	614.315	***499.288***	***428.251***	***357.214***	229.119	116.035

Supplemental Figure 8. Orbitrap LC-MS data for PLP-6 in *Senecio pinnatifolius* ssp *latilobus*. (**A**) Extracted ion chromatogram showing acquisition time of the peptide, peptide sequence with expected and observed mass-to-charge ratios (m/z) and peptide mass spectrum. (**B**) Tandem mass spectrum of the fragmented precursor ion. Immonium ions are denoted by their one-letter residue code. (**C**) Predicted b-ions following ring cleavage. Columns are for each cleavage point. Rows show the mass of the b-ion which contains the residue on the left at its C-terminus. Ions identified in the mass spectrum (either directly, or with loss of H_2O or CO) are shown in ***bold italics***.

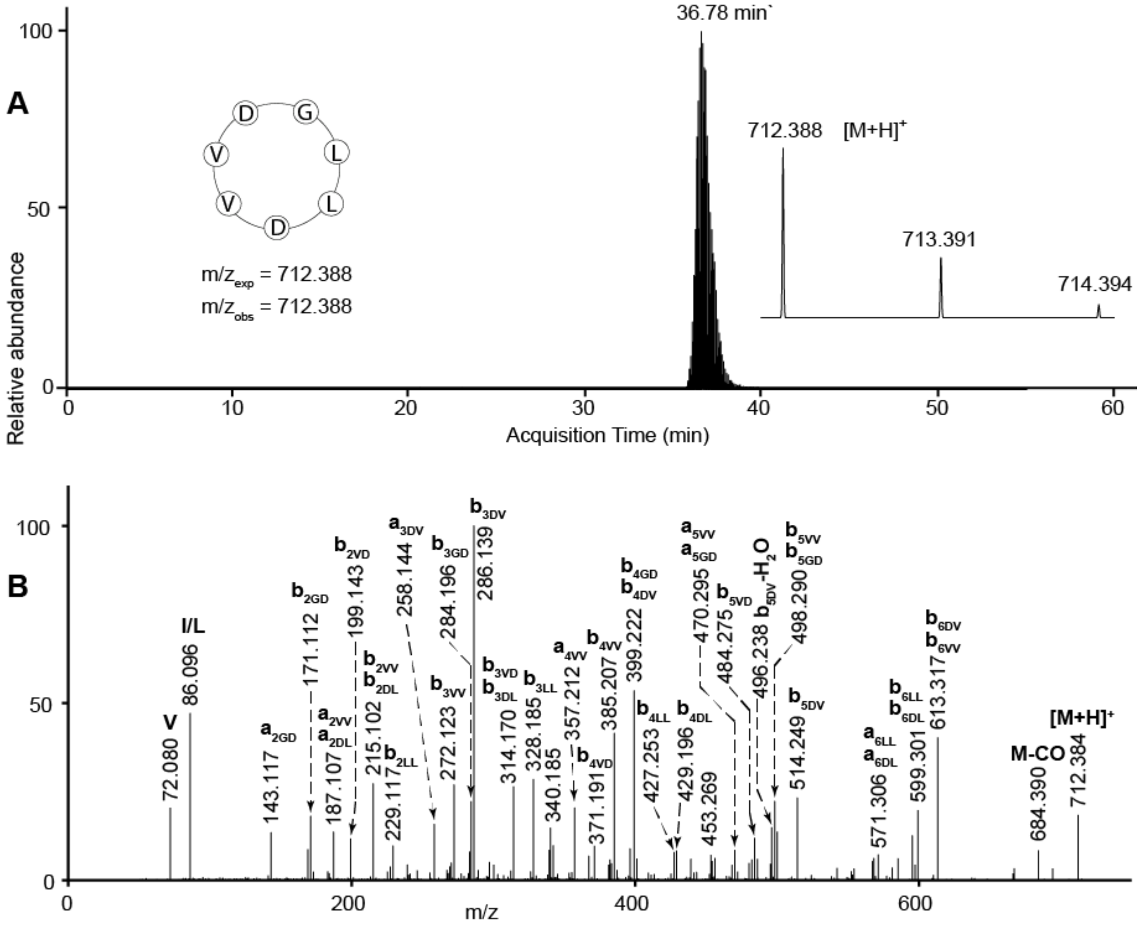

C	G-D	L-G	L-L	D-L	V-D	V-V	D-V
G	58.029	712.388	***599.304***	486.220	***371.193***	***272.125***	173.056
L	***171.113***	114.092	712.388	***599.304***	***484.277***	***385.209***	***286.140***
L	***284.197***	227.176	114.092	712.388	597.361	***498.293***	***399.224***
D	***399.224***	342.203	***229.119***	116.035	712.388	***613.320***	***514.251***
V	***498.293***	441.271	***328.187***	***215.103***	100.076	712.388	***613.320***
V	597.361	540.340	***427.256***	***314.172***	***199.145***	100.076	712.388
D	712.388	655.367	542.283	***429.199***	***314.172***	***215.103***	116.035

Supplemental Figure 9. Orbitrap LC-MS data for PLP-7 in *Senecio pinnatifolius* var *maritimus*. (**A**) Extracted ion chromatogram showing acquisition time of the peptide, peptide sequence with expected and observed mass-to-charge ratios (m/z) and peptide mass spectrum. (**B**) Tandem mass spectrum of the fragmented precursor ion. Immonium ions are denoted by their one-letter residue code. (**C**) Predicted b-ions following ring cleavage. Columns are for each cleavage point. Rows show the mass of the b-ion which contains the residue on the left at its C-terminus. Ions identified in the mass spectrum (either directly, or with loss of H_2O or CO) are shown in ***bold italics***.

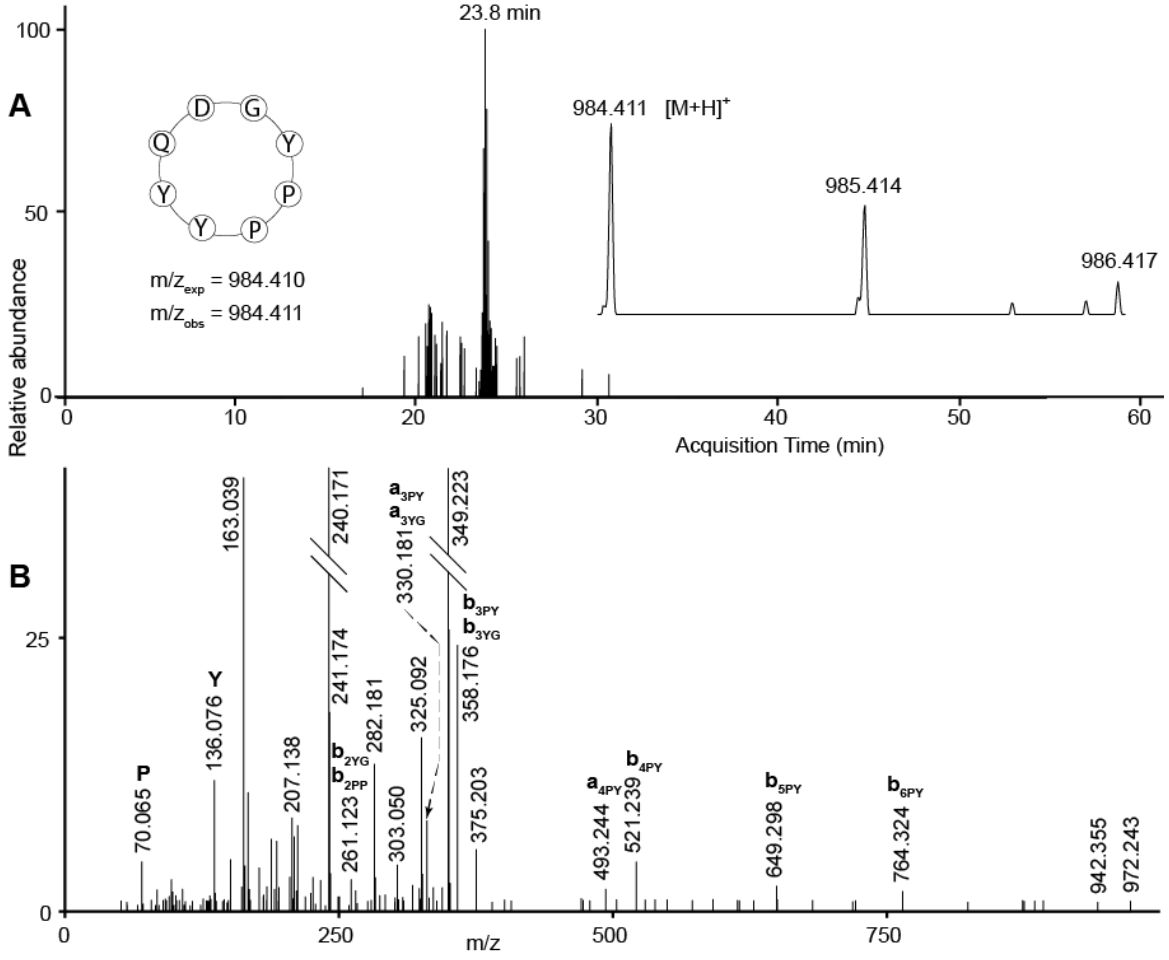

C

	G-D	Y-G	P-Y	P-P	Y-P	Y-Y	Q-Y	D-Q
G	58.029	984.410	821.347	724.294	627.241	464.178	301.115	173.056
Y	221.093	164.071	984.410	887.358	790.305	627.241	464.178	336.120
P	318.145	***261.124***	98.061	984.410	887.358	724.294	561.231	433.172
P	415.198	***358.177***	195.113	98.061	984.410	821.347	658.284	530.225
Y	578.261	521.240	***358.177***	***261.124***	164.071	984.410	821.347	693.288
Y	741.325	684.303	***521.240***	424.187	327.134	164.071	984.410	856.352
Q	869.383	812.362	***649.299***	552.246	455.193	292.130	129.066	984.410
D	984.410	927.389	***764.326***	667.273	570.220	407.157	244.093	116.035

Supplemental Figure 10. Orbitrap LC-MS data for PLP-8 in *Zinnia haageana*. (**A**) Extracted ion chromatogram showing acquisition time of the peptide, peptide sequence with expected and observed mass-to-charge ratios (m/z) and peptide mass spectrum. (**B**) Tandem mass spectrum of the fragmented precursor ion. Immonium ions are denoted by their one-letter residue code. (**C**) Predicted b-ions following ring cleavage. Columns are for each cleavage point. Rows show the mass of the b-ion which contains the residue on the left at its C-terminus. Ions identified in the mass spectrum (either directly or with loss of H_2O) are shown in ***bold italics***.

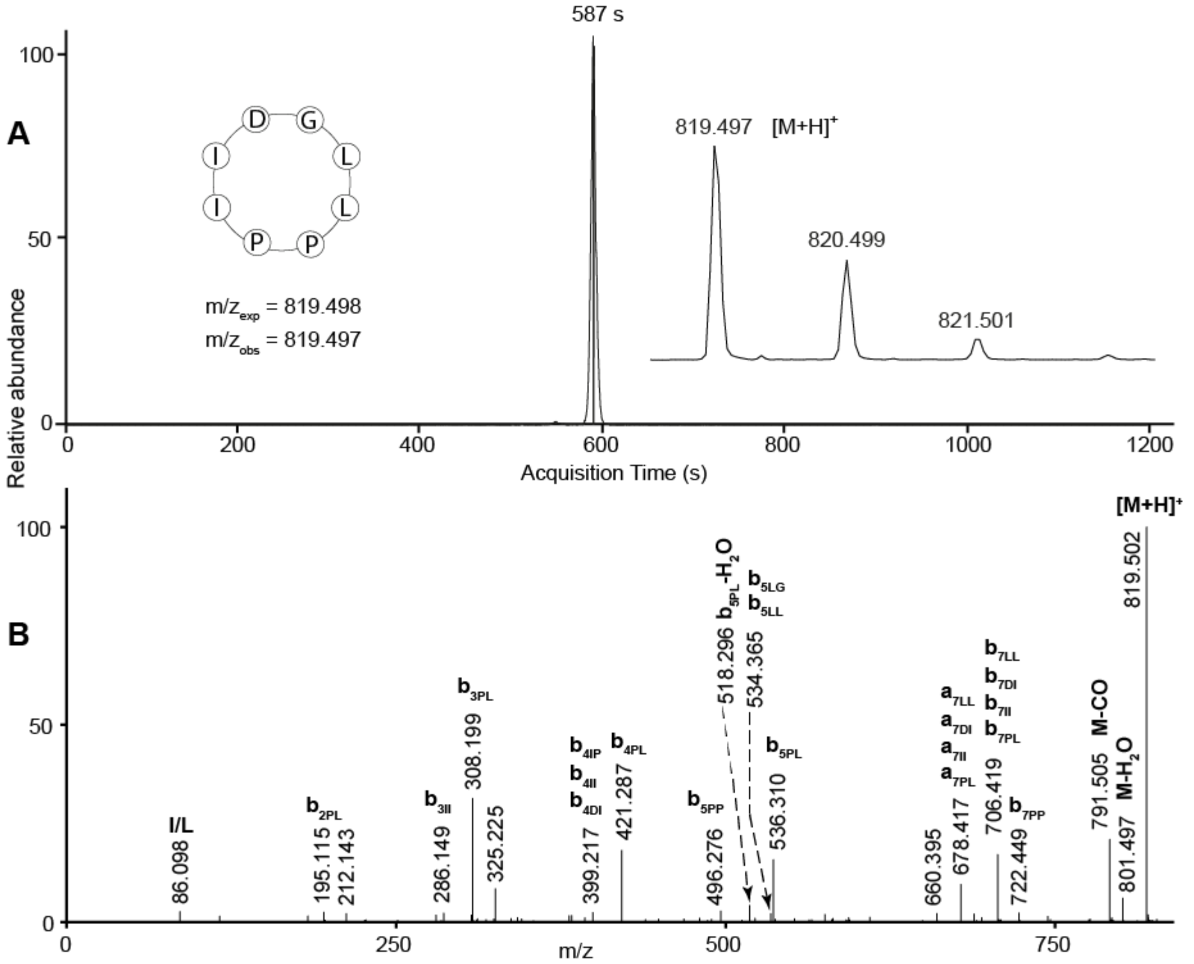

C

	G-D	L-G	L-L	P-L	P-P	I-P	I-I	D-I
G	58.029	819.498	***706.414***	593.330	***496.277***	***399.224***	***286.140***	173.056
L	171.113	114.092	819.498	***706.414***	609.361	512.308	***399.224***	286.140
L	284.197	227.176	114.092	819.498	***722.445***	625.392	512.308	***399.224***
P	381.250	324.229	211.145	98.061	819.498	722.445	609.361	496.277
P	478.303	421.281	308.197	***195.113***	98.061	819.498	***706.414***	593.330
I	591.387	***534.366***	421.281	***308.197***	211.145	114.092	819.498	***706.414***
I	704.471	647.450	***534.366***	***421.281***	324.229	227.176	114.092	819.498
D	819.498	762.477	649.392	***536.308***	439.256	342.203	229.119	116.035

Supplemental Figure 11. Q-TOF LC-MS data for PLP-9 in *Zinnia elegans*. (**A**) Extracted ion chromatogram showing acquisition time of the peptide, peptide sequence with expected and observed mass-to-charge ratios (m/z) and peptide mass spectrum. (**B**) Tandem mass spectrum of the fragmented precursor ion. Immonium ions are denoted by their one-letter residue code. (**C**) Predicted b-ions following ring cleavage. Columns are for each cleavage point. Rows show the mass of the b-ion which contains the residue on the left at its C-terminus. Ions identified in the mass spectrum (either directly, or with loss of H_2O or CO) are shown in ***bold italics***.

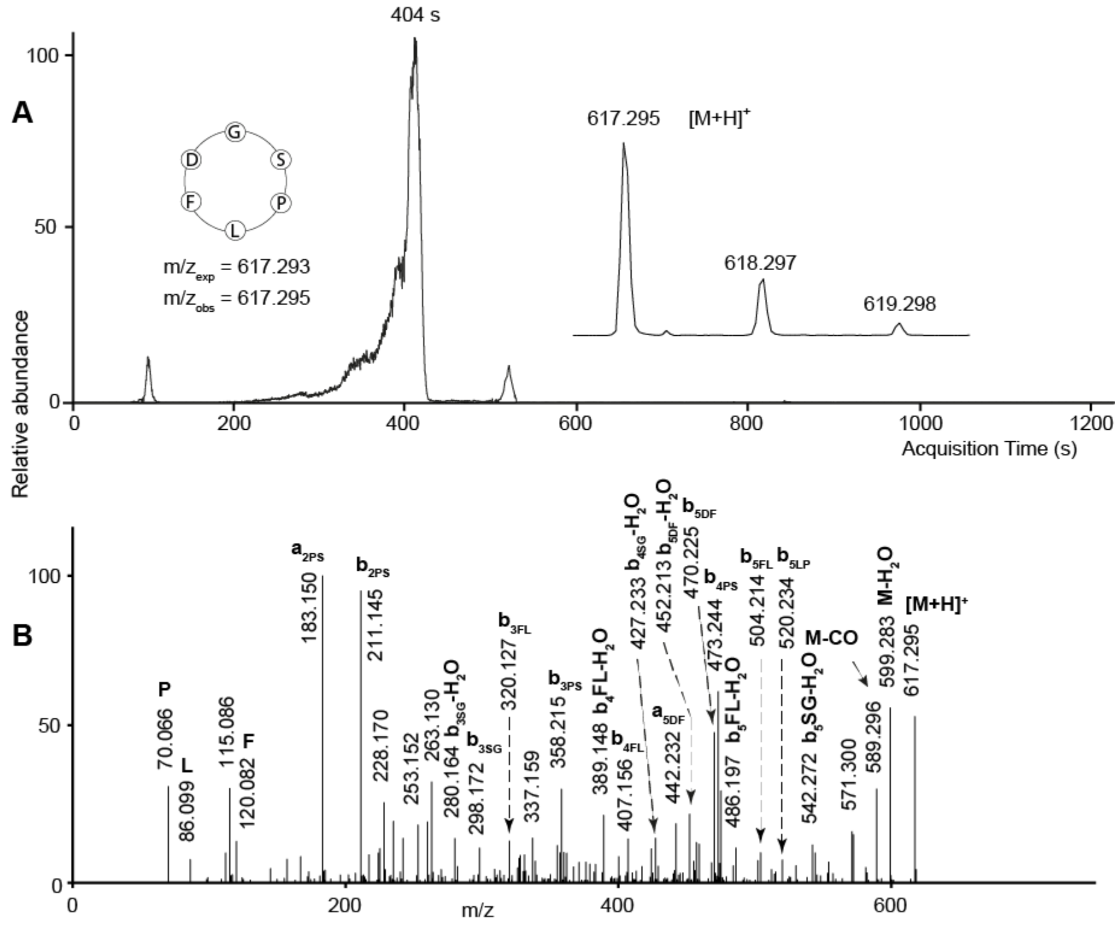

C

	G - D	S - G	P - S	L - P	F - L	D - F
G	58.029	617.293	530.261	433.209	***320.125***	173.056
S	145.061	88.040	617.293	***520.241***	***407.157***	260.088
P	242.114	185.093	98.061	617.293	***504.209***	357.141
L	355.198	***298.177***	***211.145***	114.092	617.293	***470.225***
F	502.267	***445.245***	***358.213***	261.160	148.076	617.293
D	617.293	***560.272***	***473.240***	376.187	263.103	116.035

Supplemental Figure 12. Q-TOF LC-MS data for PLP-10 in *Zinnia elegans*. (**A**) Extracted ion chromatogram showing acquisition time of the peptide, peptide sequence with expected and observed mass-to-charge ratios (m/z) and peptide mass spectrum. (**B**) Tandem mass spectrum of the fragmented precursor ion. Immonium ions are denoted by their one-letter residue code. (**C**) Predicted b-ions following ring cleavage. Columns are for each cleavage point. Rows show the mass of the b-ion which contains the residue on the left at its C-terminus. Ions identified in the mass spectrum (either directly, or with loss of H_2O or CO) are shown in ***bold italics***.

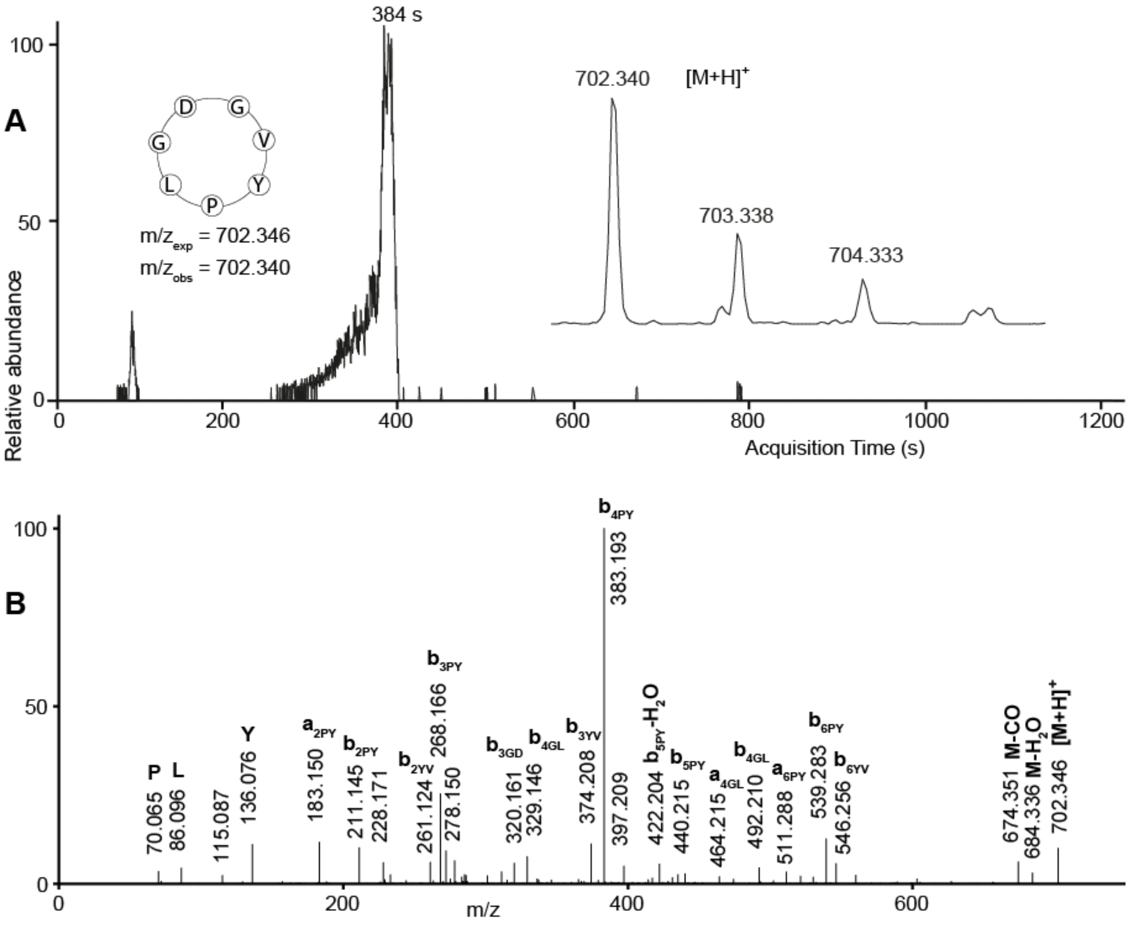

C

	G-D	V-G	Y-V	P-Y	L-P	G-L	D-G
G	58.029	702.346	603.278	***440.215***	343.162	230.078	173.056
V	157.098	100.076	702.346	***539.283***	442.230	***329.146***	272.125
Y	***320.161***	263.140	164.071	702.346	605.293	***492.209***	435.188
P	417.214	360.192	***261.124***	98.061	702.346	589.262	532.241
L	530.298	473.276	***374.208***	***211.145***	114.092	702.346	645.325
G	587.319	530.298	431.229	***268.166***	171.113	58.029	702.346
D	702.346	645.325	***546.256***	***383.193***	286.140	173.056	116.035

Supplemental Figure 13. Q-TOF LC-MS data for PLP-11 in *Zinnia elegans*. (**A**) Extracted ion chromatogram showing acquisition time of the peptide, peptide sequence with expected and observed mass-to-charge ratios (m/z) and peptide mass spectrum. (**B**) Tandem mass spectrum of the fragmented precursor ion. Immonium ions are denoted by their one-letter residue code. (**C**) Predicted b-ions following ring cleavage. Columns are for each cleavage point. Rows show the mass of the b-ion which contains the residue on the left at its C-terminus. Ions identified in the mass spectrum (either directly or with loss of H_2O or CO) are shown in ***bold italics***.

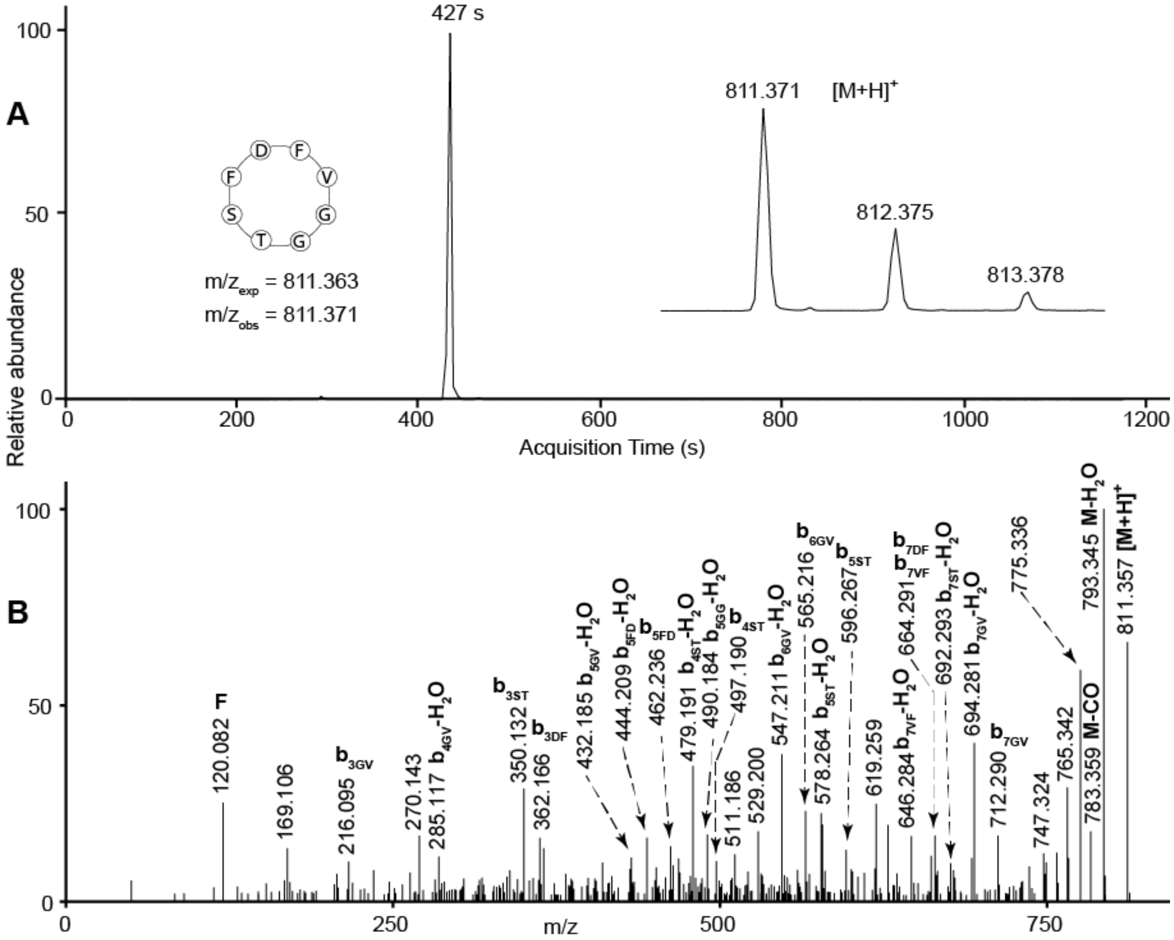

C

	F-D	V-F	G-V	G-G	T-G	S-T	F-S	D-F
F	148.076	811.363	***712.294***	655.273	598.251	***497.204***	410.172	263.103
V	247.145	100.076	811.363	754.341	697.320	***596.272***	509.240	***362.172***
G	304.166	157.098	58.029	811.363	754.341	653.293	566.261	419.193
G	361.188	214.119	115.051	58.029	811.363	***710.315***	623.283	476.215
T	***462.235***	315.167	***216.098***	159.077	102.056	811.363	724.331	577.262
S	549.267	402.199	***303.130***	246.109	189.088	88.040	811.363	***664.294***
F	696.336	549.267	***450.199***	393.177	336.156	235.108	148.076	811.363
D	811.363	***664.294***	***565.226***	***508.204***	451.183	***350.135***	263.103	116.035

Supplemental Figure 14. Q-TOF LC-MS data for PLP-12 in *Senecio vulgaris*. (**A**) Extracted ion chromatogram showing acquisition time of the peptide, peptide sequence with expected and observed mass-to-charge ratios (m/z) and peptide mass spectrum. (**B**) Tandem mass spectrum of the fragmented precursor ion. Immonium ions are denoted by their one-letter residue code. (**C**) Predicted b-ions following ring cleavage. Columns are for each cleavage point. Rows show the mass of the b-ion which contains the residue on the left at its C-terminus. Ions identified in the mass spectrum (either directly or with loss of H_2O or CO) are shown in ***bold italics***.

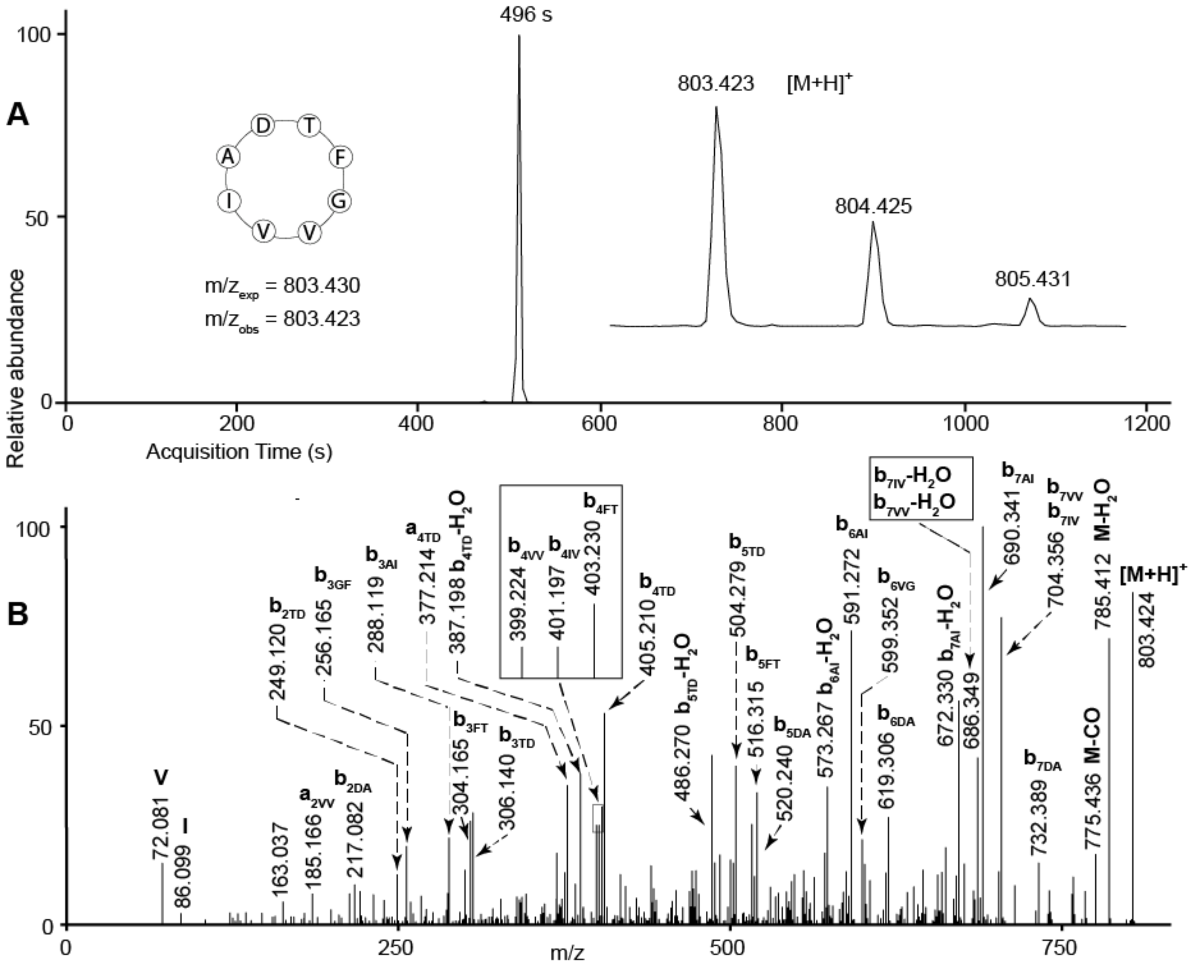

C

	T-D	F-T	G-F	V-G	V-V	I-V	A-I	D-A
T	102.056	803.430	656.362	***599.340***	500.272	***401.204***	***288.120***	***217.082***
F	***249.124***	148.076	803.430	746.409	647.340	548.272	435.188	364.151
G	***306.145***	205.098	58.029	803.430	***704.362***	605.293	492.209	421.172
V	***405.214***	***304.166***	157.098	100.076	803.430	***704.362***	***591.278***	***520.241***
V	***504.282***	***403.235***	***256.166***	199.145	100.076	803.430	***690.346***	***619.309***
I	617.366	***516.319***	369.250	312.229	213.160	114.092	803.430	732.393
A	688.403	587.356	440.287	383.266	284.197	185.129	72.045	803.430
D	803.430	702.383	555.314	498.293	***399.224***	300.156	187.072	116.035

Supplemental Figure 15. Q-TOF LC-MS data for PLP-13 in *Senecio vulgaris*. (**A**) Extracted ion chromatogram showing acquisition time of the peptide, peptide sequence with expected and observed mass-to-charge ratios (m/z) and peptide mass spectrum. (**B**) Tandem mass spectrum of the fragmented precursor ion. Immonium ions are denoted by their one-letter residue code. (**C**) Predicted b-ions following ring cleavage. Columns are for each cleavage point. Rows show the mass of the b-ion which contains the residue on the left at its C-terminus. Ions identified in the mass spectrum (either directly, or with loss of H_2O or CO) are shown in ***bold italics***.

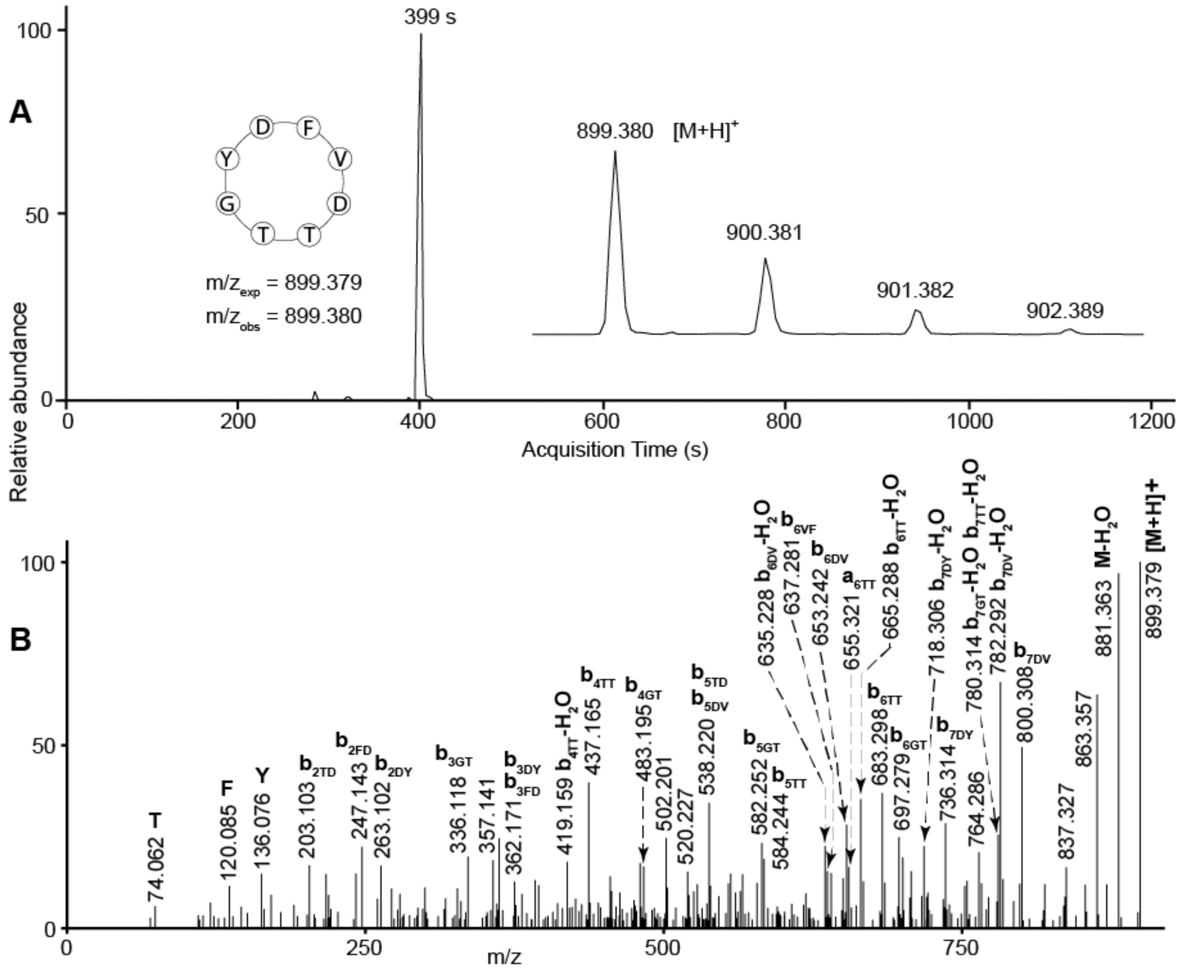

C	F-D	V-F	D-V	T-D	T-T	G-T	Y-G	D-Y
F	148.076	899.379	***800.310***	685.283	***584.236***	***483.188***	426.167	***263.103***
V	***247.145***	100.076	899.379	784.352	***683.304***	***582.256***	525.235	***362.172***
D	***362.172***	215.103	116.035	899.379	***798.331***	***697.283***	640.262	477.199
T	463.219	316.151	217.082	102.056	899.379	***798.331***	741.310	578.246
T	564.267	417.199	318.130	***203.103***	102.056	899.379	842.357	679.294
G	621.288	474.220	375.152	260.125	159.077	58.029	899.379	***736.315***
Y	784.352	***637.283***	***538.215***	423.188	322.140	221.093	164.071	899.379
D	899.379	752.310	***653.242***	***538.215***	***437.167***	***336.120***	279.098	116.035

Supplemental Figure 16. Q-TOF LC-MS data for PLP-14 in *Senecio vulgaris*. (**A**) Extracted ion chromatogram showing acquisition time of the peptide, peptide sequence with expected and observed mass-to-charge ratios (m/z) and peptide mass spectrum. (**B**) Tandem mass spectrum of the fragmented precursor ion. Immonium ions are denoted by their one-letter residue code. (**C**) Predicted b-ions following ring cleavage. Columns are for each cleavage point. Rows show the mass of the b-ion which contains the residue on the left at its C-terminus. Ions identified in the mass spectrum (either directly or with loss of H_2O or CO) are shown in ***bold italics***.

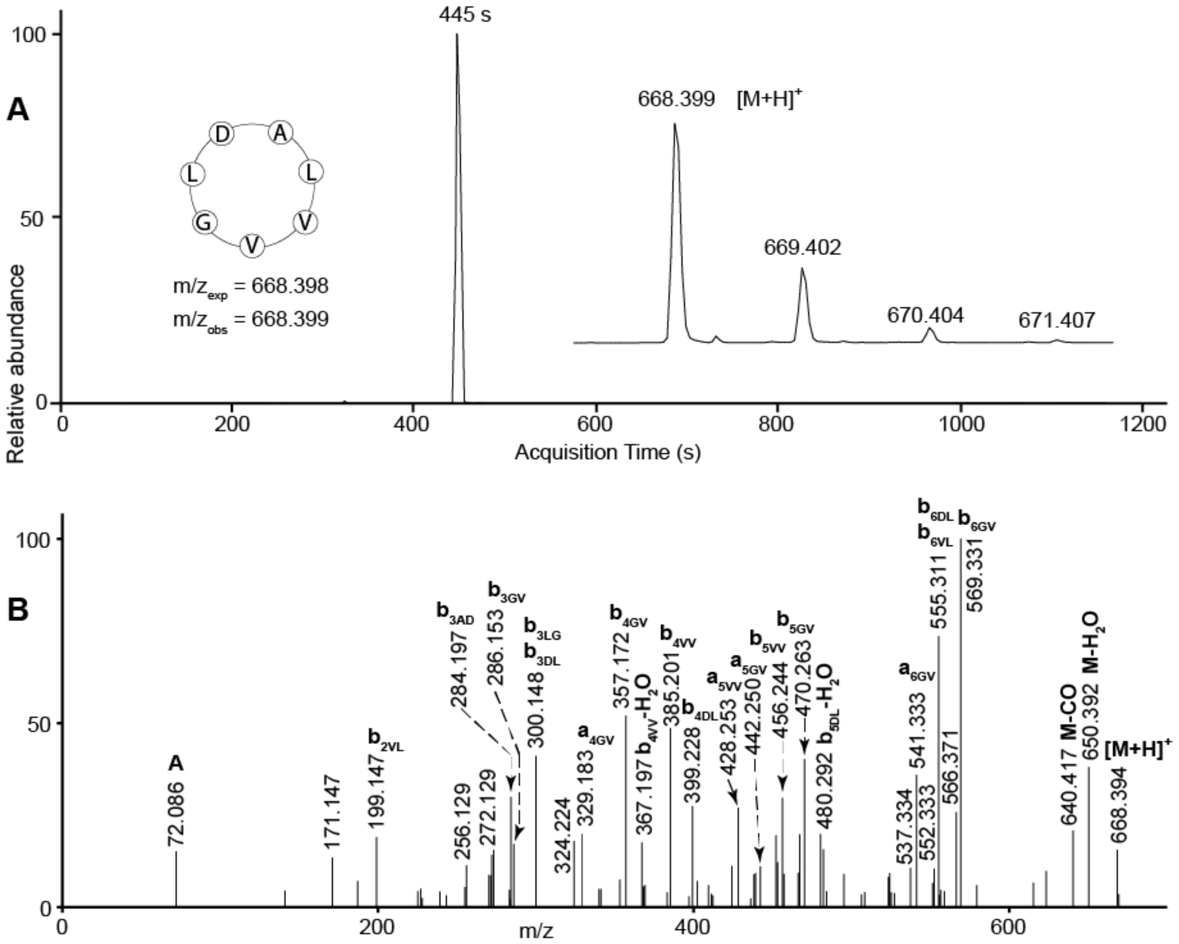

C	A-D	L-A	V-L	V-V	G-V	L-G	D-L
A	72.045	668.398	***555.314***	***456.246***	***357.177***	***300.156***	187.072
L	185.129	114.092	668.398	569.330	***470.261***	413.240	***300.156***
V	***284.197***	213.160	100.076	668.398	***569.330***	512.308	***399.224***
V	383.266	312.229	***199.145***	100.076	668.398	611.377	***498.293***
G	440.287	369.250	256.166	157.098	58.029	668.398	***555.314***
L	553.371	482.334	369.250	270.182	171.113	114.092	668.398
D	668.398	597.361	484.277	***385.209***	***286.140***	229.119	116.035

Supplemental Figure 17. Q-TOF LC-MS data for PLP-15 in *Senecio vulgaris*. (**A**) Extracted ion chromatogram showing acquisition time of the peptide, peptide sequence with expected and observed mass-to-charge ratios (m/z) and peptide mass spectrum. (**B**) Tandem mass spectrum of the fragmented precursor ion. Immonium ions are denoted by their one-letter residue code. (**C**) Predicted b-ions following ring cleavage. Columns are for each cleavage point. Rows show the mass of the b-ion which contains the residue on the left at its C-terminus. Ions identified in the mass spectrum (either directly, or with loss of H_2O or CO) are shown in ***bold italics***.

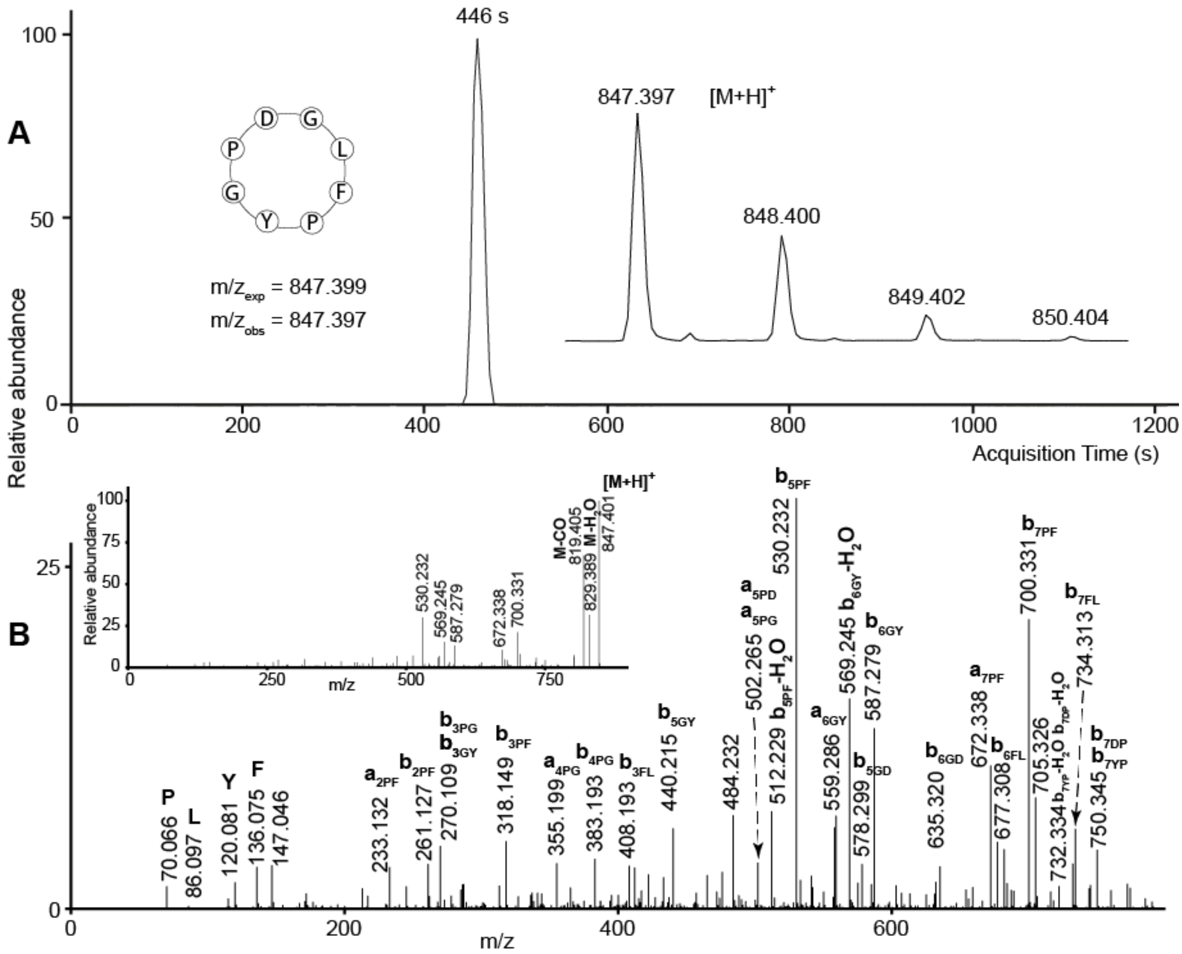

C

	G-D	L-G	F-L	P-F	Y-P	G-Y	P-G	D-P
G	58.029	847.399	***734.315***	587.247	490.194	327.130	***270.109***	173.056
L	171.113	114.092	847.399	***700.331***	603.278	***440.215***	***383.193***	286.140
F	318.182	261.160	148.076	847.399	***750.346***	***587.283***	530.261	433.209
P	415.235	358.213	245.129	98.061	847.399	684.336	627.314	530.261
Y	***578.298***	521.276	***408.192***	***261.124***	164.071	847.399	790.378	693.325
G	***635.319***	578.298	465.214	***318.145***	221.093	58.029	847.399	***750.346***
P	732.372	675.351	562.267	415.198	318.145	155.082	98.061	847.399
D	847.399	790.378	***677.293***	***530.225***	433.172	***270.109***	213.088	116.035

Supplemental Figure 18. Q-TOF LC-MS data for PLP-16 in *Dahlia variabilis*. (**A**) Extracted ion chromatogram showing acquisition time of the peptide, peptide sequence with expected and observed mass-to-charge ratios (m/z) and peptide mass spectrum. (**B**) Tandem mass spectrum of the fragmented precursor ion. Immonium ions are denoted by their one-letter residue code. (**C**) Predicted b-ions following ring cleavage. Columns are for each cleavage point. Rows show the mass of the b-ion which contains the residue on the left at its C-terminus. Ions identified in the mass spectrum (either directly, or with loss of H_2O or CO) are shown in ***bold italics***.

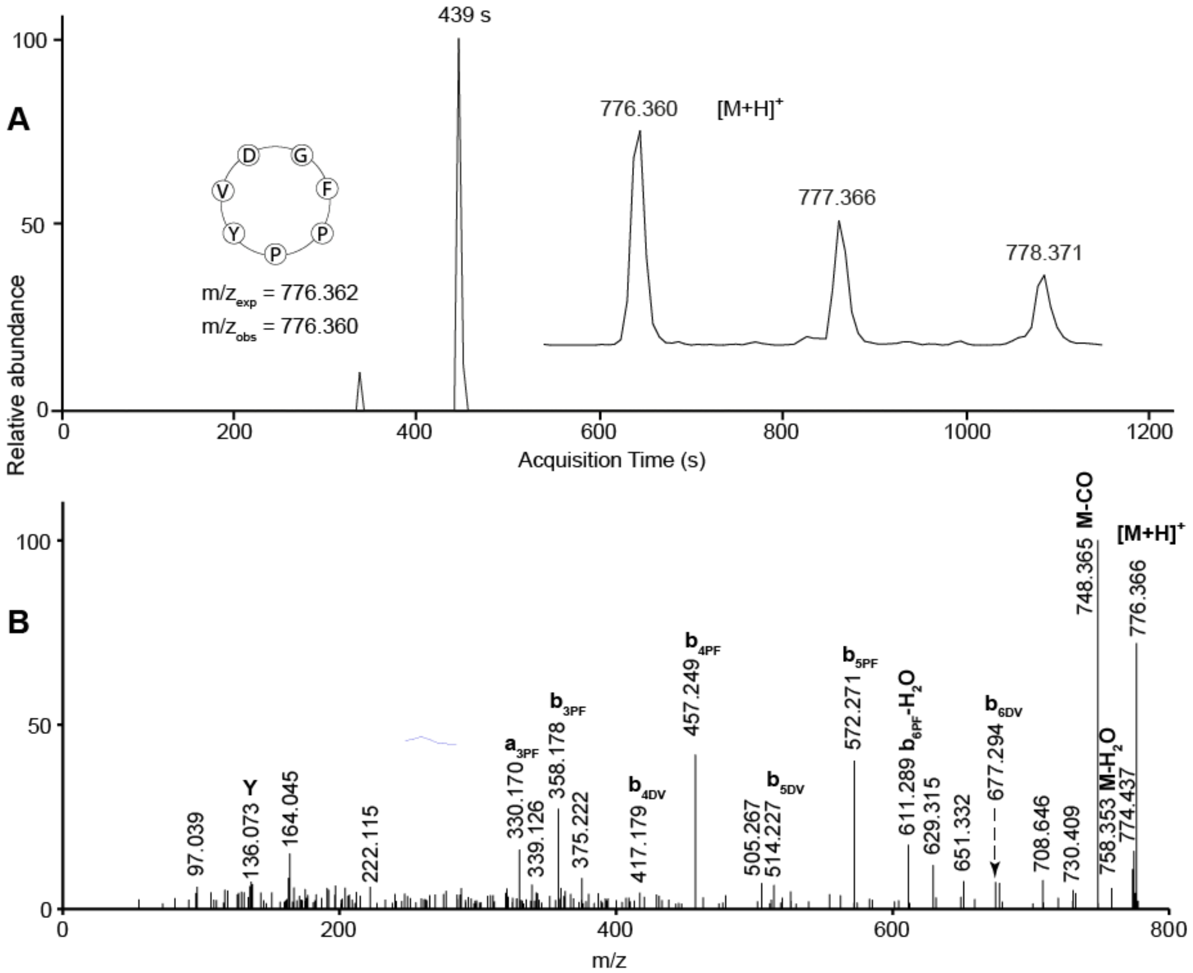

C	G-D	F-G	P-F	P-P	Y-P	V-Y	D-V
G	58.029	776.362	***629.293***	532.241	435.188	272.125	173.056
F	205.098	148.076	776.362	679.309	582.256	419.193	320.125
P	302.150	245.129	98.061	776.362	679.309	516.246	***417.177***
P	399.203	342.182	195.113	98.061	776.362	613.299	***514.230***
Y	562.267	505.245	***358.177***	261.124	164.071	776.362	***677.293***
V	661.335	604.313	***457.245***	360.192	263.140	100.076	776.362
D	776.362	719.340	***572.272***	475.219	378.167	215.103	116.035

Supplemental Figure 19. Q-TOF LC-MS data for PLP-17 in *Buphthalmum salicifolium*. (**A**) Extracted ion chromatogram showing acquisition time of the peptide, peptide sequence with expected and observed mass-to-charge ratios (m/z) and peptide mass spectrum. (**B**) Tandem mass spectrum of the fragmented precursor ion. Immonium ions are denoted by their one-letter residue code. (**C**) Predicted b-ions following ring cleavage. Columns are for each cleavage point. Rows show the mass of the b-ion which contains the residue on the left at its C-terminus. Ions identified in the mass spectrum (either directly, or with loss of H_2O or CO) are shown in ***bold italics***.

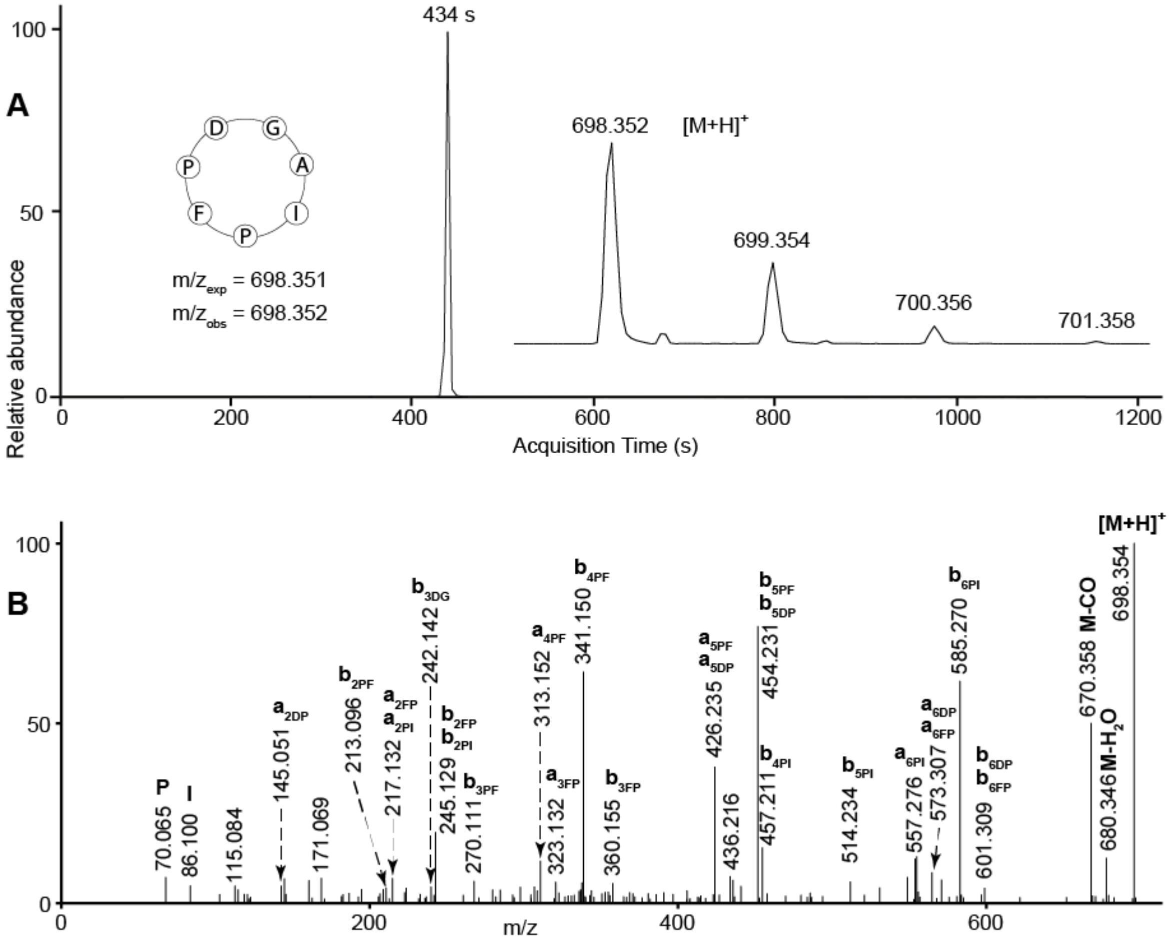

C

	G-D	A-G	I-A	P-I	F-P	P-F	D-P
G	58.029	698.351	627.314	***514.230***	417.177	***270.109***	173.056
A	129.066	72.045	698.351	585.267	488.215	***341.146***	244.093
I	***242.150***	185.129	114.092	698.351	***601.299***	***454.230***	357.177
P	339.203	282.182	211.145	98.061	698.351	551.283	***454.230***
F	486.272	429.250	358.213	***245.129***	148.076	698.351	***601.299***
P	583.324	526.303	455.266	342.182	***245.129***	98.061	698.351
D	698.351	641.330	570.2 93	***457.209***	***360.156***	***213.088***	116.035

Supplemental Figure 20. Q-TOF LC-MS data for PLP-18 in *Buphthalmum salicifolium*. (**A**) Extracted ion chromatogram showing acquisition time of the peptide, peptide sequence with expected and observed mass-to-charge ratios (m/z) and peptide mass spectrum. (**B**) Tandem mass spectrum of the fragmented precursor ion. Immonium ions are denoted by their one-letter residue code. (**C**) Predicted b-ions following ring cleavage. Columns are for each cleavage point. Rows show the mass of the b-ion which contains the residue on the left at its C-terminus. Ions identified in the mass spectrum (either directly, or with loss of H_2O or CO) are shown in ***bold italics***.

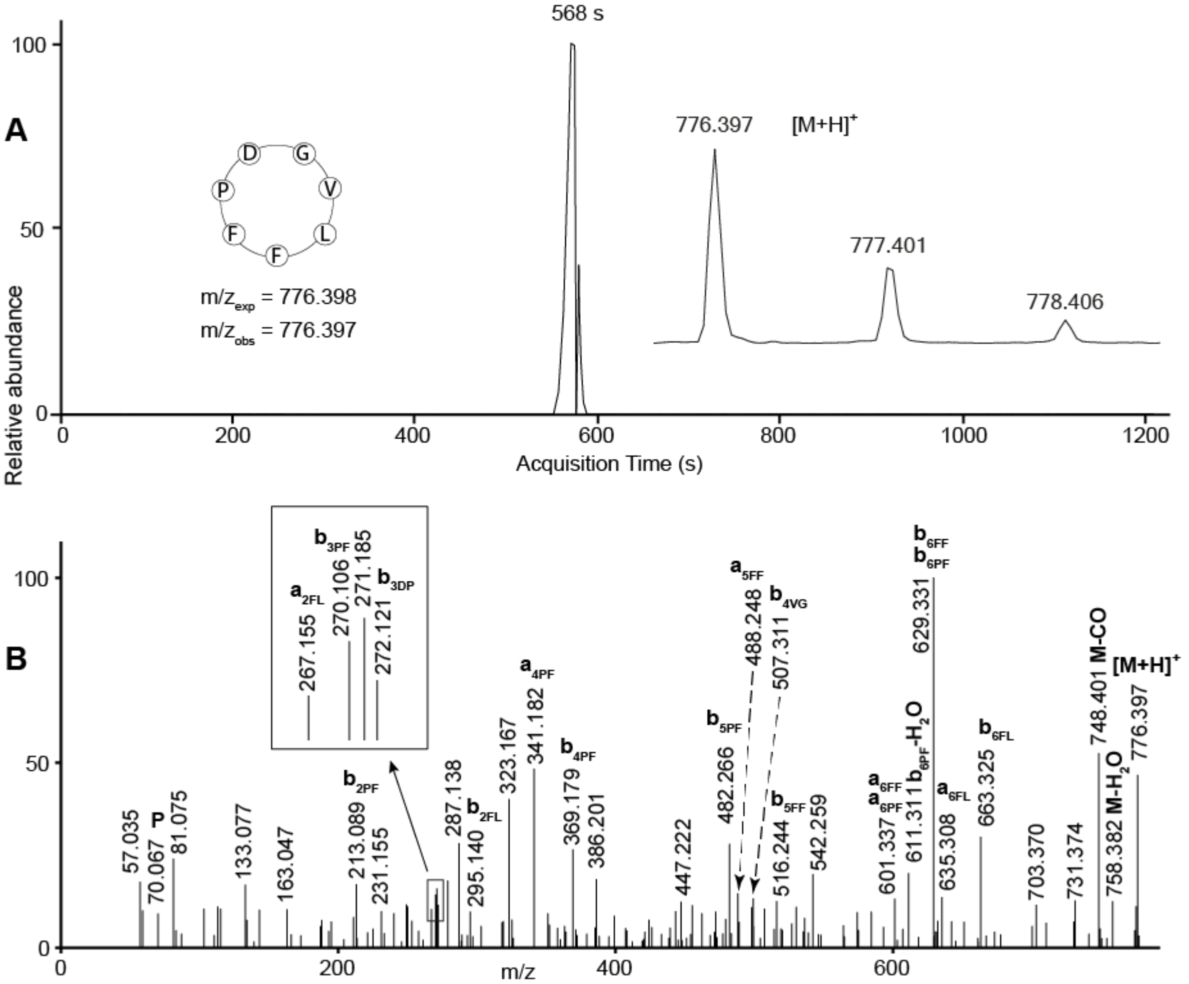

C

	G-D	V-G	L-V	F-L	F-F	P-F	D-P
G	58.029	776.398	677.330	564.246	417.177	***270.109***	173.056
V	157.098	100.076	776.39 8	***663.314***	***516.246***	***369.177***	***272.125***
L	270.182	213.160	114.092	776.398	***629.330***	***482.261***	385.209
F	417.250	360.229	261.160	148.076	776.398	***629.330***	532.277
F	564.319	***507.297***	408.229	***295.145***	148.076	776.398	679.346
P	661.371	604.350	505.281	392.197	245.129	98.061	776.398
D	776.398	719.377	620.308	507.224	360.156	***213.088***	116.035

Supplemental Figure 21. Q-TOF LC-MS data for PLP-19 in *Buphthalmum salicifolium*. (**A**) Extracted ion chromatogram showing acquisition time of the peptide, peptide sequence with expected and observed mass-to-charge ratios (m/z) and peptide mass spectrum. (**B**) Tandem mass spectrum of the fragmented precursor ion. Immonium ions are denoted by their one-letter residue code. (**C**) Predicted b-ions following ring cleavage. Columns are for each cleavage point. Rows show the mass of the b-ion which contains the residue on the left at its C-terminus. Ions identified in the mass spectrum (either directly, or with loss of H_2O or CO) are shown in ***bold italics***.

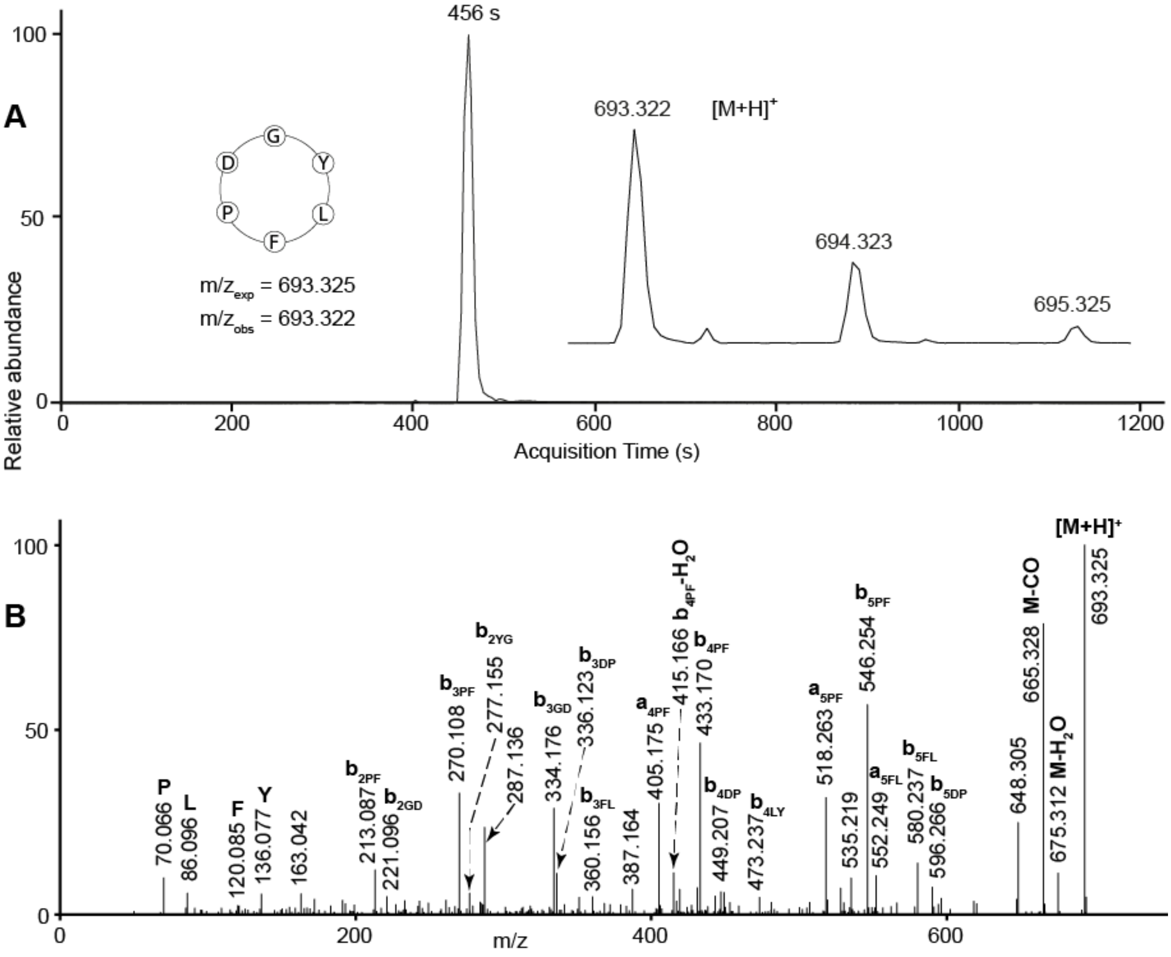

	G-D	Y-G	L-Y	F-L	P-F	D-P
G	58.029	693.325	530.261	417.177	***270.109***	173.056
Y	***221.093***	164.071	693.325	***580.241***	***433.172***	***336.120***
L	***334.177***	***277.155***	114.092	693.325	***546.256***	***449.204***
F	481.245	424.224	261.160	148.076	693.325	***596.272***
P	578.298	521.276	358.213	245.129	98.061	693.325
D	693.325	636.303	***473.240***	***360.156***	***213.088***	116.035

Supplemental Figure 22. Q-TOF LC-MS data for PLP-20 in *Inula racemosa*. (**A**) Extracted ion chromatogram showing acquisition time of the peptide, peptide sequence with expected and observed mass-to-charge ratios (m/z) and peptide mass spectrum. (**B**) Tandem mass spectrum of the fragmented precursor ion. Immonium ions are denoted by their one-letter residue code. (**C**) Predicted b-ions following ring cleavage. Columns are for each cleavage point. Rows show the mass of the b-ion which contains the residue on the left at its C-terminus. Ions identified in the mass spectrum (either directly, or with loss of H_2O or CO) are shown in ***bold italics***.

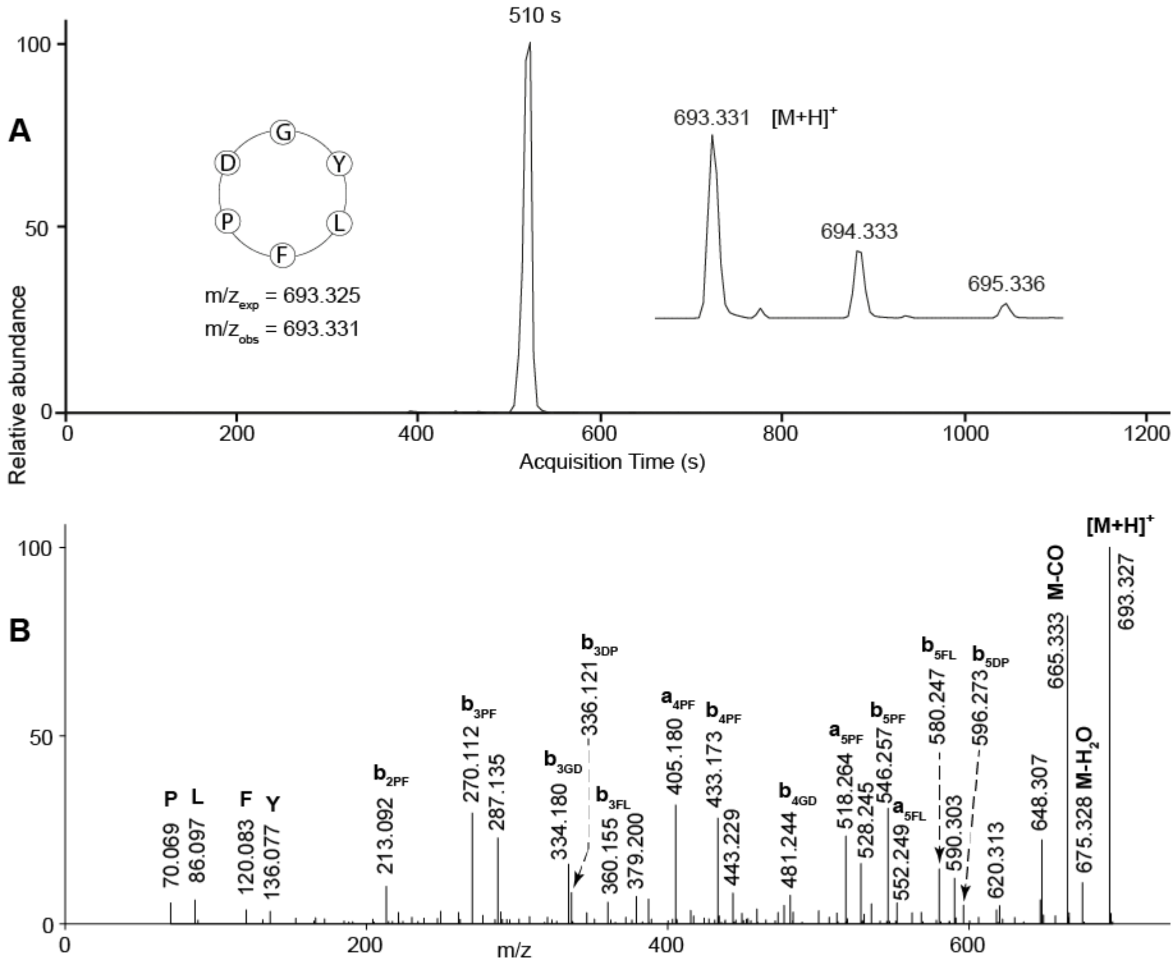

C

	G-D	Y-G	L-Y	F-L	P-F	D-P
G	58.029	693.325	530.261	417.177	***270.109***	173.056
Y	221.093	164.071	693.325	***580.241***	***433.172***	***336.120***
L	***334.177***	277.155	114.092	693.325	***546.256***	449.204
F	***481.245***	424.224	261.160	148.076	693.325	***596.272***
P	578.298	521.276	358.213	245.129	98.061	693.325
D	693.325	636.303	473.240	***360.156***	***213.088***	116.035

Supplemental Figure 23. Q-TOF LC-MS data for PLP-20 in *Inula helenium*. (**A**) Extracted ion chromatogram showing acquisition time of the peptide, peptide sequence with expected and observed mass-to-charge ratios (m/z) and peptide mass spectrum. (**B**) Tandem mass spectrum of the fragmented precursor ion. Immonium ions are denoted by their one-letter residue code. (**C**) Predicted b-ions following ring cleavage. Columns are for each cleavage point. Rows show the mass of the b-ion which contains the residue on the left at its C-terminus. Ions identified in the mass spectrum (either directly, or with loss of H_2O or CO) are shown in ***bold italics***.

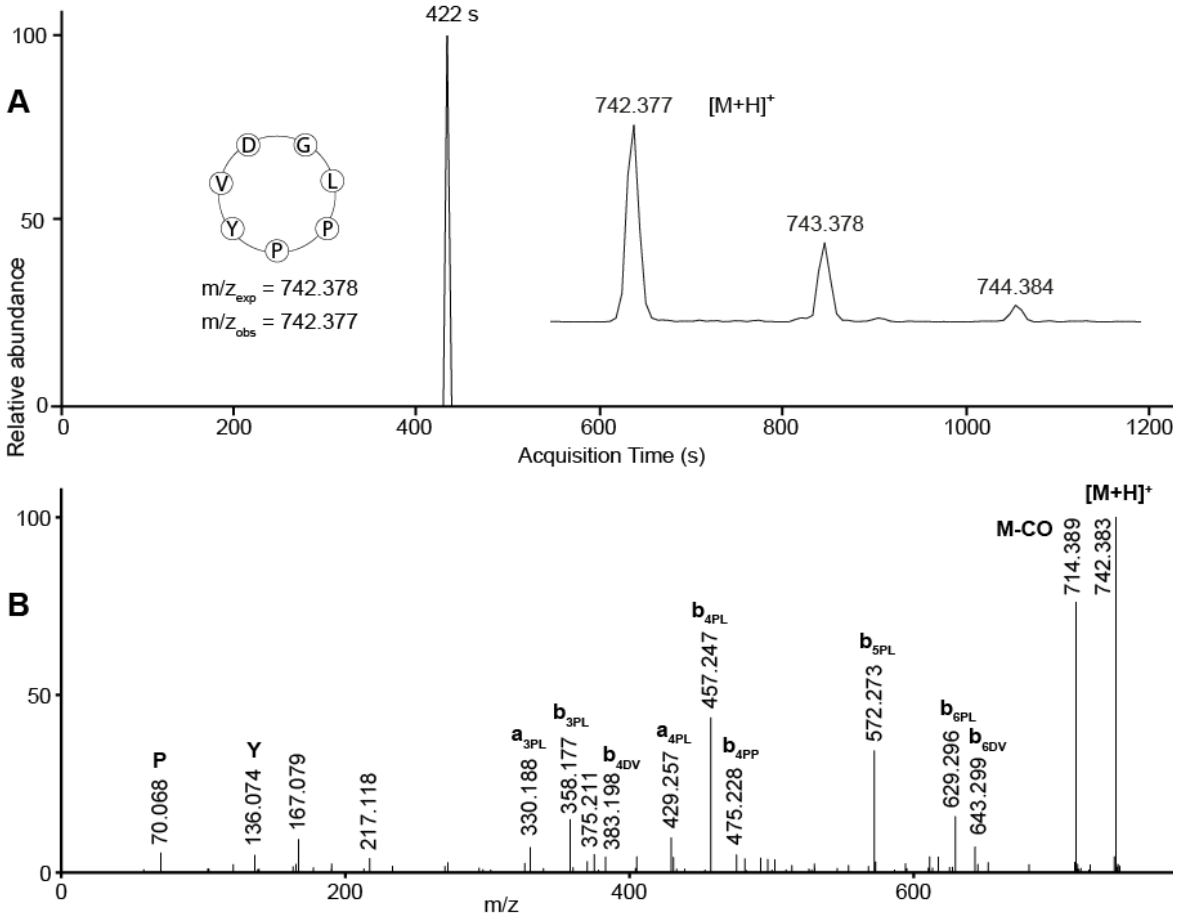

C	G-D	L-G	P-L	P-P	Y-P	V-Y	D-V
G	58.029	742.378	***629.293***	532.241	435.188	272.125	173.056
L	171.113	114.092	742.378	645.325	548.272	385.209	286.140
P	268.166	211.145	98.061	742.378	645.325	482.261	***383.193***
P	365.219	308.197	195.113	98.061	742.378	579.314	480.246
Y	528.282	471.261	***358.177***	261.124	164.071	742.378	***643.309***
V	627.351	570.329	***457.245***	360.192	263.140	100.076	742.378
D	742.378	685.356	***572.272***	***475.219***	378.167	215.103	116.035

Supplemental Figure 24. Q-TOF LC-MS data for PLP-22 in *Inula racemosa*. (**A**) Extracted ion chromatogram showing acquisition time of the peptide, peptide sequence with expected and observed mass-to-charge ratios (m/z) and peptide mass spectrum. (**B**) Tandem mass spectrum of the fragmented precursor ion. Immonium ions are denoted by their one-letter residue code. (**C**) Predicted b-ions following ring cleavage. Columns are for each cleavage point. Rows show the mass of the b-ion which contains the residue on the left at its C-terminus. Ions identified in the mass spectrum (either directly or with loss of CO) are shown in ***bold italics***.

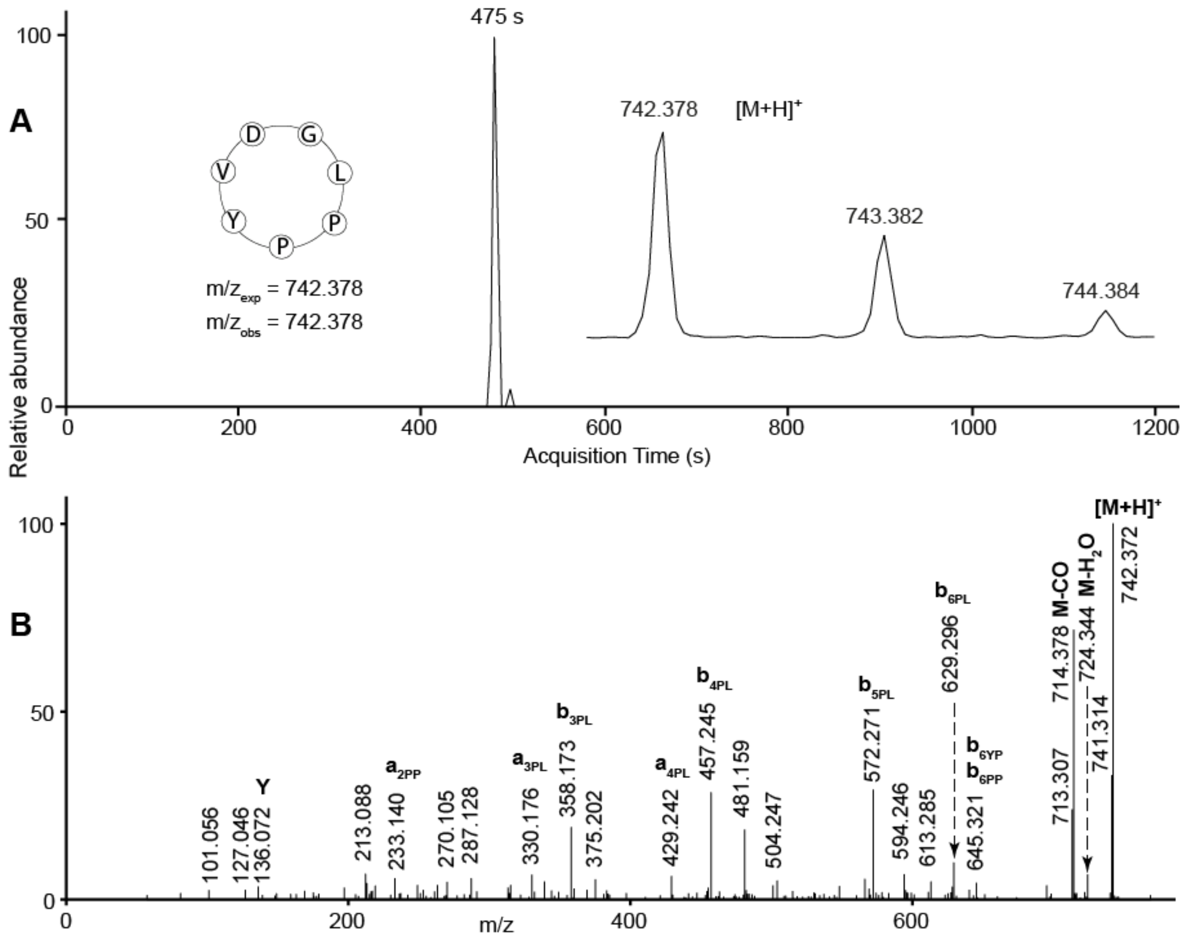

C

	G-D	L-G	P-L	P-P	Y-P	V-Y	D-V
G	58.029	742.378	***629.293***	532.241	435.188	272.125	173.056
L	171.113	114.092	742.378	***645.325***	548.272	385.209	286.140
P	268.166	211.145	98.061	742.378	***645.325***	482.261	383.193
P	365.219	308.197	195.113	98.061	742.378	579.314	480.246
Y	528.282	471.261	***358.177***	261.124	164.071	742.378	643.309
V	627.351	570.329	***457.245***	360.192	263.140	100.076	742.378
D	742.378	685.356	***572.272***	475.219	378.167	215.103	116.035

Supplemental Figure 25. Q-TOF LC-MS data for PLP-22 in *Inula helenium*. (**A**) Extracted ion chromatogram showing acquisition time of the peptide, peptide sequence with expected and observed mass-to-charge ratios (m/z) and peptide mass spectrum. (**B**) Tandem mass spectrum of the fragmented precursor ion. Immonium ions are denoted by their one-letter residue code. (**C**) Predicted b-ions following ring cleavage. Columns are for each cleavage point. Rows show the mass of the b-ion which contains the residue on the left at its C-terminus. Ions identified in the mass spectrum (either directly, or with loss of H_2O or CO) are shown in ***bold italics***.

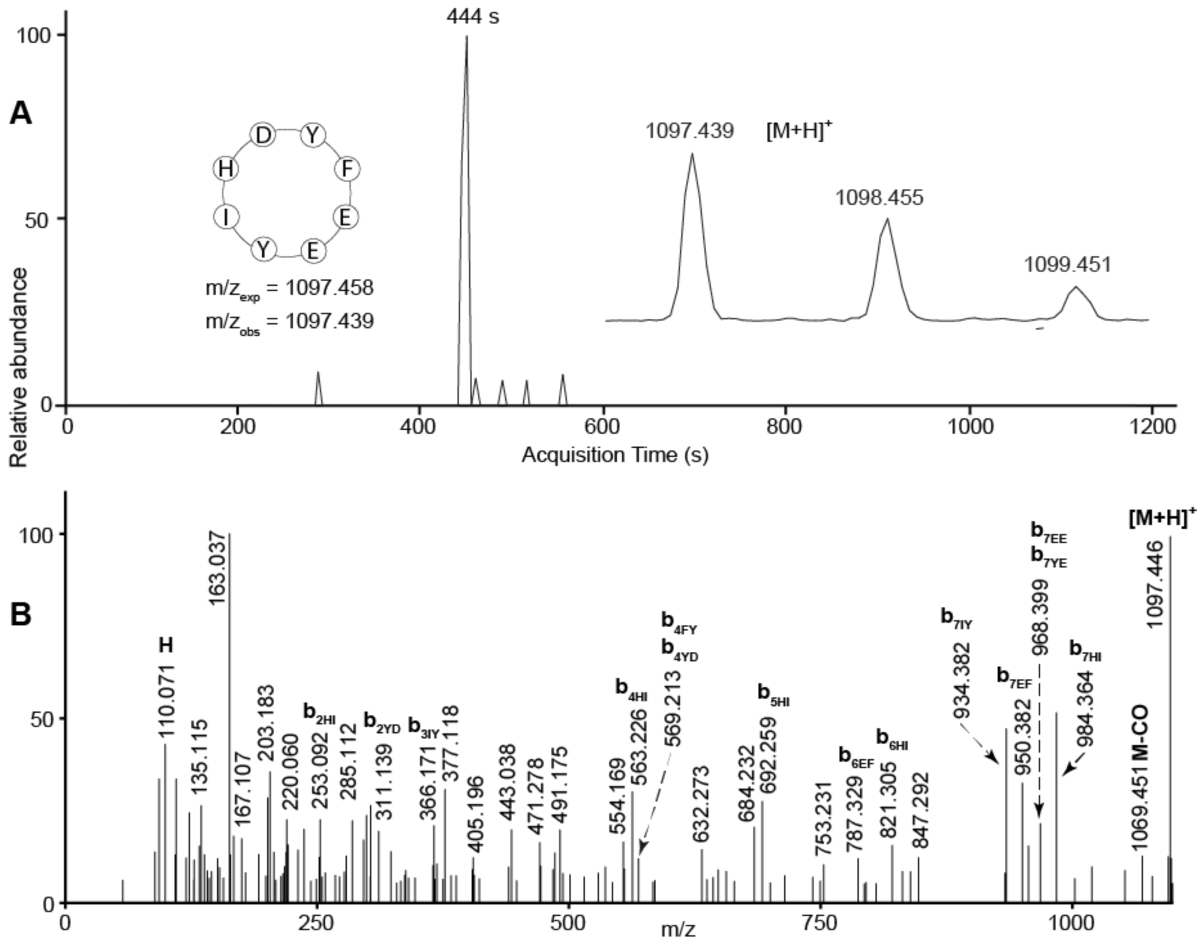

C	Y-D	F-Y	E-F	E-E	Y-E	I-Y	H-I	D-H
Y	164.071	1097.458	***950.390***	821.347	692.304	529.241	416.157	279.098
F	***311.140***	148.076	1097.458	***968.415***	839.373	676.309	***563.225***	426.167
E	440.182	277.119	130.050	1097.458	***968.415***	805.352	***692.268***	555.209
E	***569.225***	406.161	259.093	130.050	1097.458	***934.395***	***821.311***	684.252
Y	732.288	***569.225***	422.156	293.114	164.071	1097.458	***984.374***	847.315
I	845.372	682.309	535.240	406.198	277.155	114.092	1097.458	960.399
H	982.431	819.368	672.299	543.257	414.214	251.151	138.067	1097.458
D	1097.458	934.395	***787.326***	658.284	529.241	***366.178***	***253.094***	116.035

Supplemental Figure 26. Q-TOF LC-MS data for PLP-23 in *Othonna arborescens*. (**A**) Extracted ion chromatogram showing acquisition time of the peptide, peptide sequence with expected and observed mass-to-charge ratios (m/z) and peptide mass spectrum. (**B**) Tandem mass spectrum of the fragmented precursor ion. Immonium ions are denoted by their one-letter residue code. (**C**) Predicted b-ions following ring cleavage. Columns are for each cleavage point. Rows show the mass of the b-ion which contains the residue on the left at its C-terminus. Ions identified in the mass spectrum (either directly, or with loss of H_2O or CO) are shown in ***bold italics***.

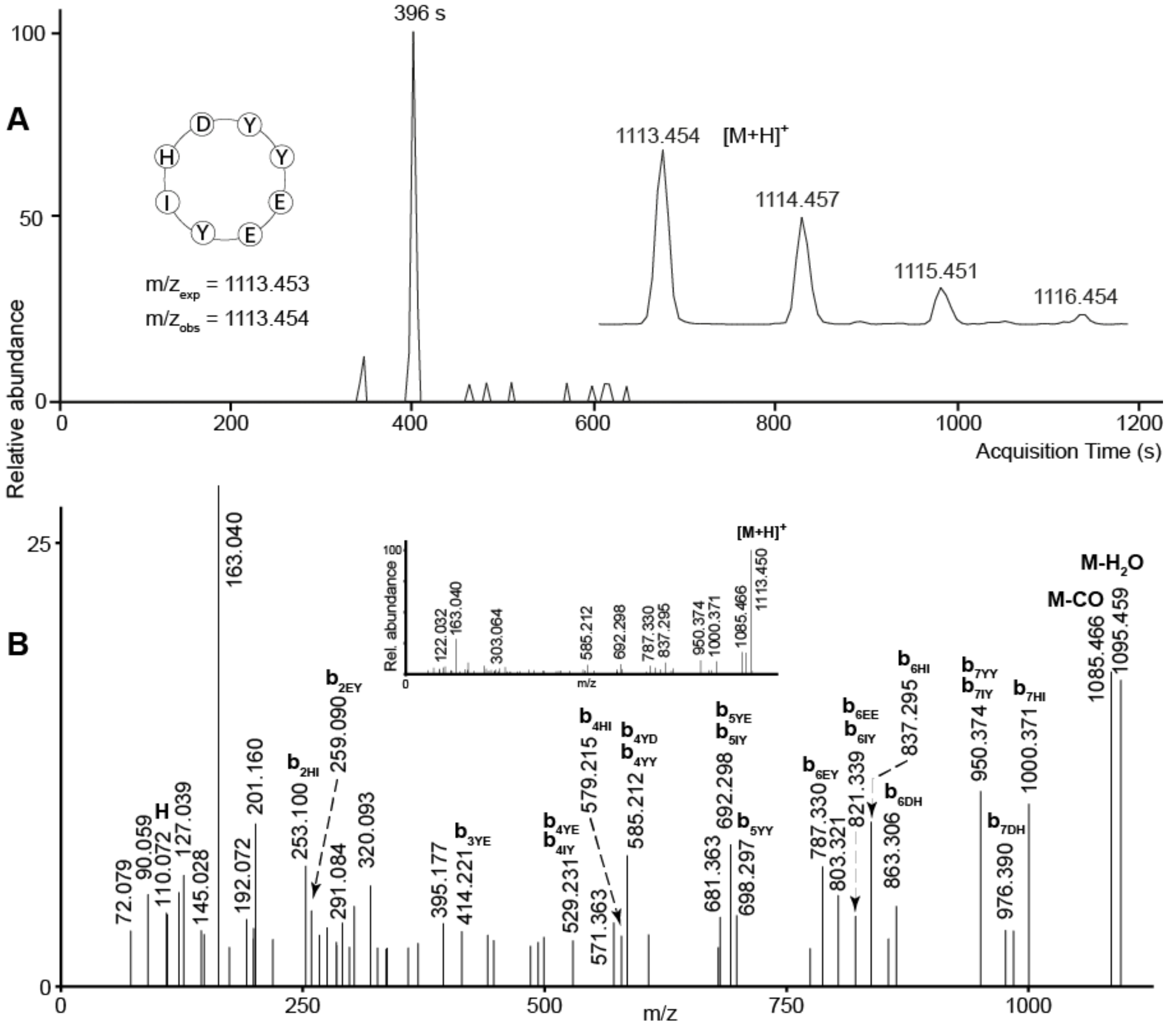

C	Y-D	Y-Y	E-Y	E-E	Y-E	I-Y	H-I	D-H
Y	164.071	1113.453	***950.390***	***821.347***	***692.304***	***529.241***	416.157	279.098
Y	327.134	164.071	1113.453	984.410	855.368	***692.304***	***579.220***	442.161
E	456.177	293.114	130.050	1113.453	984.410	***821.347***	708.263	571.204
E	*585.220*	422.156	***259.093***	130.050	1113.453	950.390	***837.306***	700.247
Y	748.283	***585.220***	422.156	293.114	164.071	1113.453	***1000.369***	***863.310***
I	861.367	***698.304***	535.240	406.198	277.155	114.092	1113.453	976.394
H	998.426	835.363	672.299	543.257	***414.214***	251.151	138.067	1113.453
D	1113.453	***950.390***	***787.326***	658.284	***529.241***	366.178	***253.094***	116.035

Supplemental Figure 27. Q-TOF LC-MS data for PLP-25 in *Othonna arborescens*. (**A**) Extracted ion chromatogram showing acquisition time of the peptide, peptide sequence with expected and observed mass-to-charge ratios (m/z) and peptide mass spectrum. (**B**) Tandem mass spectrum of the fragmented precursor ion. Immonium ions are denoted by their one-letter residue code. (**C**) Predicted b-ions following ring cleavage. Columns are for each cleavage point. Rows show the mass of the b-ion which contains the residue on the left at its C-terminus. Ions identified in the mass spectrum (either directly, or with loss of H_2O or CO) are shown in ***bold italics***.

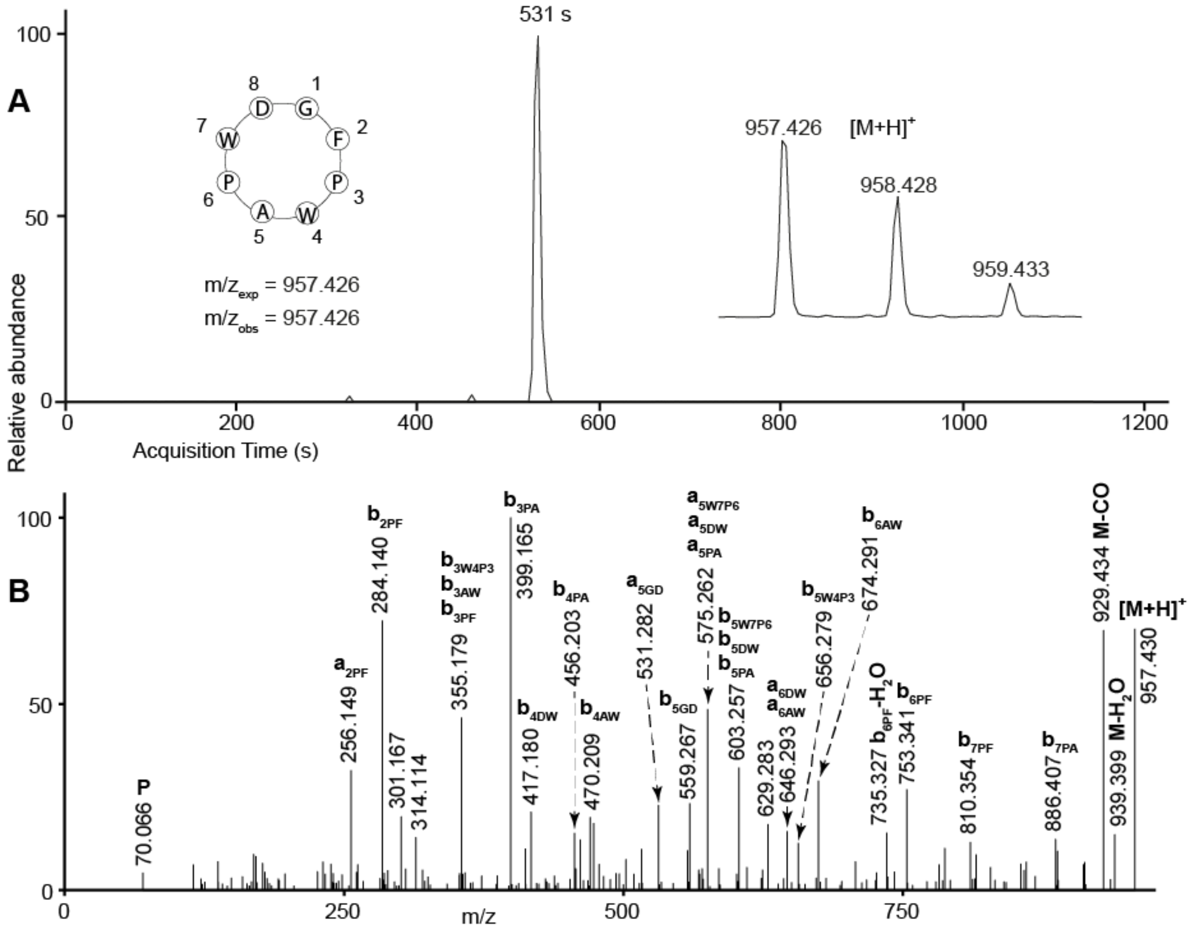

C

	D-W	G-D	F-G	P-F	W^4-P^3	A-W	P-A	W^7-P^6
D	116.035	957.426	900.404	***753.336***	***656.283***	***470.204***	***399.167***	302.114
G	173.056	58.029	957.426	***810.357***	713.305	527.225	***456.188***	359.136
F	320.125	205.098	148.076	957.426	860.373	***674.294***	***603.257***	506.204
P	***417.177***	302.150	245.129	98.061	957.426	771.347	700.309	***603.257***
W	***603.257***	488.230	431.208	***284.140***	187.087	957.426	***886.389***	789.336
A	674.294	***559.267***	502.245	***355.177***	258.124	72.045	957.426	860.373
P	771.347	656.320	599.298	452.230	***355.177***	169.098	98.061	957.426
W	957.426	842.399	785.377	638.309	541.256	***355.177***	284.140	187.087

Supplemental Figure 28. Q-TOF LC-MS data for PLP-26 in *Melampodium paludosum*. (**A**) Extracted ion chromatogram showing acquisition time of the peptide, peptide sequence with expected and observed mass-to-charge ratios (m/z) and peptide mass spectrum. (**B**) Tandem mass spectrum of the fragmented precursor ion. Immonium ions are denoted by their one-letter residue code. (**C**) Predicted b-ions following ring cleavage. Columns are for each cleavage point. Rows show the mass of the b-ion which contains the residue on the left at its C-terminus. Ions identified in the mass spectrum (either directly, or with loss of H_2O or CO) are shown in ***bold italics***.

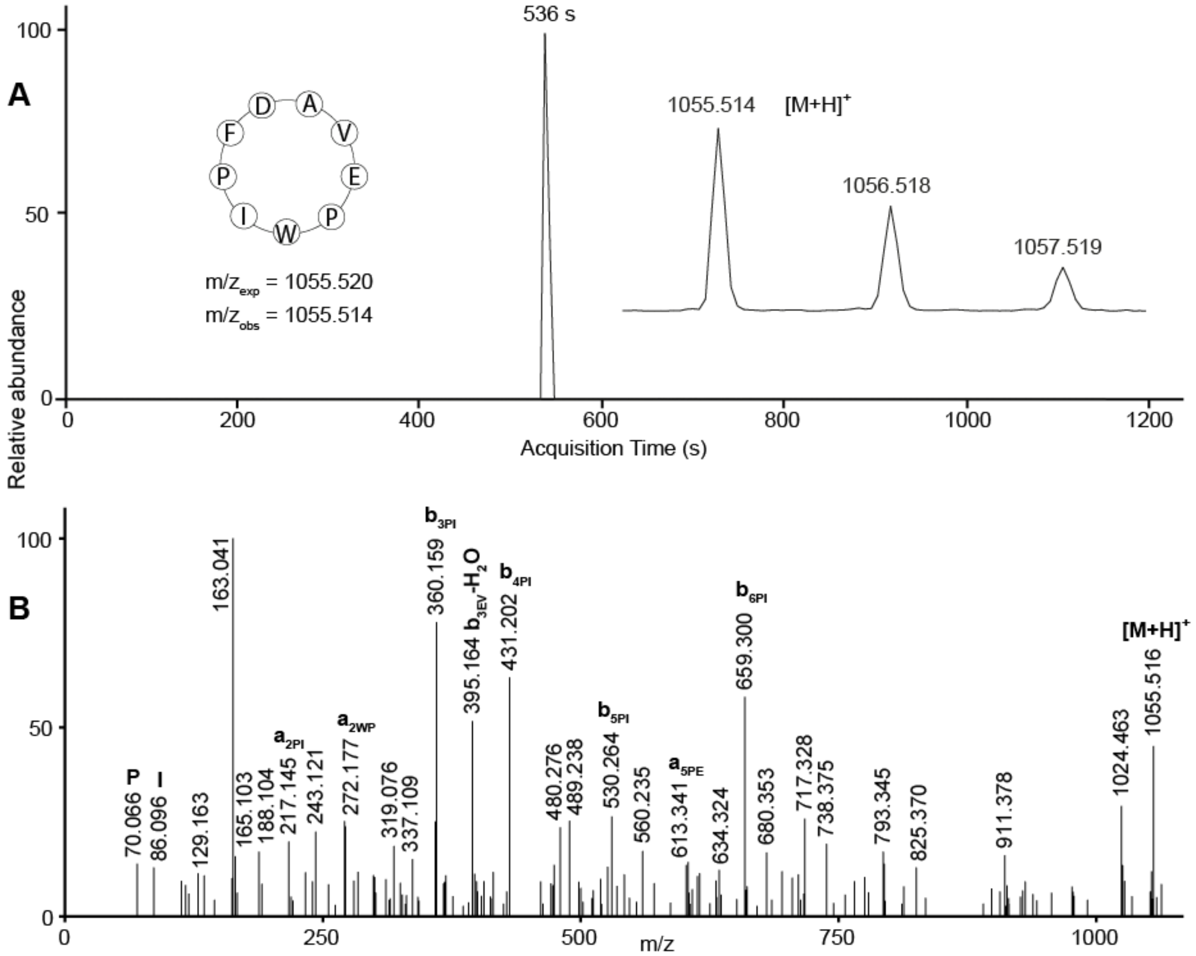

C

	A-D	V-A	E-V	P-E	W-P	I-W	P-I	F-P	D-F
A	72.045	1055.520	956.452	827.409	730.356	544.277	***431.193***	334.140	187.072
V	171.113	100.076	1055.520	926.478	829.425	643.346	***530.261***	433.209	286.140
E	300.156	229.119	130.050	1055.520	958.467	772.388	***659.304***	562.251	415.183
P	397.209	326.172	227.103	98.061	1055.520	869.441	756.357	659.304	512.236
W	583.288	512.251	413.182	284.140	187.087	1055.520	942.436	845.383	698.315
I	696.372	625.335	526.267	397.224	***300.171***	114.092	1055.520	958.467	811.399
P	793.425	722.388	623.319	494.277	397.224	211.145	98.061	1055.520	908.452
F	940.493	869.456	770.388	***641.345***	544.292	358.213	***245.129***	148.076	1055.520
D	1055.520	984.483	885.415	756.372	659.319	473.240	***360.156***	263.103	116.035

Supplemental Figure 29. Q-TOF LC-MS data for PLP-27 in *Melampodium paludosum*. (**A**) Extracted ion chromatogram showing acquisition time of the peptide, peptide sequence with expected and observed mass-to-charge ratios (m/z) and peptide mass spectrum. (**B**) Tandem mass spectrum of the fragmented precursor ion. Immonium ions are denoted by their one-letter residue code. (**C**) Predicted b-ions following ring cleavage. Columns are for each cleavage point. Rows show the mass of the b-ion which contains the residue on the left at its C-terminus. Ions identified in the mass spectrum (either directly, or with loss of H_2O or CO) are shown in ***bold italics***.

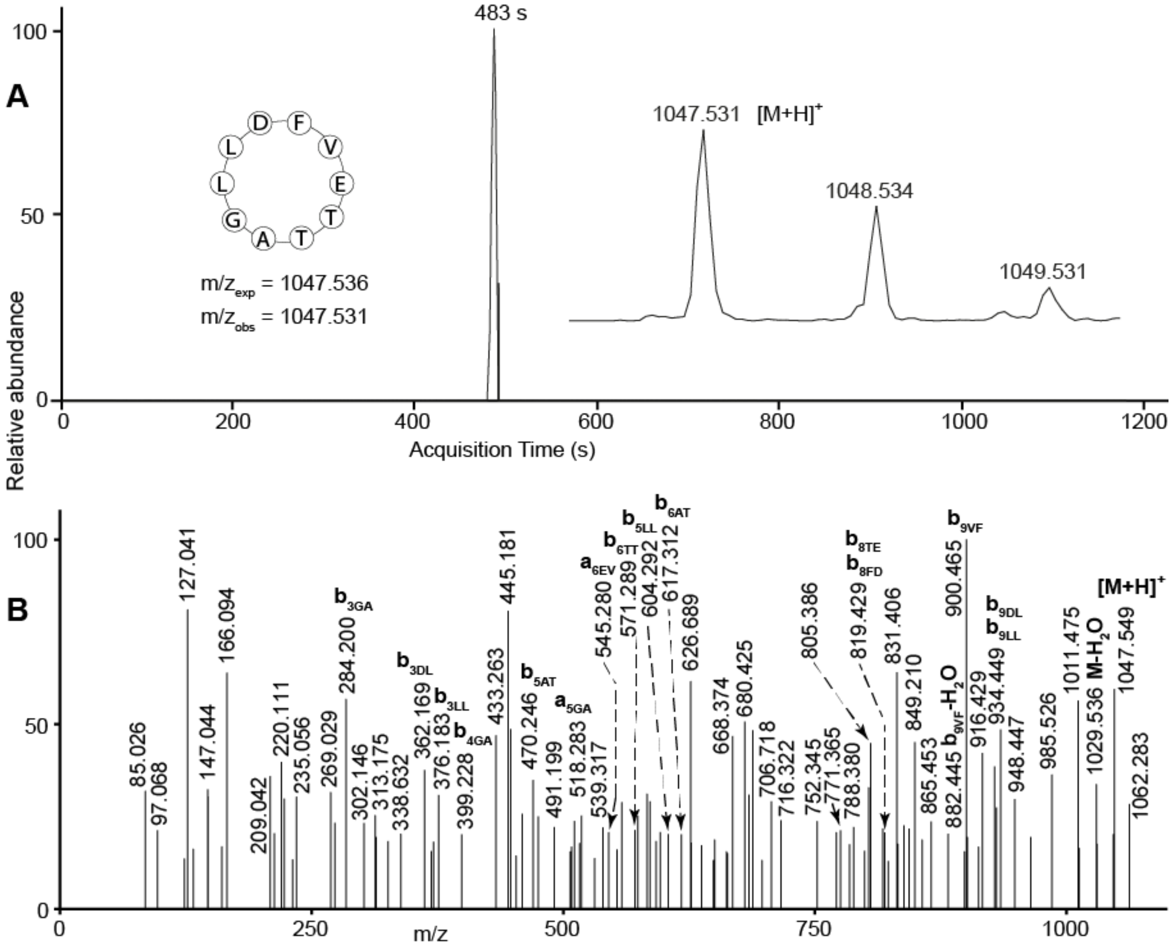

C

	F-D	V-F	E-V	T-E	T-T	A-T	G-A	L-G	L-L	D-L
F	148.076	1047.536	948.468	***819.425***	718.378	***617.330***	***546.293***	489.271	***376.187***	263.103
V	247.145	100.076	1047.536	918.494	817.446	716.398	645.361	588.340	475.256	***362.172***
E	376.187	229.119	130.050	1047.536	946.489	845.441	774.404	717.382	***604.298***	491.214
T	477.235	330.167	231.098	102.056	1047.536	946.489	875.451	818.430	705.346	592.262
T	578.283	431.214	332.146	203.103	102.056	1047.536	976.499	919.478	806.394	693.310
A	649.320	502.251	403.183	274.140	173.093	72.045	1047.536	990.515	877.431	764.347
G	706.341	559.273	460.204	331.162	230.114	129.066	58.029	1047.536	***934.452***	821.368
L	***819.425***	672.357	***573.288***	444.246	343.198	242.150	171.113	114.092	1047.536	***934.452***
L	932.509	785.441	686.372	557.330	456.282	355.235	***284.197***	227.176	114.092	1047.536
D	1047.536	***900.468***	801.399	672.357	***571.309***	***470.261***	***399.224***	342.203	229.119	116.035

Supplemental Figure 30. Q-TOF LC-MS data for PLP-28 in *Steirodiscus tagetes*. (**A**) Extracted ion chromatogram showing acquisition time of the peptide, peptide sequence with expected and observed mass-to-charge ratios (m/z) and peptide mass spectrum. (**B**) Tandem mass spectrum of the fragmented precursor ion. Immonium ions are denoted by their one-letter residue code. (**C**) Predicted b-ions following ring cleavage. Columns are for each cleavage point. Rows show the mass of the b-ion which contains the residue on the left at its C-terminus. Ions identified in the mass spectrum (either directly, or with loss of H_2O or CO) are shown in ***bold italics***.

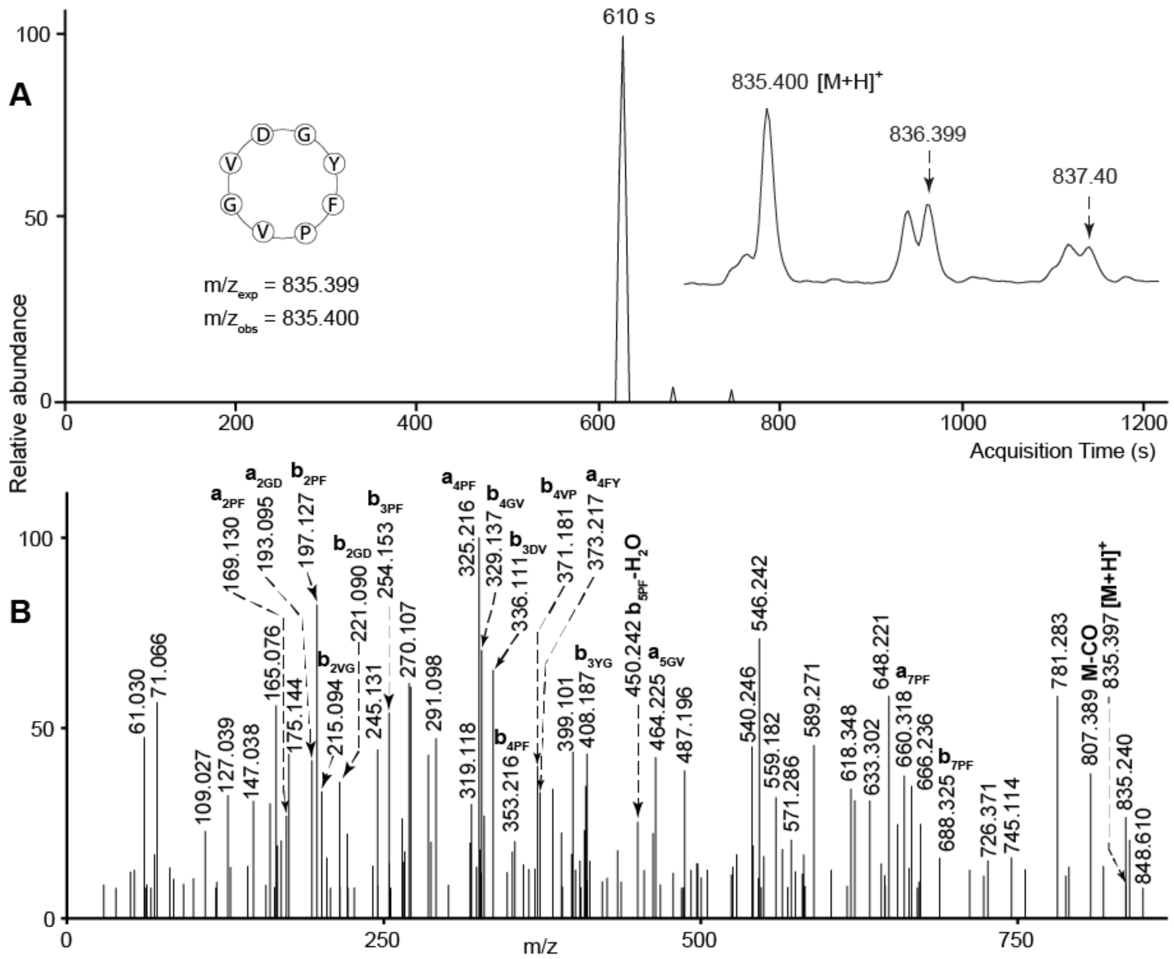

C	G-D	Y-G	F-Y	P-F	V-P	G-V	V-G	D-V
G	58.029	835.399	672.336	525.267	428.215	***329.146***	272.125	173.056
Y	***221.093***	164.071	835.399	***688.331***	591.278	***492.209***	435.188	336.120
F	368.161	311.140	148.076	835.399	738.346	639.278	582.256	483.188
P	465.214	***408.192***	245.129	98.061	835.399	736.331	679.309	580.241
V	564.282	507.261	344.197	***197.129***	100.076	835.399	778.378	679.309
G	621.304	564.282	***401.219***	***254.150***	157.098	58.029	835.399	736.331
V	720.372	663.351	500.287	***353.219***	256.166	157.098	100.076	835.399
D	835.399	778.378	615.314	***468.246***	***371.193***	272.125	***215.103***	116.035

Supplemental Figure 31. Q-TOF LC-MS data for PLP-29 in *Steirodiscus tagetes*. (**A**) Extracted ion chromatogram showing acquisition time of the peptide, peptide sequence with expected and observed mass-to-charge ratios (m/z) and peptide mass spectrum. (**B**) Tandem mass spectrum of the fragmented precursor ion. Immonium ions are denoted by their one-letter residue code. (**C**) Predicted b-ions following ring cleavage. Columns are for each cleavage point. Rows show the mass of the b-ion which contains the residue on the left at its C-terminus. Ions identified in the mass spectrum (either directly, or with loss of H_2O or CO) are shown in ***bold italics***.

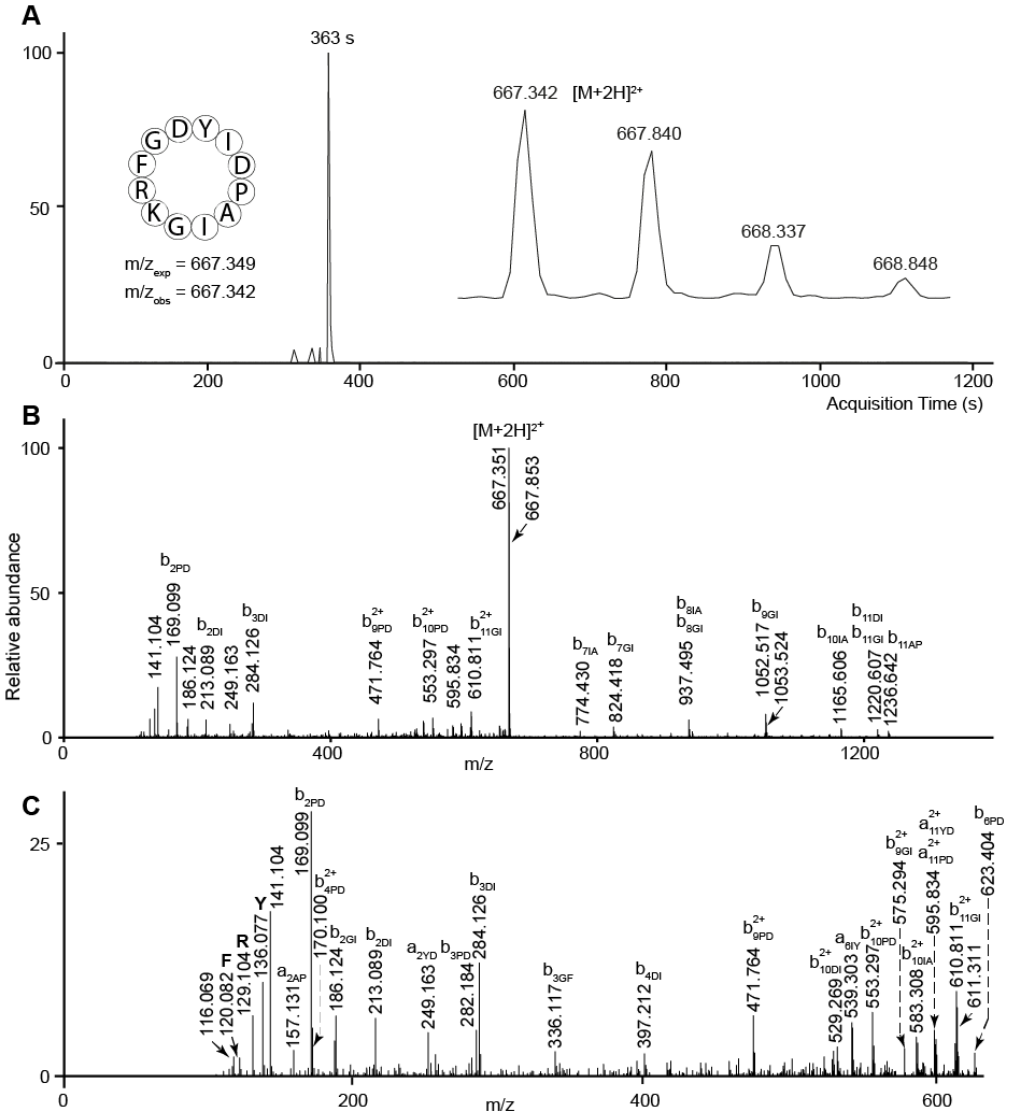

D	Y-D	I-Y	D-I	P-D	A-P	I-A	G-I	K-G	R-K	F-R	G-F	D-G
Y	164.07	1333.69	***1220.61***	1105.58	1008.53	937.49	***824.41***	767.38	639.29	483.19	336.12	279.10
I	***277.16***	114.09	1333.69	1218.66	1121.61	1050.57	937.49	880.47	752.37	596.27	449.20	392.18
D	392.18	229.12	116.03	1333.69	***1236.64***	***1165.60***	***1052.52***	995.50	867.40	711.30	564.23	507.21
P	489.23	326.17	***213.09***	98.06	1333.69	1262.65	1149.57	1092.55	964.45	808.35	661.28	604.26
A	560.27	397.21	***284.12***	***169.10***	72.04	1333.69	***1220.61***	1163.58	1035.49	879.39	732.32	675.30
I	673.36	510.29	***397.21***	***282.18***	***185.13***	114.09	1333.69	1276.67	1148.57	992.47	845.40	788.38
G	730.38	***567.31***	454.23	339.20	242.15	171.11	58.03	1333.69	1205.60	1049.49	902.43	845.40
K	858.47	695.41	582.33	467.30	370.25	299.21	***186.12***	129.10	1333.69	1177.59	1030.52	973.50
R	1014.57	851.51	738.43	***623.40***	526.35	455.31	342.23	285.20	157.11	1333.69	1186.62	1129.60
F	1161.64	998.58	885.49	770.47	673.41	602.38	489.29	432.27	304.18	148.08	1333.69	1276.67
G	1218.66	1055.60	942.52	827.49	730.44	659.40	546.32	489.29	361.20	205.10	58.03	1333.69
D	1333.69	1170.63	1057.54	942.52	845.46	***774.43***	661.34	604.32	476.23	320.12	173.06	116.03

E	Y-D	I-Y	D-I	P-D	A-P	I-A	G-I	K-G	R-K	F-R	G-F	D-G
Y	82.539	667.349	610.807	***553.294***	504.767	469.249	412.707	384.196	320.148	242.098	168.564	140.053
I	139.082	57.550	667.349	***609.836***	561.309	525.791	469.249	440.738	376.690	298.640	225.106	196.595
D	196.595	115.063	58.521	667.349	618.823	***583.304***	526.762	498.251	434.204	356.153	282.619	254.108
P	245.121	163.590	107.048	49.534	667.349	631.831	***575.289***	546.778	482.730	404.680	331.146	302.635
A	280.640	199.108	142.566	85.053	36.526	667.349	***610.807***	582.296	518.249	440.198	366.664	338.153
I	337.182	255.650	199.108	141.595	93.068	57.550	667.349	638.838	574.791	496.740	423.206	394.695
G	365.693	284.161	227.619	***170.106***	121.579	86.061	29.519	667.349	603.302	525.251	451.717	423.206
K	429.740	348.209	291.666	234.153	185.627	150.108	93.566	65.055	667.349	589.299	515.764	487.254
R	507.791	426.259	369.717	312.204	263.677	228.159	171.617	143.106	79.058	667.349	593.815	565.304
F	581.325	499.793	443.251	385.738	337.211	301.693	245.151	216.640	152.593	74.542	667.349	638.838
G	***609.836***	528.304	471.762	414.248	365.722	330.204	273.662	245.151	181.103	103.053	29.519	667.349
D	667.349	585.817	***529.275***	***471.762***	423.236	387.717	331.175	302.664	238.617	160.566	87.032	58.521

Supplemental Figure 32. Q-TOF LC-MS data for PLP-30 in *Cosmos bipinnatus*. (**A**) Extracted ion chromatogram showing acquisition time of the peptide, peptide sequence with expected and observed mass-to-charge ratios (m/z) and peptide mass spectrum. (**B**) Tandem mass spectrum of the fragmented precursor ion. (**C**) Lower half of tandem mass spectrum expanded. Immonium ions are denoted by their one-letter residue code. (**D**) Predicted b-ions following ring cleavage. Columns are for each cleavage point. Rows show the mass of the b-ion which contains the residue on the left at its C-terminus. Ions identified in the mass spectrum (either directly, or with loss of H_2O or CO) are shown in ***bold italics***. (**E**) As **D**, but for 2+ b-ions.

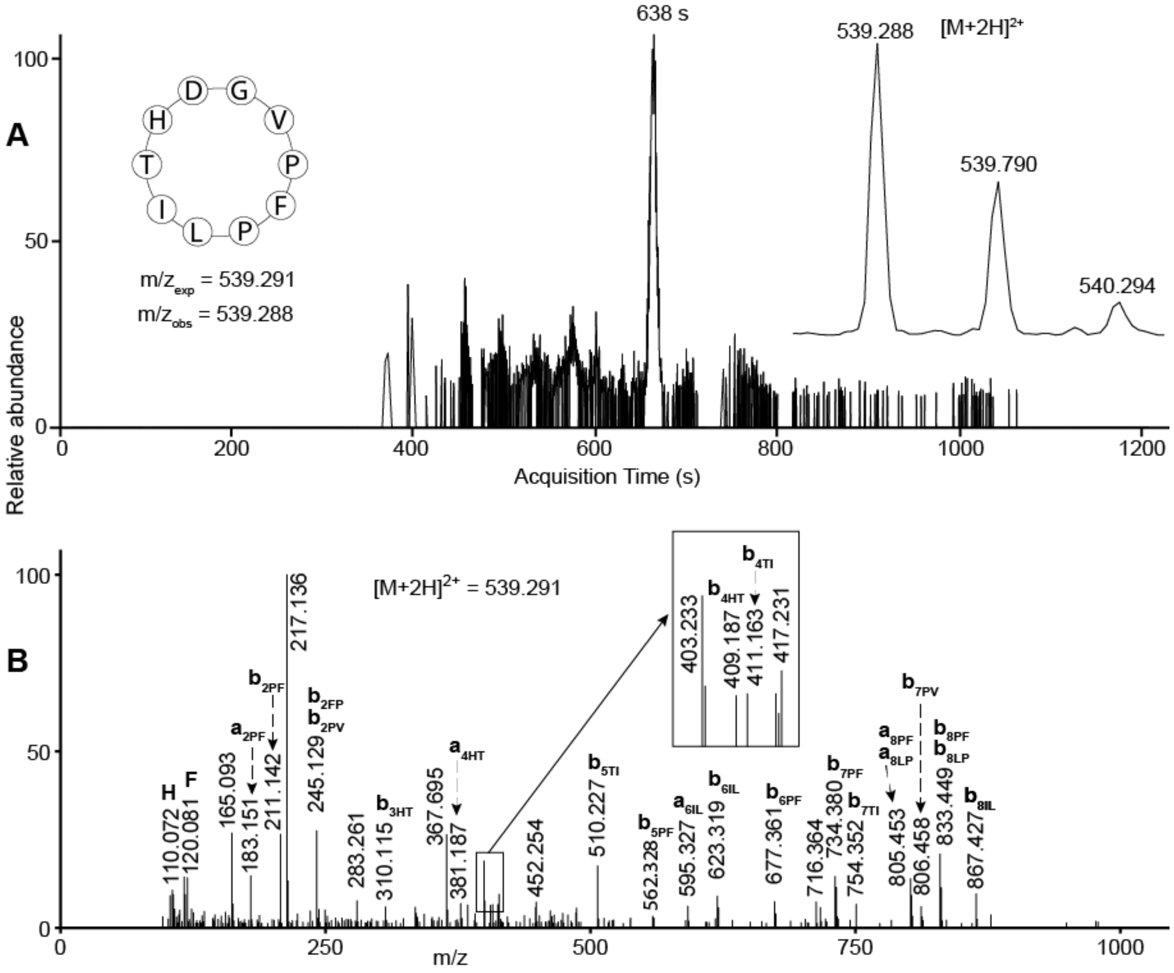

C

	G-D	V-G	P-V	F-P	P-F	L-P	I-L	T-I	H-T	D-H
G	58.029	1077.573	978.505	881.452	***734.384***	637.331	524.247	***411.163***	***310.115***	173.056
V	157.098	100.076	1077.573	980.521	***833.452***	736.399	***623.315***	***510.231***	***409.184***	272.125
P	254.150	197.129	98.061	1077.573	930.505	***833.452***	720.368	607.284	506.236	369.177
F	401.219	344.197	***245.129***	148.076	1077.573	980.521	***867.436***	***754.352***	653.305	516.246
P	498.272	441.250	342.182	***245.129***	98.061	1077.573	964.489	851.405	750.357	613.299
L	611.356	554.334	455.266	358.213	***211.145***	114.092	1077.573	964.489	863.442	726.383
I	724.440	667.418	568.350	471.297	324.229	227.176	114.092	1077.573	976.526	839.467
T	825.487	768.466	669.398	572.345	425.276	328.224	215.140	102.056	1077.573	940.514
H	962.546	905.525	***806.456***	709.404	***562.335***	465.283	352.198	239.114	138.067	1077.573
D	1077.573	1020.552	921.483	824.431	***677.362***	580.309	467.225	354.141	253.094	116.035

Supplemental Figure 33. Q-TOF LC-MS data for PLP-31 in *Engelmannia peristenia*. (**A**) Extracted ion chromatogram showing acquisition time of the peptide, peptide sequence with expected and observed mass-to-charge ratios (m/z) and peptide mass spectrum. (**B**) Tandem mass spectrum of the fragmented precursor ion. Immonium ions are denoted by their one-letter residue code. (**C**) Predicted b-ions following ring cleavage. Columns are for each cleavage point. Rows show the mass of the b-ion which contains the residue on the left at its C-terminus. Ions identified in the mass spectrum (either directly, or with loss of H_2O or CO) are shown in ***bold italics***.

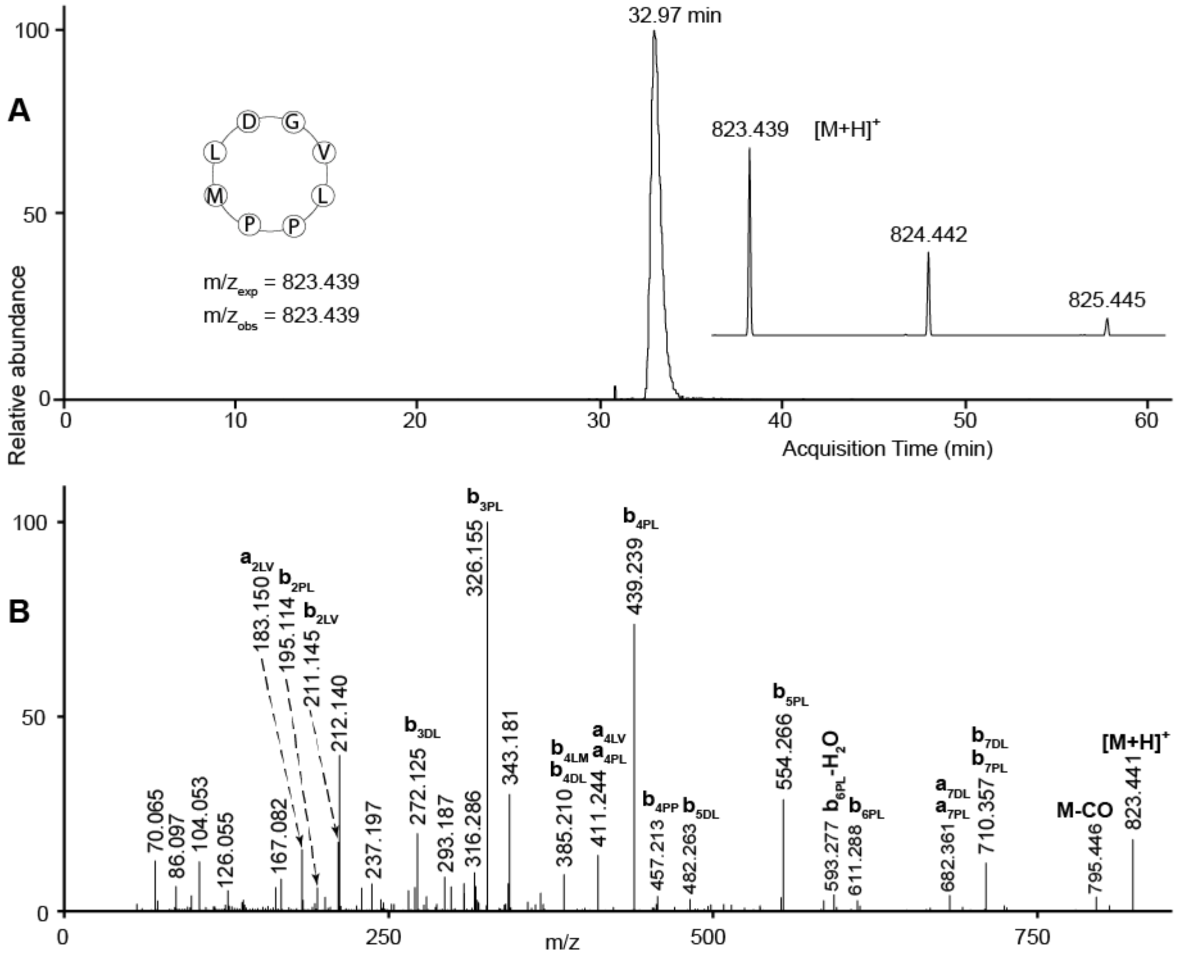

C

	G-D	V-G	L-V	P-L	P-P	M-P	L-M	D-L
G	58.029	823.439	724.370	***611.286***	514.234	417.181	286.140	173.056
V	157.098	100.076	823.439	***710.355***	613.302	516.249	***385.209***	***272.125***
L	270.182	213.160	114.092	823.439	726.386	629.333	498.293	***385.209***
P	367.235	310.213	***211.145***	98.061	823.439	726.386	595.346	***482.261***
P	464.287	407.266	308.197	***195.113***	98.061	823.439	692.398	579.314
M	595.328	538.306	439.238	***326.154***	229.101	132.048	823.439	***710.355***
L	708.412	651.390	552.322	***439.238***	342.185	245.132	114.092	823.439
D	823.439	766.417	667.349	***554.265***	***457.212***	360.159	229.119	116.035

Supplemental Figure 34. Orbitrap LC-MS data for PLP-32 in *Arnica chamissonis***.** (**A**) Extracted ion chromatogram showing acquisition time of the peptide, peptide sequence with expected and observed mass-to-charge ratios (m/z) and peptide mass spectrum. (**B**) Tandem mass spectrum of the fragmented precursor ion. Immonium ions are denoted by their one-letter residue code. (**C**) Predicted b-ions following ring cleavage. Columns are for each cleavage point. Rows show the mass of the b-ion which contains the residue on the left at its C-terminus. Ions identified in the mass spectrum (either directly, or with loss of H_2O or CO) are shown in ***bold italics***.

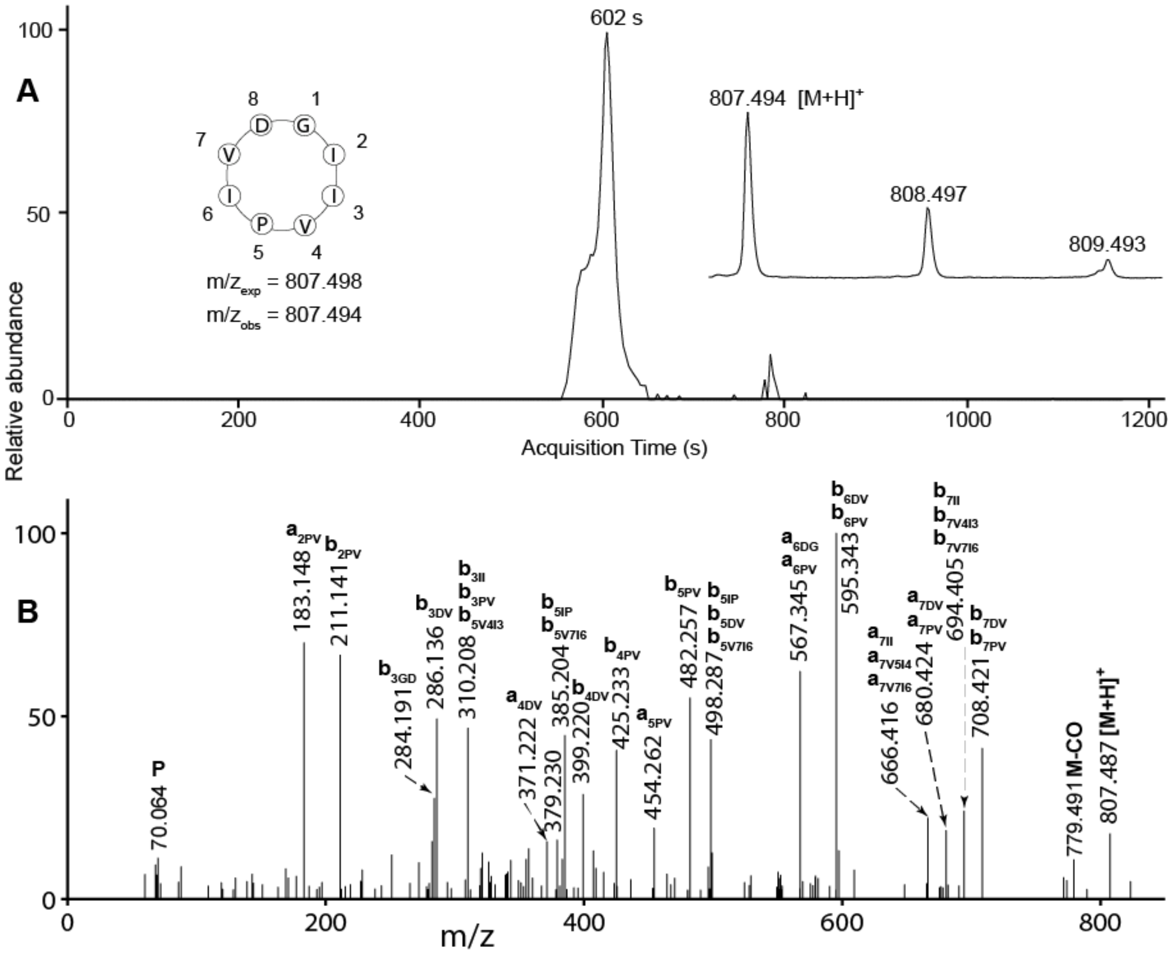

C

	G-D	I-G	I-I	V^4-I^3	P-V	I-P	V^7-I^6	D-V
G	58.029	807.498	***694.414***	581.330	***482.261***	***385.209***	272.125	173.056
I	171.113	114.092	807.498	***694.414***	***595.346***	***498.293***	***385.209***	***286.140***
I	***284.197***	227.176	114.092	807.498	***708.430***	611.377	***498.293***	***399.224***
V	383.266	326.244	213.160	100.076	***807.498***	710.445	597.361	***498.293***
P	480.319	423.297	310.213	197.129	98.061	807.498	***694.414***	***595.346***
I	593.403	536.381	423.297	310.213	***211.145***	114.092	807.498	***708.430***
V	692.471	635.450	522.366	409.281	***310.213***	213.160	100.076	807.498
D	807.498	750.477	637.392	524.308	***425.240***	328.187	215.103	116.035

Supplemental Figure 35. Q-TOF LC-MS data for PLP-33 in *Rudbeckia hirta*. (**A**) Extracted ion chromatogram showing acquisition time of the peptide, peptide sequence with expected and observed mass-to-charge ratios (m/z) and peptide mass spectrum. (**B**) Tandem mass spectrum of the fragmented precursor ion. Immonium ions are denoted by their one-letter residue code. (**C**) Predicted b-ions following ring cleavage. Columns are for each cleavage point. Rows show the mass of the b-ion which contains the residue on the left at its C-terminus. Ions identified in the mass spectrum (either directly, or with loss of CO) are shown in ***bold italics***.

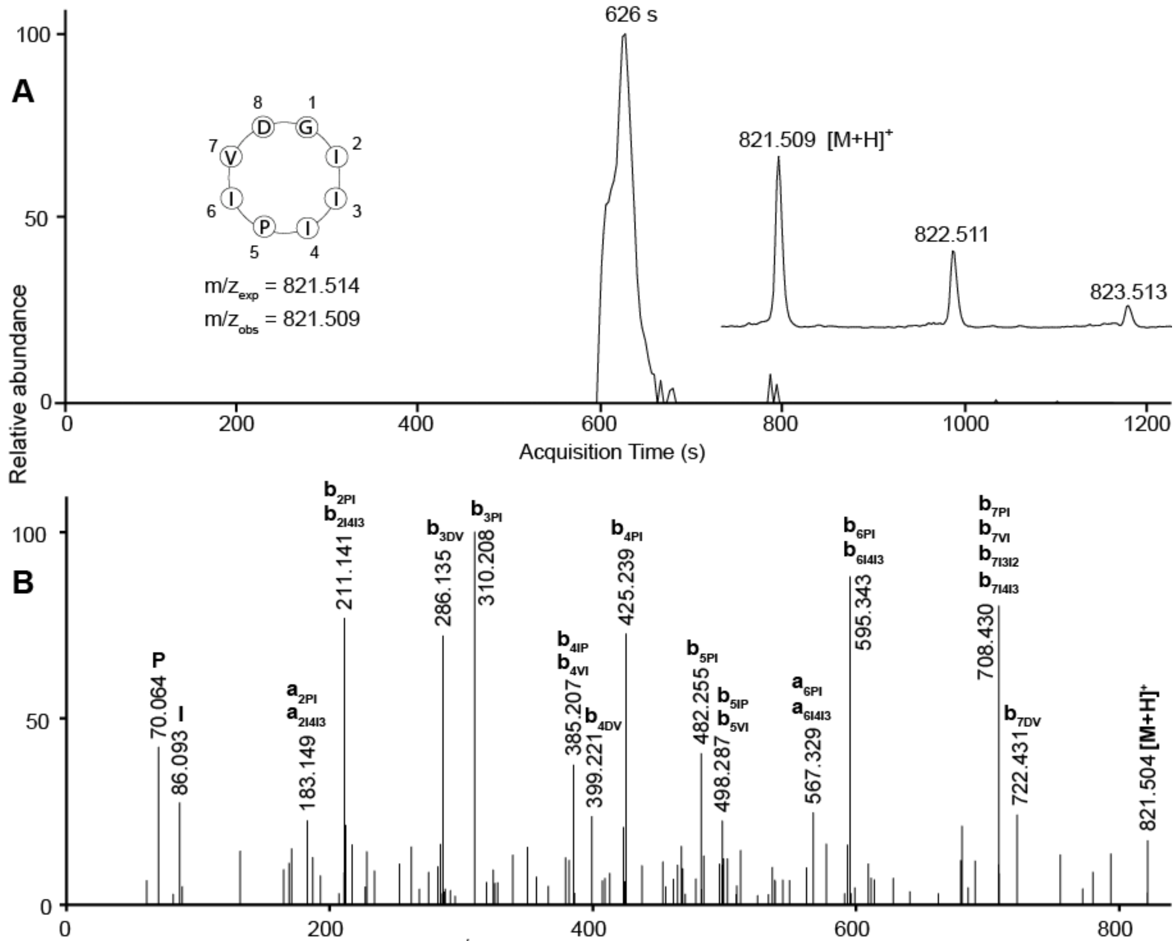

C

	G-D	I-G	I^3-I^2	I^4-I^3	P-I	I-P	V-I	D-V
G	58.029	821.514	***708.430***	***595.346***	***482.261***	***385.209***	272.125	173.056
I	171.113	114.092	821.514	***708.430***	***595.346***	***498.293***	***385.209***	***286.140***
I	284.197	227.176	114.092	821.514	***708.430***	611.377	***498.293***	***399.224***
I	397.281	340.260	227.176	114.092	***821.514***	724.461	611.377	512.308
P	494.334	437.313	324.229	211.145	98.061	821.514	***708.430***	609.361
I	607.418	550.397	437.313	324.229	***211.145***	114.092	821.514	***722.445***
V	706.487	649.465	536.381	423.297	***310.213***	213.160	100.076	821.514
D	821.514	764.492	651.408	538.324	***425.240***	328.187	215.103	116.035

Supplemental Figure 36. Q-TOF LC-MS data for PLP-34 in *Rudbeckia hirta*. (**A**) Extracted ion chromatogram showing acquisition time of the peptide, peptide sequence with expected and observed mass-to-charge ratios (m/z) and peptide mass spectrum. (**B**) Tandem mass spectrum of the fragmented precursor ion. Immonium ions are denoted by their one-letter residue code. (**C**) Predicted b-ions following ring cleavage. Columns are for each cleavage point. Rows show the mass of the b-ion which contains the residue on the left at its C-terminus. Ions identified in the mass spectrum (either directly, or with loss of H_2O or CO) are shown in ***bold italics***.

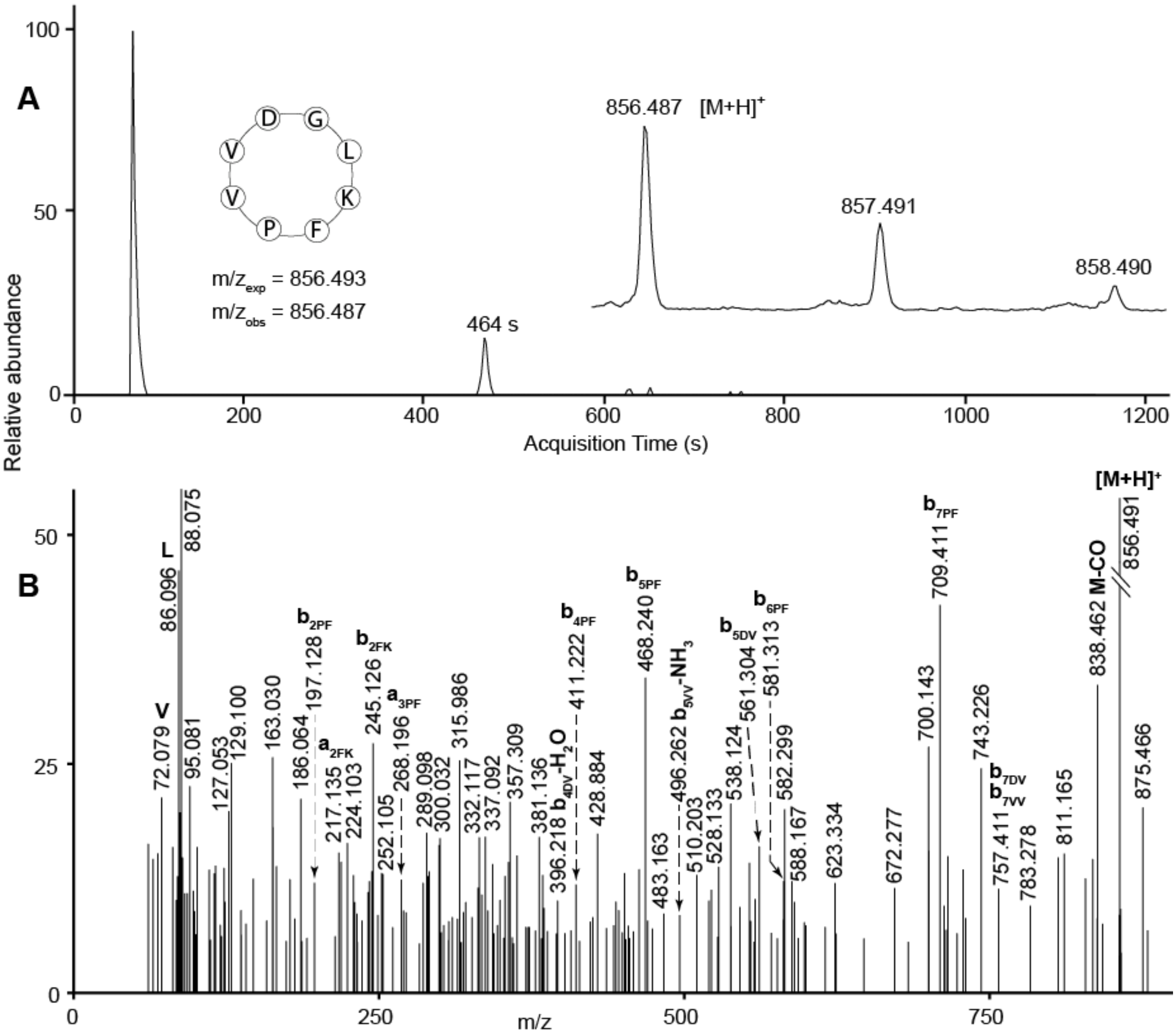

C

	G-D	L-G	K-L	F-K	P-F	V-P	V-V	D-V
G	58.029	856.493	743.409	615.314	***468.246***	371.193	272.125	173.056
L	171.113	114.092	856.493	728.398	***581.330***	484.277	385.209	286.140
K	299.208	242.187	129.103	856.493	***709.425***	612.372	***513.304***	***414.235***
F	446.277	389.255	276.171	148.076	***856.493***	759.440	660.372	***561.304***
P	543.329	486.308	373.224	***245.129***	98.061	856.493	***757.425***	658.356
V	642.398	585.376	472.292	344.197	***197.129***	100.076	856.493	***757.425***
V	741.466	684.445	571.361	443.266	***296.197***	199.145	100.076	856.493
D	856.493	799.472	686.388	558.293	***411.224***	314.172	215.103	116.035

Supplemental Figure 37. Q-TOF LC-MS data for PLP-35 in *Rudbeckia hirta*. (**A**) Extracted ion chromatogram showing acquisition time of the peptide, peptide sequence with expected and observed mass-to-charge ratios (m/z) and peptide mass spectrum. (**B**) Tandem mass spectrum of the fragmented precursor ion. Immonium ions are denoted by their one-letter residue code. (**C**) Predicted b-ions following ring cleavage. Columns are for each cleavage point. Rows show the mass of the b-ion which contains the residue on the left at its C-terminus. Ions identified in the mass spectrum (either directly, or with loss of H_2O, NH_3 or CO) are shown in ***bold italics***.

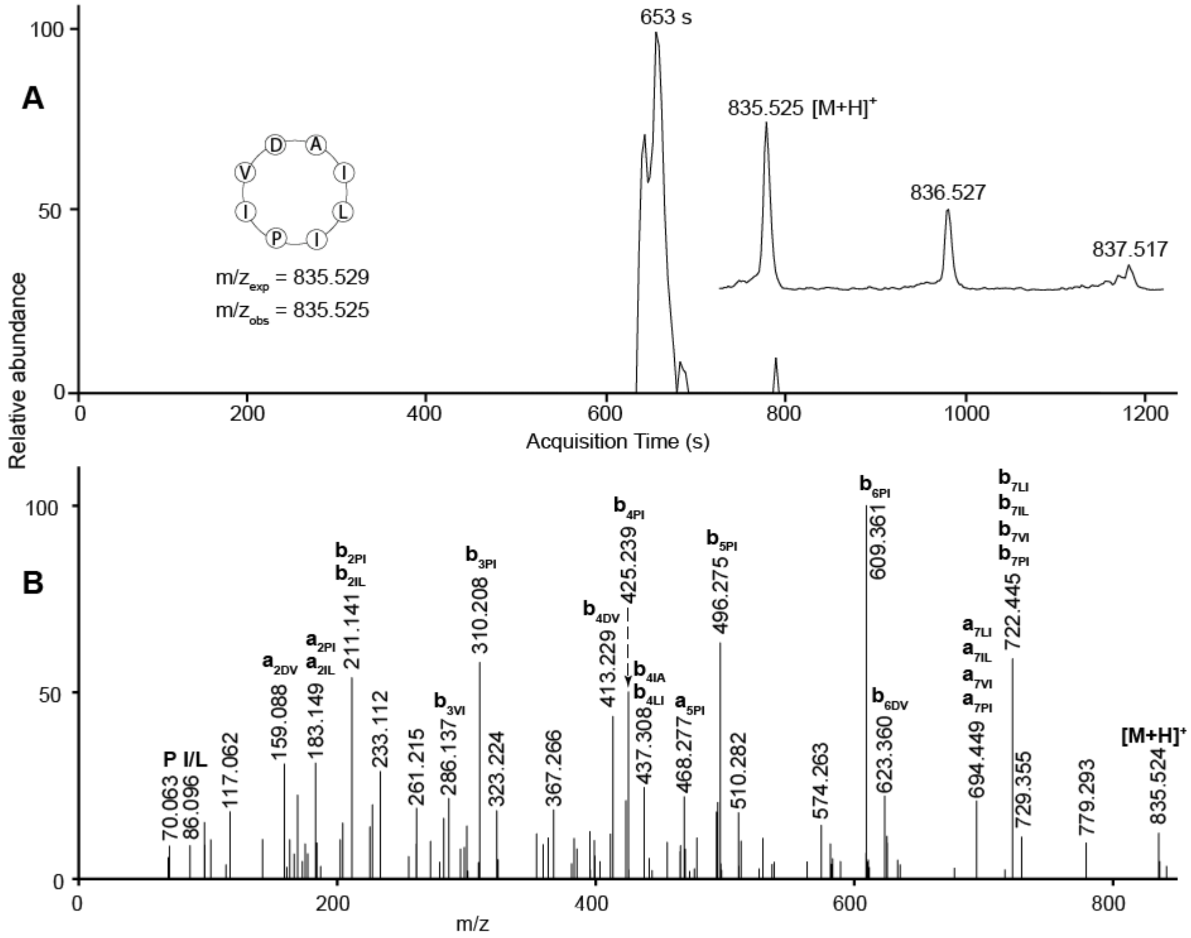

C	A-D	I-A	L-I	I-L	P-I	I-P	V-I	D-V
A	72.045	835.529	***722.445***	609.361	***496.277***	399.224	***286.140***	***187.072***
I	185.129	114.092	835.529	***722.445***	***609.361***	512.308	399.224	300.156
L	298.213	227.176	114.092	835.529	***722.445***	625.392	512.308	***413.240***
I	411.297	340.260	227.176	114.092	***835.529***	738.477	625.392	526.324
P	508.350	***437.313***	324.229	***211.145***	98.061	835.529	***722.445***	***623.377***
I	621.434	550.397	***437.313***	324.229	***211.145***	114.092	835.529	736.461
V	720.502	649.465	536.381	423.297	***310.213***	213.160	100.076	835.529
D	835.529	764.492	651.408	538.324	***425.240***	328.187	215.103	116.035

Supplemental Figure 38. Q-TOF LC-MS data for PLP-36 in *Rudbeckia hirta*. (**A**) Extracted ion chromatogram showing acquisition time of the peptide, peptide sequence with expected and observed mass-to-charge ratios (m/z) and peptide mass spectrum. (**B**) Tandem mass spectrum of the fragmented precursor ion. Immonium ions are denoted by their one-letter residue code. (**C**) Predicted b-ions following ring cleavage. Columns are for each cleavage point. Rows show the mass of the b-ion which contains the residue on the left at its C-terminus. Ions identified in the mass spectrum (either directly, or with loss of CO) are shown in ***bold italics***.

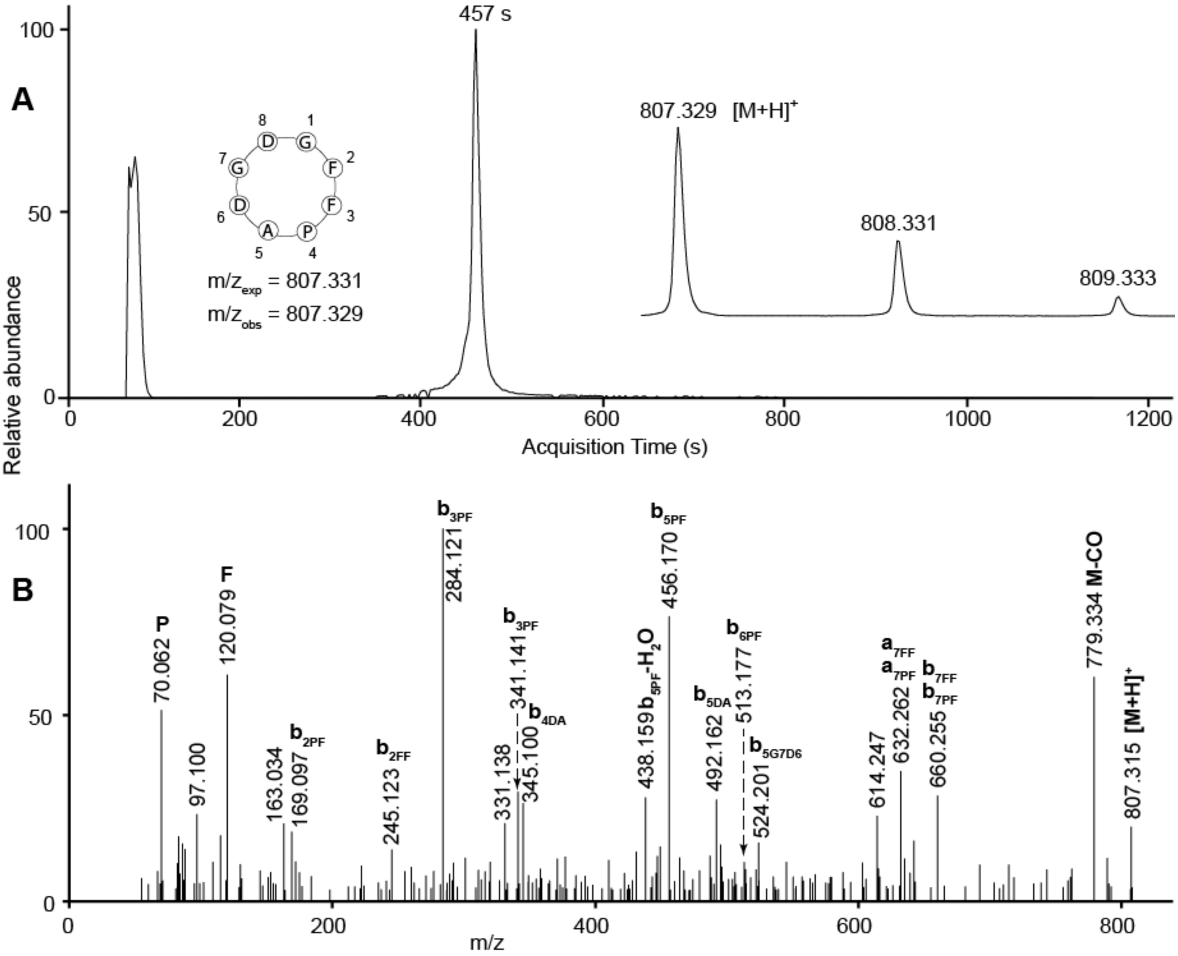

C	G^1-D^8	F-G	F-F	P-F	A-P	D-A	G^7-D^6	D-G
G	58.029	807.331	***660.263***	***513.194***	416.142	***345.105***	230.078	173.056
F	205.098	148.076	807.331	***660.263***	563.210	***492.173***	377.146	320.125
F	352.166	295.145	148.076	***807.331***	710.279	639.241	***524.215***	467.193
P	449.219	392.197	***245.129***	98.061	807.331	736.294	621.267	564.246
A	520.256	463.235	316.166	***169.098***	72.045	807.331	692.304	635.283
D	635.283	578.261	431.193	***284.125***	187.072	116.035	807.331	750.310
G	692.304	635.283	488.215	***341.146***	244.093	173.056	58.029	807.331
D	807.331	750.310	603.241	***456.173***	359.120	288.083	173.056	116.035

Supplemental Figure 39. Q-TOF LC-MS data for PLP-37 in *Rudbeckia hirta*. (**A**) Extracted ion chromatogram showing acquisition time of the peptide, peptide sequence with expected and observed mass-to-charge ratios (m/z) and peptide mass spectrum. (**B**) Tandem mass spectrum of the fragmented precursor ion. Immonium ions are denoted by their one-letter residue code. (**C**) Predicted b-ions following ring cleavage. Columns are for each cleavage point. Rows show the mass of the b-ion which contains the residue on the left at its C-terminus. Ions identified in the mass spectrum (either directly, or with loss of H_2O or CO) are shown in ***bold italics***.

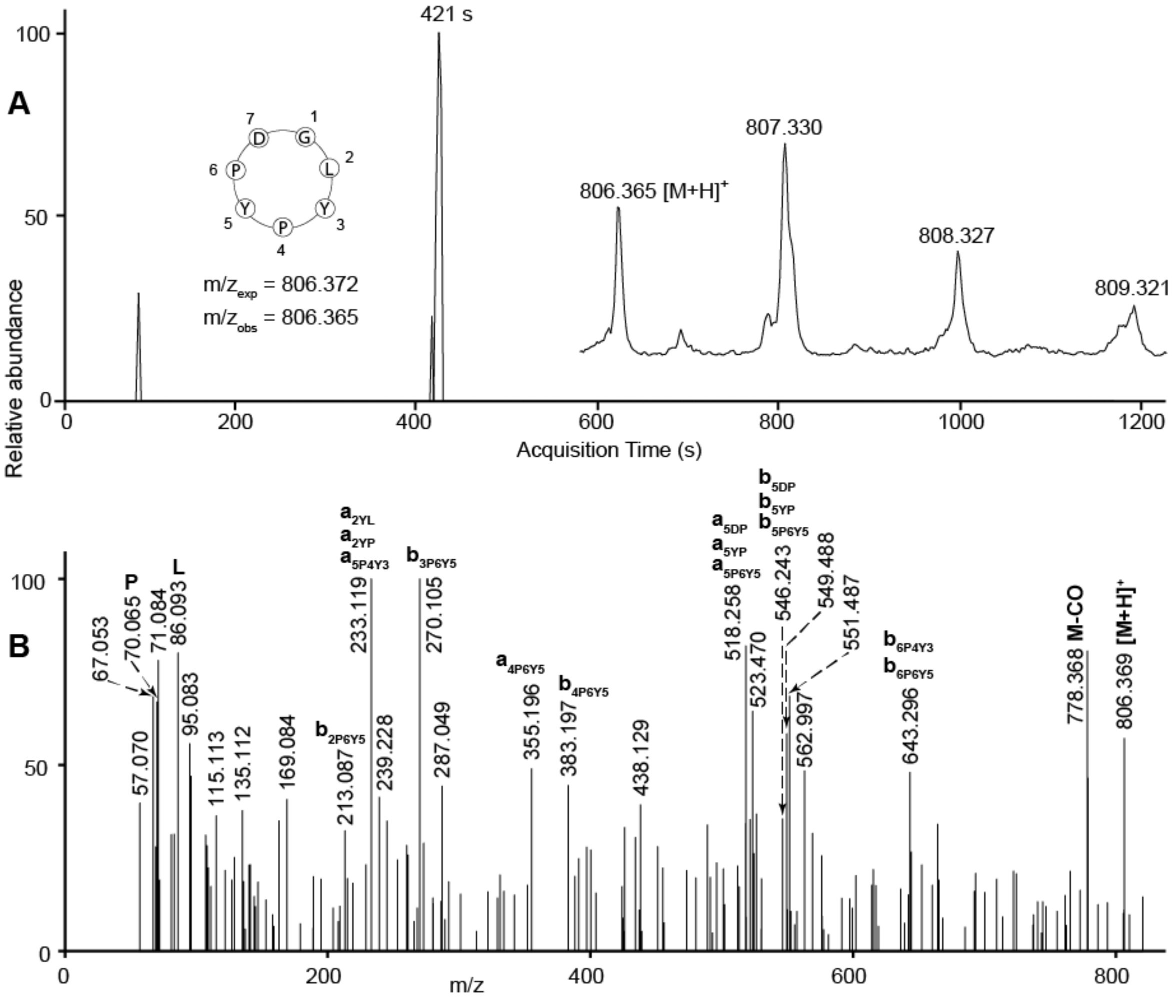

C

	G-D	L-G	Y-L	P^4-Y^3	Y-P	P^6-Y^5	D-P
G	58.029	806.372	693.288	530.225	433.172	***270.109***	173.056
L	171.113	114.092	806.372	643.309	***546.256***	***383.193***	286.140
Y	334.177	277.155	164.071	806.372	709.320	***546.256***	449.204
P	431.229	374.208	***261.124***	98.061	806.372	***643.309***	***546.256***
Y	594.293	537.271	424.187	***261.124***	164.071	***806.372***	709.320
P	691.346	634.324	521.240	358.177	***261.124***	98.061	806.372
D	806.372	749.351	636.267	473.204	376.151	***213.088***	116.035

Supplemental Figure 40. Q-TOF LC-MS data for PLP-38 in *Rudbeckia hirta*. (**A**) Extracted ion chromatogram showing acquisition time of the peptide, peptide sequence with expected and observed mass-to-charge ratios (m/z) and peptide mass spectrum. (**B**) Tandem mass spectrum of the fragmented precursor ion. Immonium ions are denoted by their one-letter residue code. (**C**) Predicted b-ions following ring cleavage. Columns are for each cleavage point. Rows show the mass of the b-ion which contains the residue on the left at its C-terminus. Ions identified in the mass spectrum (either directly, or with loss of CO) are shown in ***bold italics***.

N.B. The peptide mass spectrum is contaminated by the presence of PLP-37 at m/z 807.331 (**Supplemental Figure 39**), which is present in greater abundance than PLP-38.

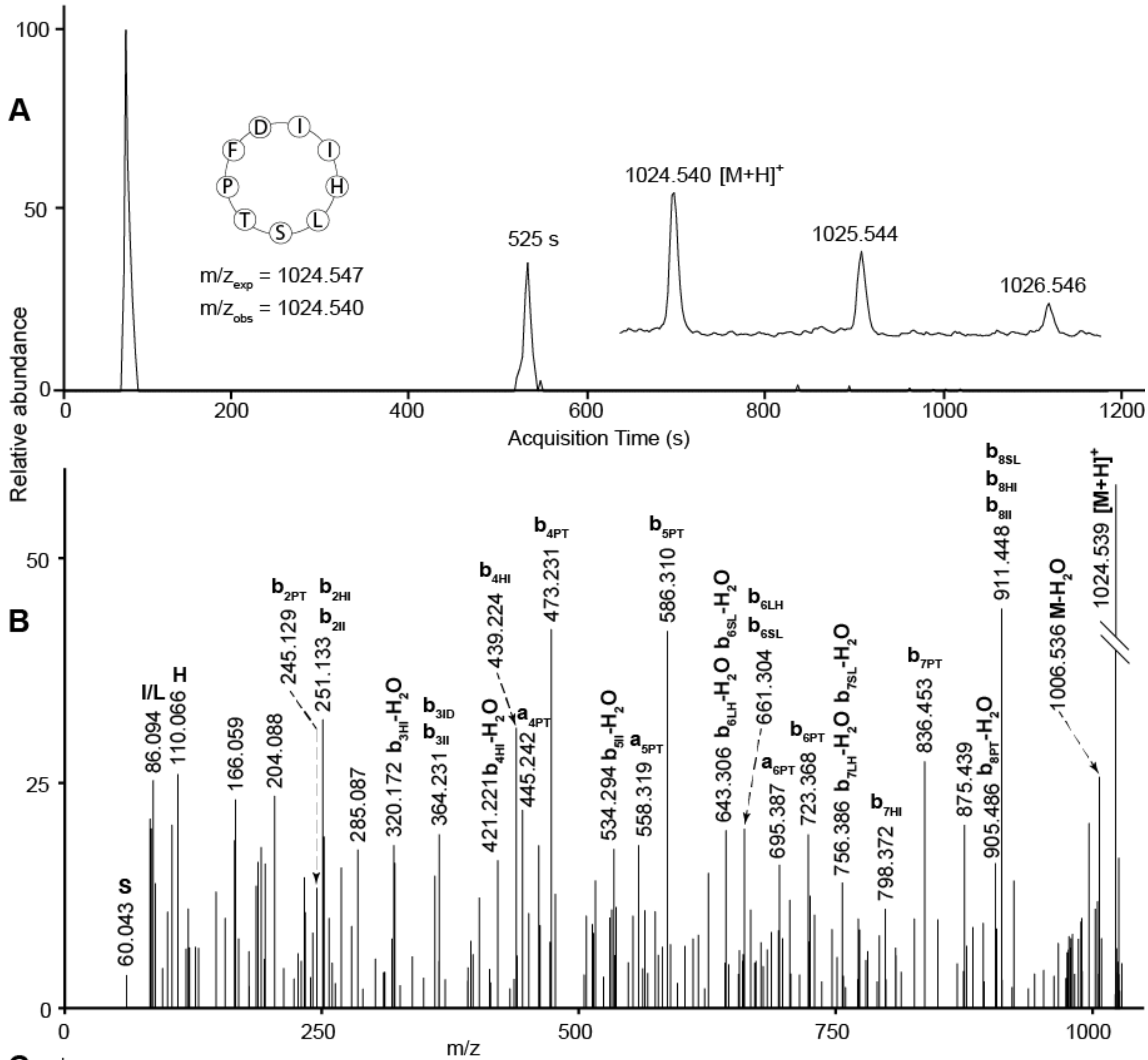

C	I-D	I-I	H-I	L-H	S-L	T-S	P-T	F-P	D-F
I	114.092	1024.547	***911.463***	***774.404***	***661.320***	574.288	***473.240***	376.187	229.119
I	227.176	114.092	1024.547	887.488	***774.404***	687.372	***586.324***	489.271	342.203
H	***364.235***	***251.151***	138.067	1024.547	***911.463***	824.431	***723.383***	626.330	479.262
L	477.319	***364.235***	***251.151***	114.092	1024.547	937.515	***836.467***	739.414	592.346
S	564.351	451.267	338.183	201.124	88.040	1024.547	***923.499***	826.446	679.378
T	665.399	***552.315***	***439.231***	302.172	189.088	102.056	***1024.547***	927.494	780.426
P	762.451	649.367	536.283	399.224	286.140	199.108	98.061	1024.547	877.478
F	909.520	796.436	683.352	546.293	433.209	346.177	***245.129***	148.076	1024.547
D	1024.547	***911.463***	***798.379***	***661.320***	548.236	461.204	360.156	263.103	116.035

Supplemental Figure 41. Q-TOF LC-MS data for PLP-39 in *Rudbeckia hirta*. (**A**) Extracted ion chromatogram showing acquisition time of the peptide, peptide sequence with expected and observed mass-to-charge ratios (m/z) and peptide mass spectrum. (**B**) Tandem mass spectrum of the fragmented precursor ion. Immonium ions are denoted by their one-letter residue code. (**C**) Predicted b-ions following ring cleavage. Columns are for each cleavage point. Rows show the mass of the b-ion which contains the residue on the left at its C-terminus. Ions identified in the mass spectrum (either directly, or with loss of H_2O or CO) are shown in ***bold italics***.

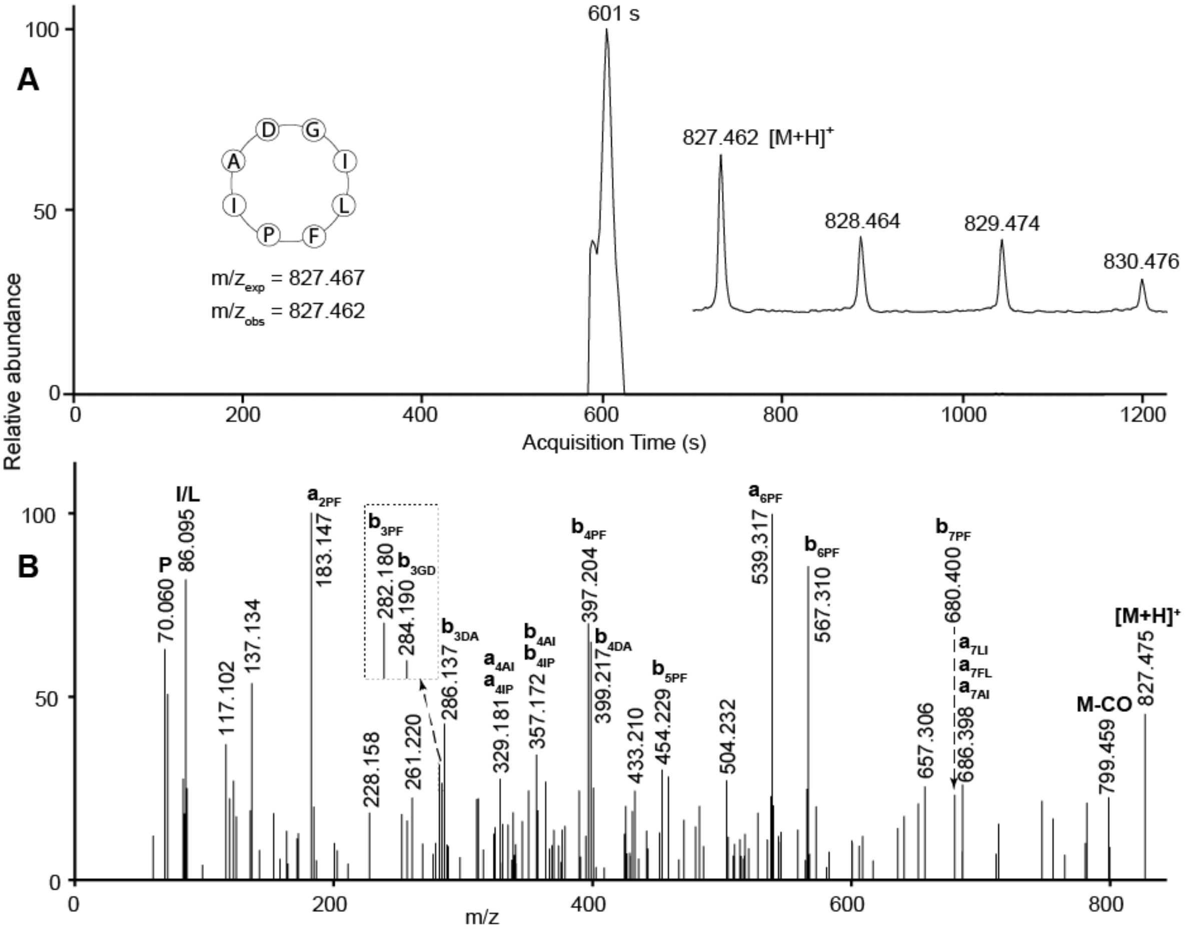

C

	G-D	I-G	L-I	F-L	P-F	I-P	A-I	D-A
G	58.029	827.467	***714.383***	601.299	***454.230***	***357.177***	244.093	173.056
I	171.113	114.092	827.467	***714.383***	***567.314***	470.261	***357.177***	***286.140***
L	***284.197***	227.176	114.092	827.467	***680.398***	583.346	470.261	***399.224***
F	431.266	374.244	261.160	148.076	***827.467***	730.414	617.330	546.293
P	528.319	471.297	358.213	245.129	98.061	827.467	***714.383***	643.346
I	641.403	584.381	471.297	358.213	***211.145***	114.092	827.467	756.430
A	712.440	655.418	542.334	429.250	***282.182***	185.129	72.045	827.467
D	827.467	770.445	657.361	544.277	***397.209***	300.156	187.072	116.035

Supplemental Figure 42. Q-TOF LC-MS data for PLP-40 in *Rudbeckia hirta*. (**A**) Extracted ion chromatogram showing acquisition time of the peptide, peptide sequence with expected and observed mass-to-charge ratios (m/z) and peptide mass spectrum. (**B**) Tandem mass spectrum of the fragmented precursor ion. Immonium ions are denoted by their one-letter residue code. (**C**) Predicted b-ions following ring cleavage. Columns are for each cleavage point. Rows show the mass of the b-ion which contains the residue on the left at its C-terminus. Ions identified in the mass spectrum (either directly, or with loss of H_2O or CO) are shown in ***bold italics***.

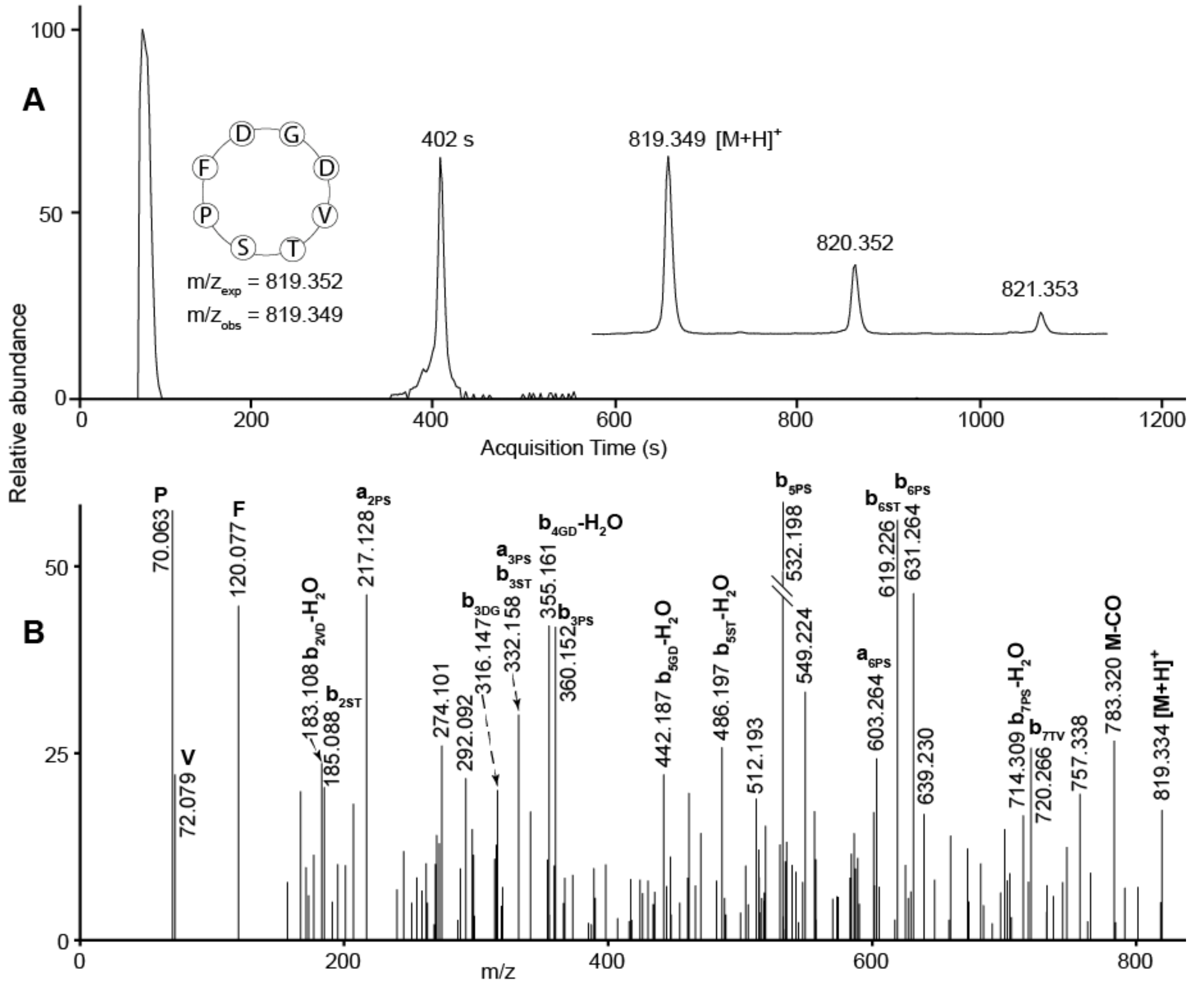

C

	G-D	D-G	V-D	T-V	S-T	P-S	F-P	D-F
G	58.029	819.352	704.326	605.257	***504.209***	417.177	320.125	173.056
D	173.056	116.035	819.352	***720.284***	***619.236***	***532.204***	435.152	288.083
V	272.125	215.103	100.076	819.352	718.305	***631.273***	534.220	387.152
T	***373.172***	***316.151***	***201.124***	102.056	819.352	***732.320***	635.268	488.199
S	***460.204***	403.183	288.156	189.088	88.040	***819.352***	722.300	575.231
P	557.257	500.236	385.209	286.140	***185.093***	98.061	819.352	672.284
F	704.326	647.304	532.277	433.209	***332.161***	***245.129***	148.076	819.352
D	819.352	762.331	647.304	548.236	447.188	***360.156***	263.103	116.035

Supplemental Figure 43. Q-TOF LC-MS data for PLP-41 in *Sanvitalia procumbens*. (**A**) Extracted ion 1chromatogram showing acquisition time of the peptide, peptide sequence with expected and observed mass-to-charge ratios (m/z) and peptide mass spectrum. (**B**) Tandem mass spectrum of the fragmented precursor ion. Immonium ions are denoted by their one-letter residue code. (**C**) Predicted b-ions following ring cleavage. Columns are for each cleavage point. Rows show the mass of the b-ion which contains the residue on the left at its C-terminus. Ions identified in the mass spectrum (either directly, or with loss of H_2O or CO) are shown in ***bold italics***.

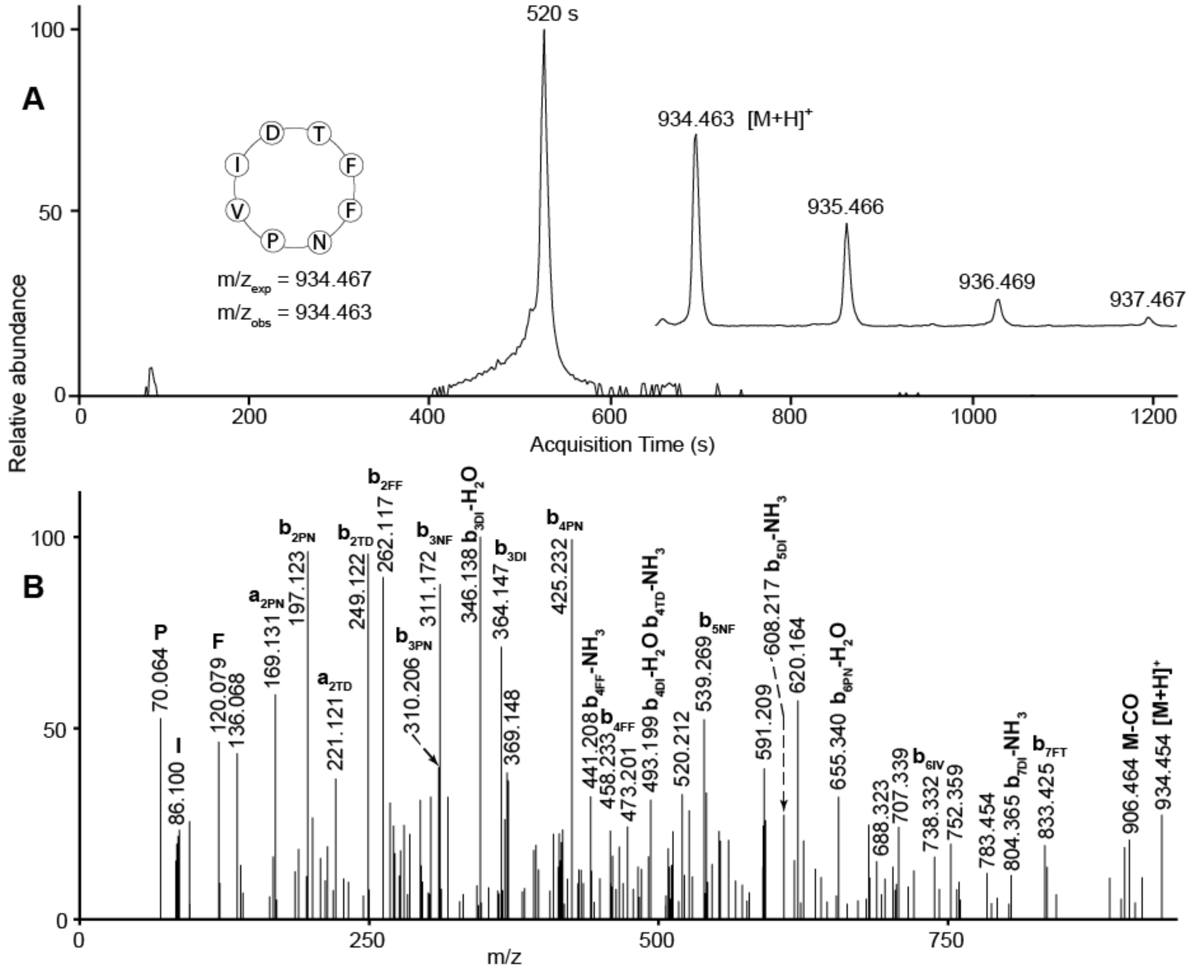

C

	T-D	F-T	F-F	N-F	P-N	V-P	I-V	D-I
T	102.056	934.467	787.399	640.331	526.288	429.235	330.167	217.082
F	***249.124***	148.076	934.467	787.399	***673.356***	576.303	477.235	***364.151***
F	396.192	295.145	148.076	934.467	820.424	723.372	624.303	***511.219***
N	***510.235***	409.188	***262.119***	115.051	934.467	837.415	***738.346***	***625.262***
P	607.288	506.240	359.172	212.104	98.061	934.467	835.399	722.315
V	706.356	605.309	***458.240***	***311.172***	***197.129***	100.076	934.467	***821.383***
I	819.440	718.393	571.324	424.256	***310.213***	213.160	114.092	***934.467***
D	934.467	***833.420***	686.351	***539.283***	***425.240***	328.187	229.119	116.035

Supplemental Figure 44. Q-TOF LC-MS data for PLP-42 in *Sanvitalia procumbens*. (**A**) Extracted ion chromatogram showing acquisition time of the peptide, peptide sequence with expected and observed mass-to-charge ratios (m/z) and peptide mass spectrum. (**B**) Tandem mass spectrum of the fragmented precursor ion. Immonium ions are denoted by their one-letter residue code. (**C**) Predicted b-ions following ring cleavage. Columns are for each cleavage point. Rows show the mass of the b-ion which contains the residue on the left at its C-terminus. Ions identified in the mass spectrum (either directly, or with loss of H_2O, NH_3 or CO) are shown in ***bold italics***.

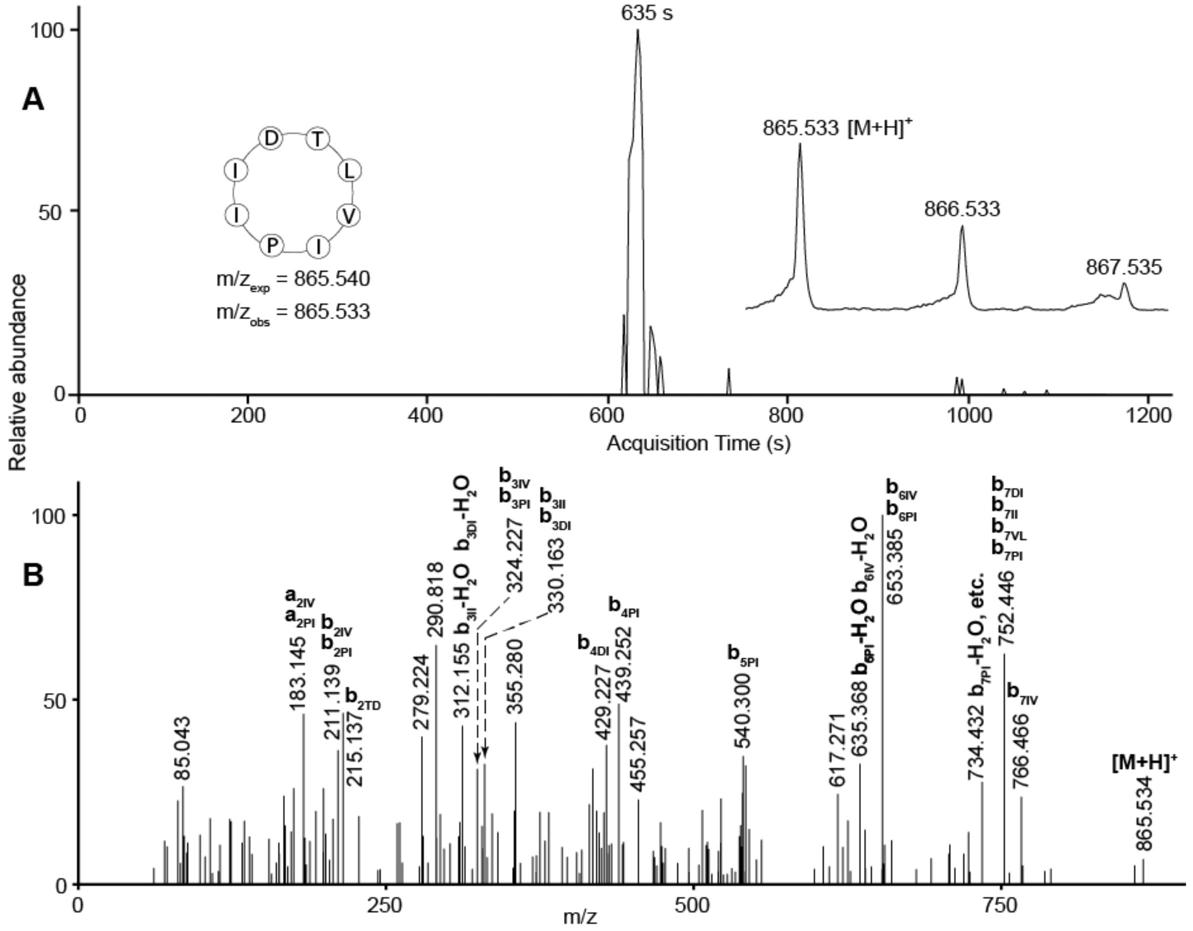

C

	T-D	L-T	V-L	I-V	P-I	I-P	I-I	D-I
T	102.056	865.540	***752.456***	***653.387***	***540.303***	443.251	***330.167***	217.082
L	***215.140***	114.092	865.540	***766.471***	***653.387***	556.335	443.251	***330.167***
V	314.208	213.160	100.076	865.540	***752.456***	655.403	542.319	***429.235***
I	427.292	326.244	213.160	114.092	***865.540***	768.487	655.403	542.319
P	524.345	423.297	310.213	***211.145***	98.061	865.540	***752.456***	639.372
I	637.429	536.381	423.297	324.229	***211.145***	114.092	865.540	***752.456***
I	750.513	649.465	536.381	437.313	***324.229***	227.176	114.092	865.540
D	865.540	764.492	651.408	552.340	***439.256***	342.203	229.119	116.035

Supplemental Figure 45. Q-TOF LC-MS data for PLP-43 in *Sanvitalia procumbens*. (**A**) Extracted ion chromatogram showing acquisition time of the peptide, peptide sequence with expected and observed mass-to-charge ratios (m/z) and peptide mass spectrum. (**B**) Tandem mass spectrum of the fragmented precursor ion. Immonium ions are denoted by their one-letter residue code. (**C**) Predicted b-ions following ring cleavage. Columns are for each cleavage point. Rows show the mass of the b-ion which contains the residue on the left at its C-terminus. Ions identified in the mass spectrum (either directly, or with loss of H_2O or CO) are shown in ***bold italics***.

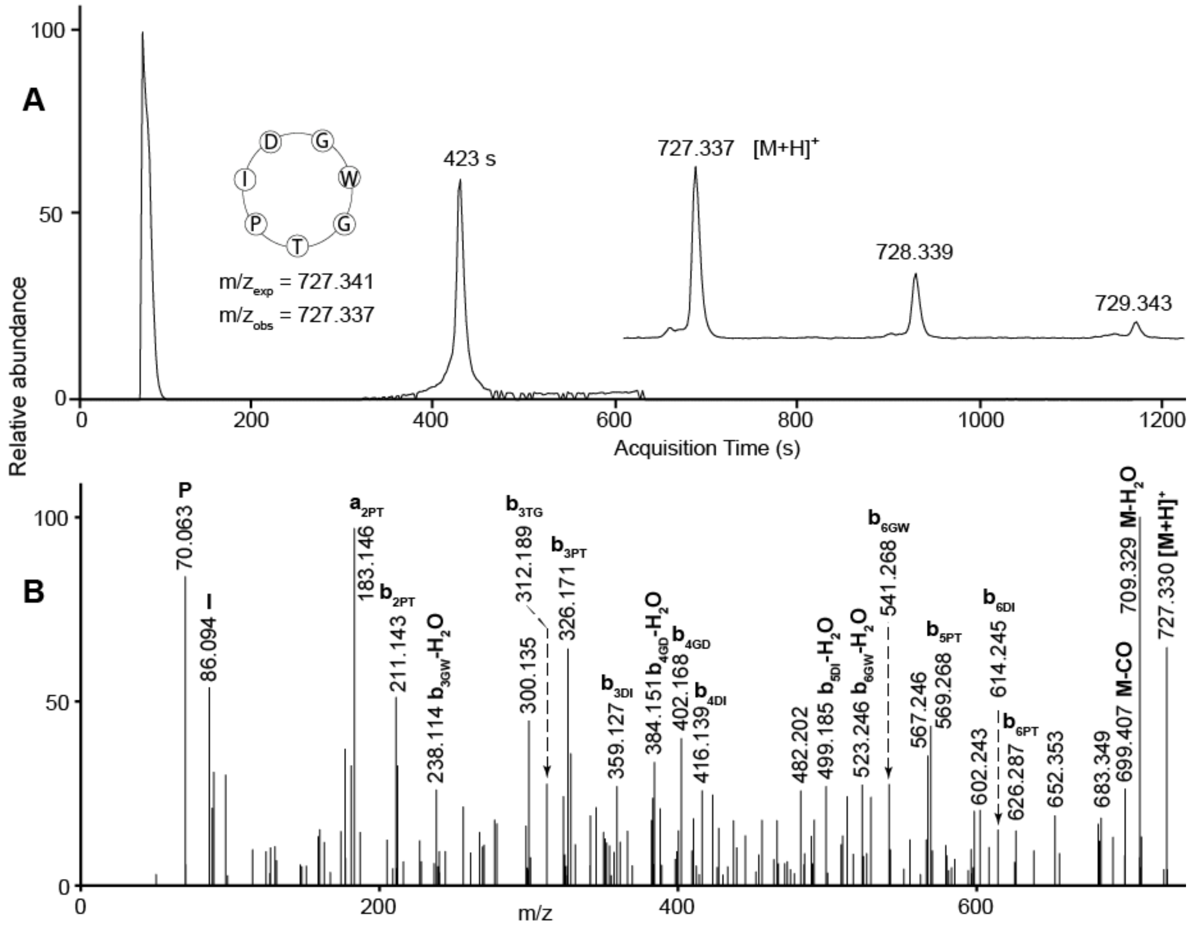

C

	G-D	W-G	G-W	T-G	P-T	I-P	D-I
G	58.029	727.341	***541.262***	484.241	383.193	286.140	173.056
W	244.109	187.087	727.341	670.320	***569.272***	472.220	***359.136***
G	301.130	244.109	58.029	727.341	***626.294***	529.241	***416.157***
T	***402.178***	345.156	159.077	102.056	***727.341***	630.289	***517.205***
P	499.230	442.209	***256.130***	199.108	98.061	727.341	***614.257***
I	612.315	555.293	369.214	***312.192***	***211.145***	114.092	727.341
D	727.341	670.320	484.241	427.219	***326.172***	229.119	116.035

Supplemental Figure 46. Q-TOF LC-MS data for PLP-44 in *Sanvitalia procumbens*. (**A**) Extracted ion chromatogram showing acquisition time of the peptide, peptide sequence with expected and observed mass-to-charge ratios (m/z) and peptide mass spectrum. (**B**) Tandem mass spectrum of the fragmented precursor ion. Immonium ions are denoted by their one-letter residue code. (**C**) Predicted b-ions following ring cleavage. Columns are for each cleavage point. Rows show the mass of the b-ion which contains the residue on the left at its C-terminus. Ions identified in the mass spectrum (either directly, or with loss of H_2O or CO) are shown in ***bold italics***.

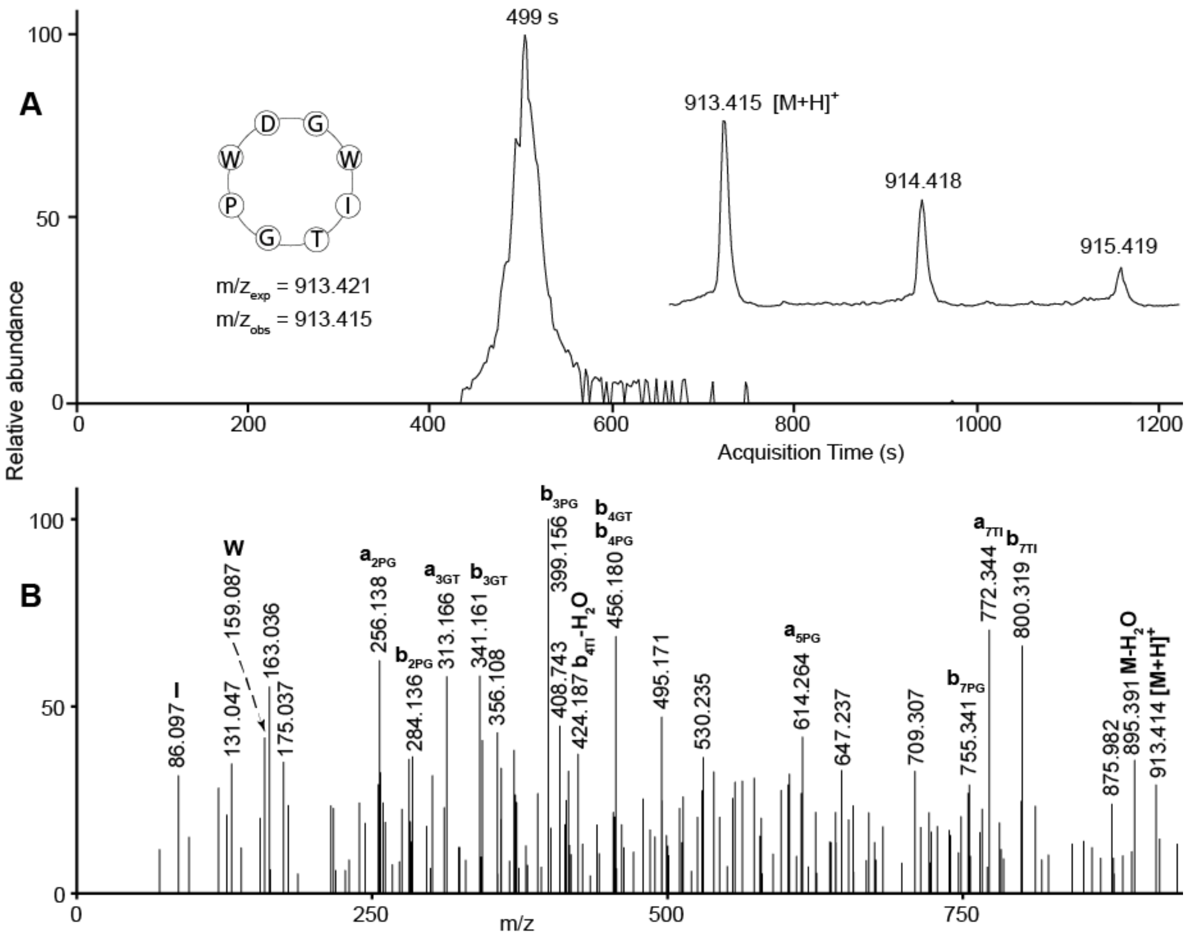

C

	G-D	W-G	I-W	T-I	G-T	P-G	W-P	D-W
G	58.029	913.421	727.341	614.257	513.210	***456.188***	359.136	173.056
W	244.109	187.087	913.421	***800.337***	699.289	***642.268***	545.215	359.136
I	357.193	300.171	114.092	913.421	812.373	***755.352***	658.299	472.220
T	458.240	401.219	215.140	102.056	913.421	856.399	759.347	573.267
G	515.262	458.240	272.161	159.077	58.029	***913.421***	816.368	630.289
P	612.315	555.293	369.214	256.130	155.082	98.061	913.421	727.341
W	798.394	741.372	555.293	***442.209***	***341.161***	***284.140***	187.087	913.421
D	913.421	856.399	670.320	557.236	***456.188***	***399.167***	302.114	116.035

Supplemental Figure 47. Q-TOF LC-MS data for PLP-45 in *Sanvitalia procumbens*. (**A**) Extracted ion chromatogram showing acquisition time of the peptide, peptide sequence with expected and observed mass-to-charge ratios (m/z) and peptide mass spectrum. (**B**) Tandem mass spectrum of the fragmented precursor ion. Immonium ions are denoted by their one-letter residue code. (**C**) Predicted b-ions following ring cleavage. Columns are for each cleavage point. Rows show the mass of the b-ion which contains the residue on the left at its C-terminus. Ions identified in the mass spectrum (either directly, or with loss of H_2O or CO) are shown in ***bold italics***.

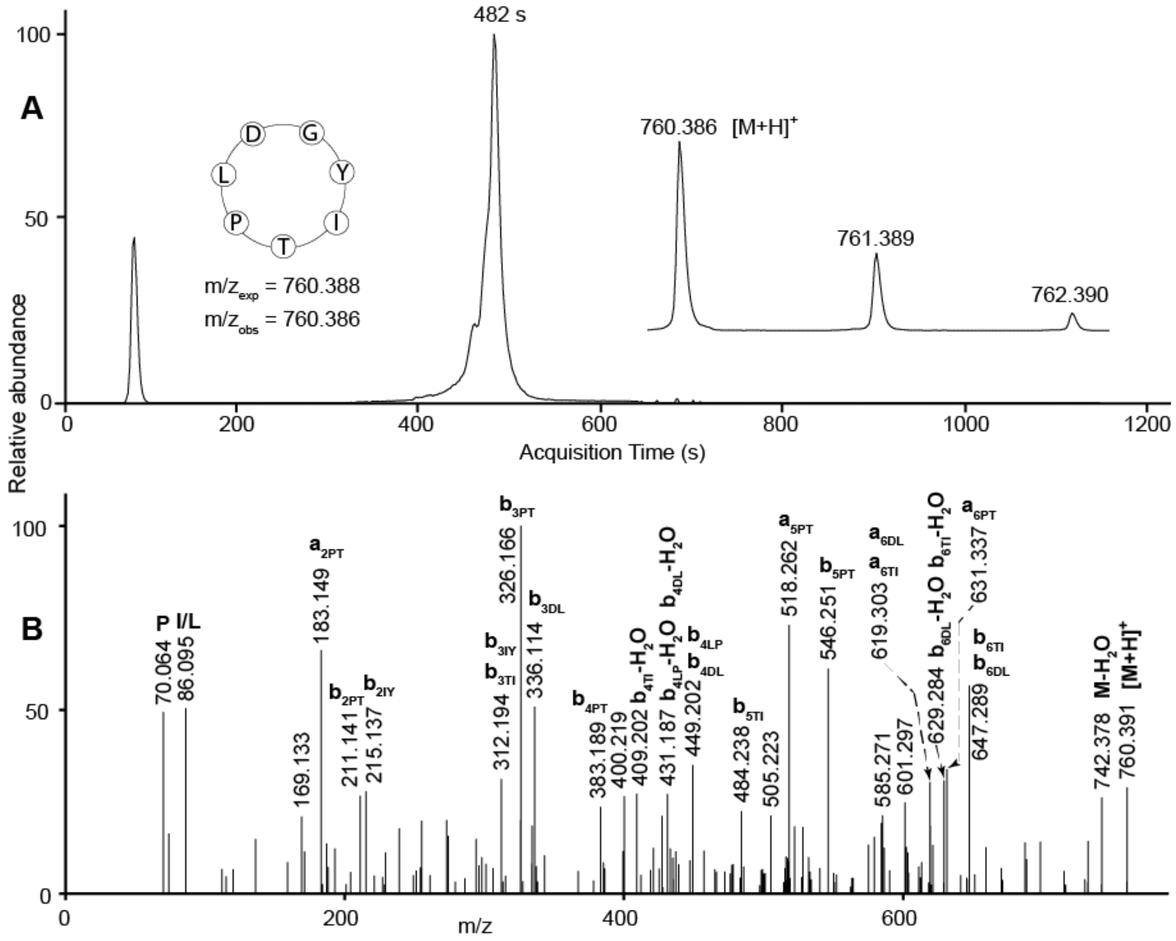

C

	G-D	Y-G	I-Y	T-I	P-T	L-P	D-L
G	58.03	760.39	597.32	***484.24***	***383.19***	286.14	173.06
Y	221.09	164.07	760.39	***647.30***	***546.26***	***449.20***	***336.12***
I	334.18	277.16	114.09	760.39	***659.34***	562.29	***449.20***
T	435.22	378.20	***215.14***	102.06	***760.39***	663.34	550.25
P	532.28	475.26	***312.19***	199.11	98.06	760.39	***647.30***
L	645.36	588.34	425.28	***312.19***	***211.14***	114.09	760.39
D	760.39	703.37	540.30	***427.22***	***326.17***	229.12	116.03

Supplemental Figure 48. Q-TOF LC-MS data for PLP-46 in *Parthenium argentatum*. (**A**) Extracted ion chromatogram showing acquisition time of the peptide, peptide sequence with expected and observed mass-to-charge ratios (m/z) and peptide mass spectrum. (**B**) Tandem mass spectrum of the fragmented precursor ion. Immonium ions are denoted by their one-letter residue code. (**C**) Predicted b-ions following ring cleavage. Columns are for each cleavage point. Rows show the mass of the b-ion which contains the residue on the left at its C-terminus. Ions identified in the mass spectrum (either directly, or with loss of H_2O or CO) are shown in ***bold italics***.

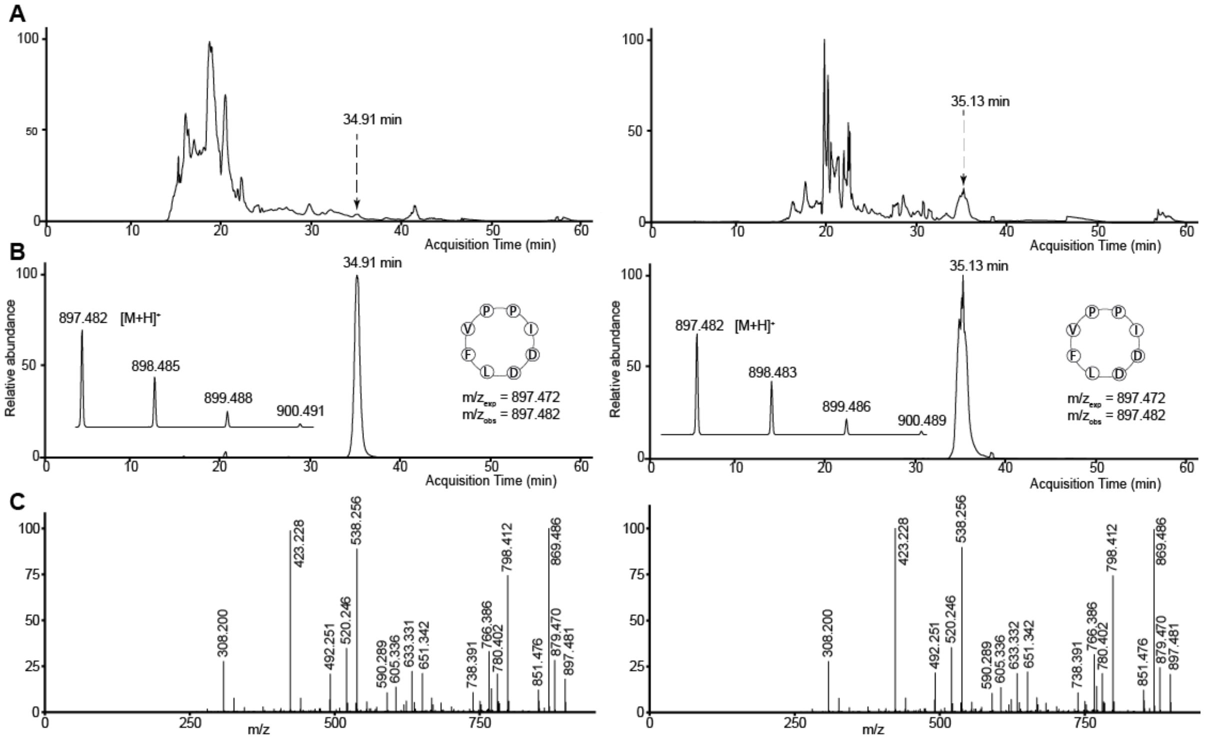

Supplemental Figure 49. Comparison of Orbitrap LC-MS data for native and synthetic PLP-2. Left, native peptide, right synthetic peptide. (**A**) Total ion current chromatogram. (**B**) Extracted ion chromatogram showing acquisition time of the peptide, with (inset right) peptide sequence with expected and observed mass-to-charge ratios (m/z) and (inset left) peptide mass spectrum. (**C**) Tandem mass spectrum of the fragmented precursor ion.

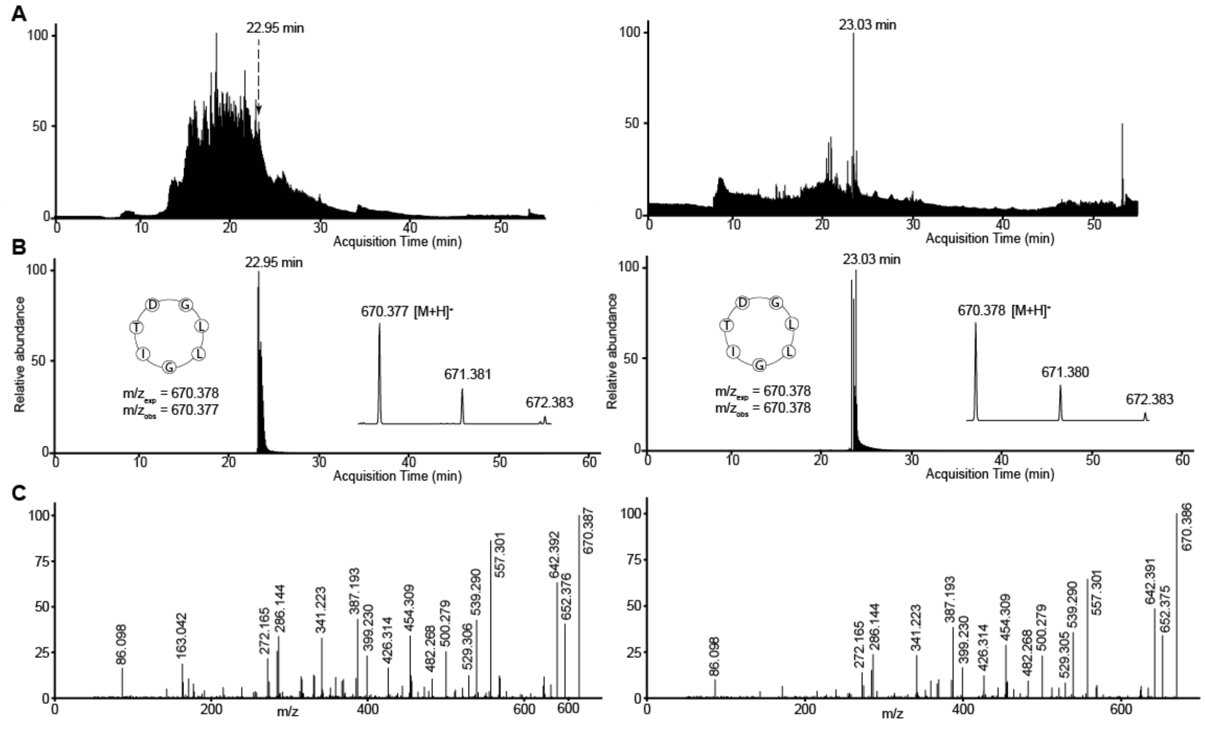

Supplemental Figure 50. Comparison of Orbitrap LC-MS data for native and synthetic PLP-4. Left, native peptide, right synthetic peptide. (**A**) Total ion current chromatogram. (**B**) Extracted ion chromatogram showing acquisition time of the peptide, with (inset left) peptide sequence with expected and observed mass-to-charge ratios (m/z) and (inset right) peptide mass spectrum. (**C**) Tandem mass spectrum of the fragmented precursor ion.

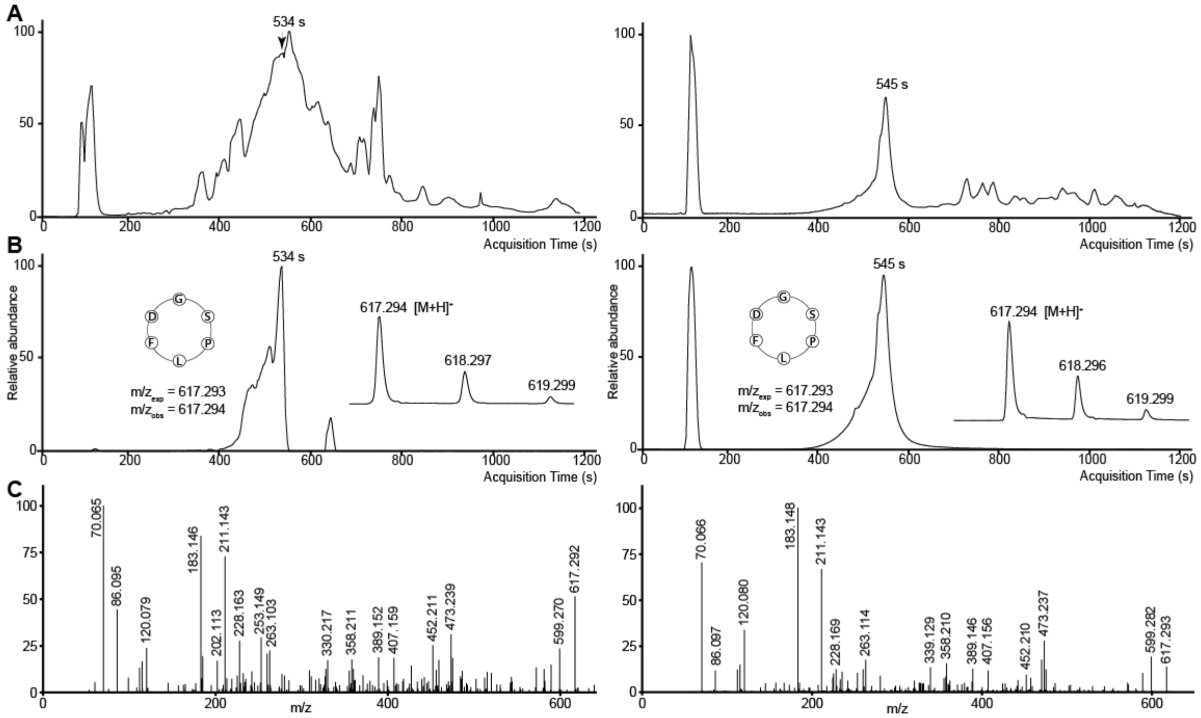

Supplemental Figure 51. Comparison of Q-TOF LC-MS data for native and synthetic PLP-10. Left, native peptide, right synthetic peptide. (**A**) Total ion current chromatogram. (**B**) Extracted ion chromatogram showing acquisition time of the peptide, with (inset left) peptide sequence with expected and observed mass-to-charge ratios (m/z) and (inset right) peptide mass spectrum. (**C**) Tandem mass spectrum of the fragmented precursor ion.

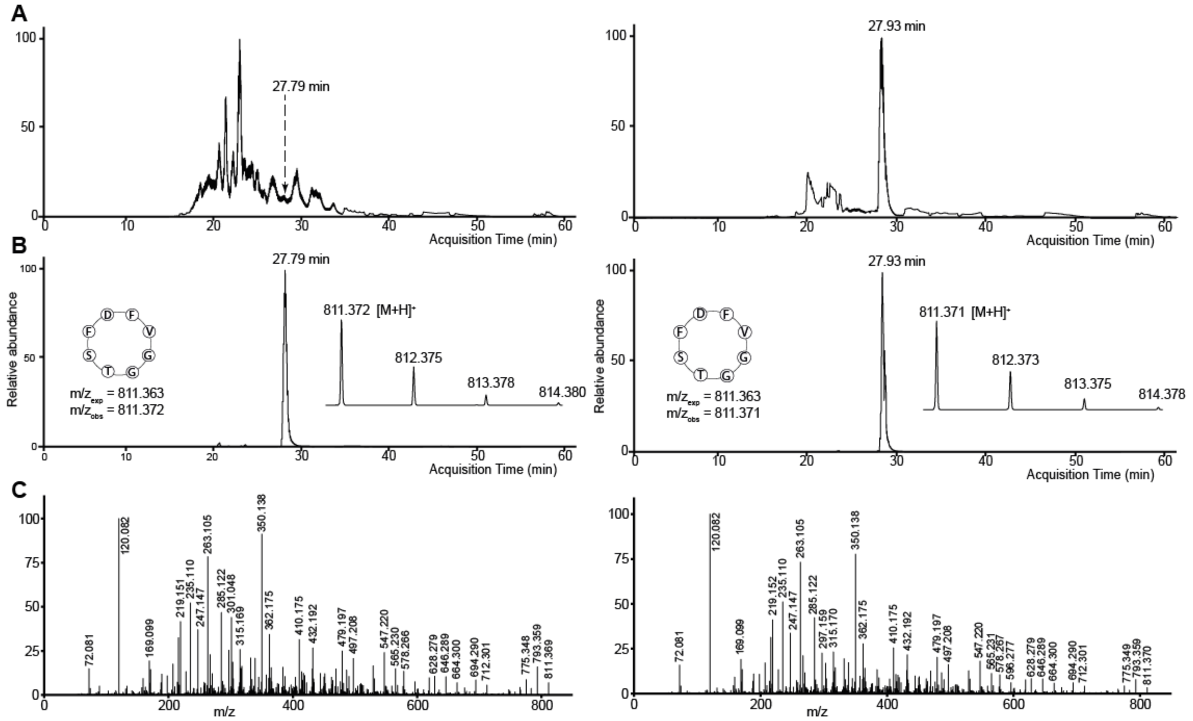

Supplemental Figure 52. Comparison of Orbitrap LC-MS data for native and synthetic PLP-12. Left, native peptide, right synthetic peptide. (**A**) Total ion current chromatogram. (**B**) Extracted ion chromatogram showing acquisition time of the peptide, with (inset left) peptide sequence with expected and observed mass-to-charge ratios (m/z) and (inset right) peptide mass spectrum. (**C**) Tandem mass spectrum of the fragmented precursor ion.

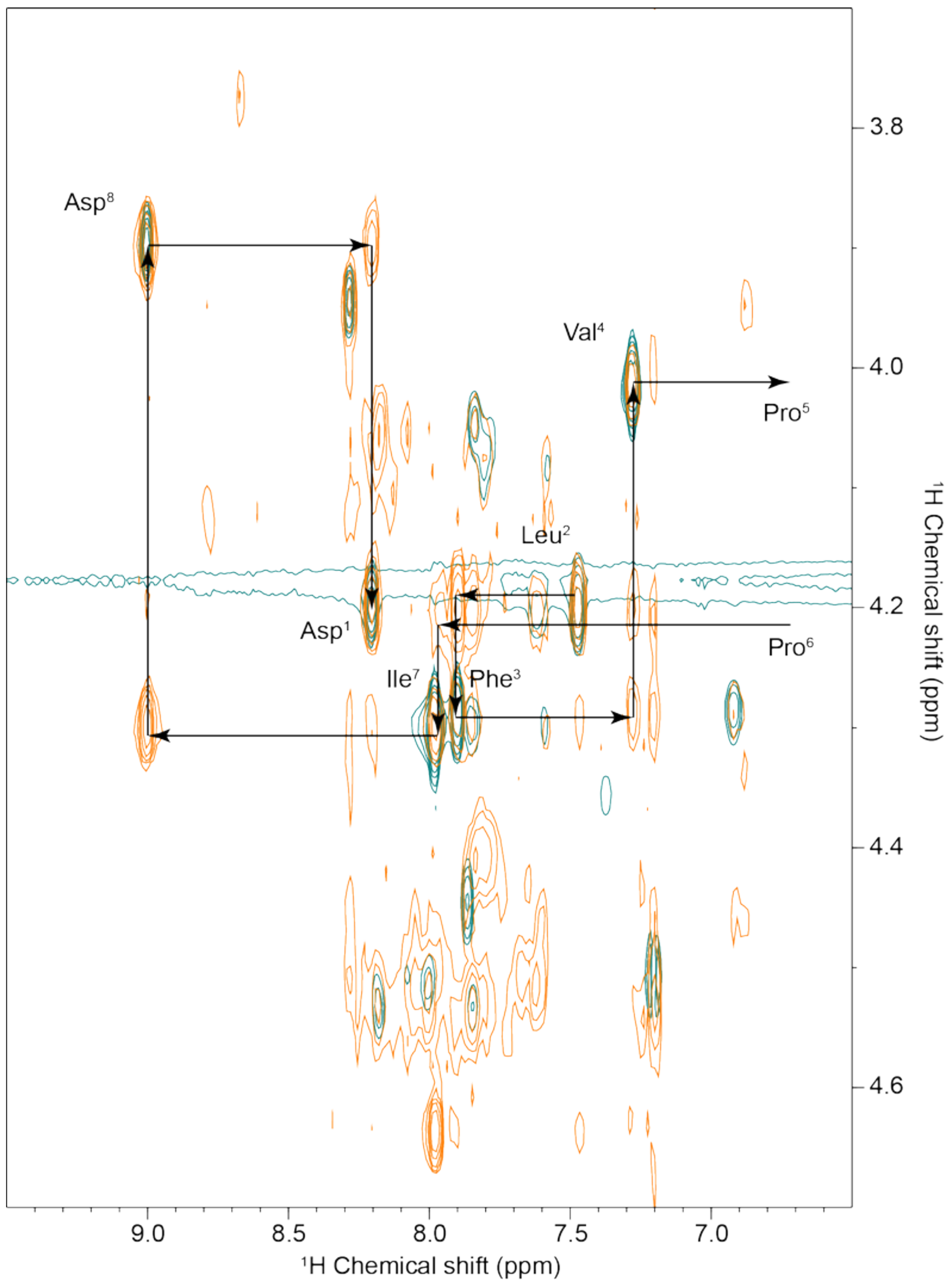

Supplemental Figure 53. Sequential walk of the fingerprint region of ^{1}H-^{1}H TOCSY (blue-green) and ^{1}H-^{1}H NOESY (orange) spectra of PLP-2 (cyclo-DLFVPPID), identifying its residue sequence.

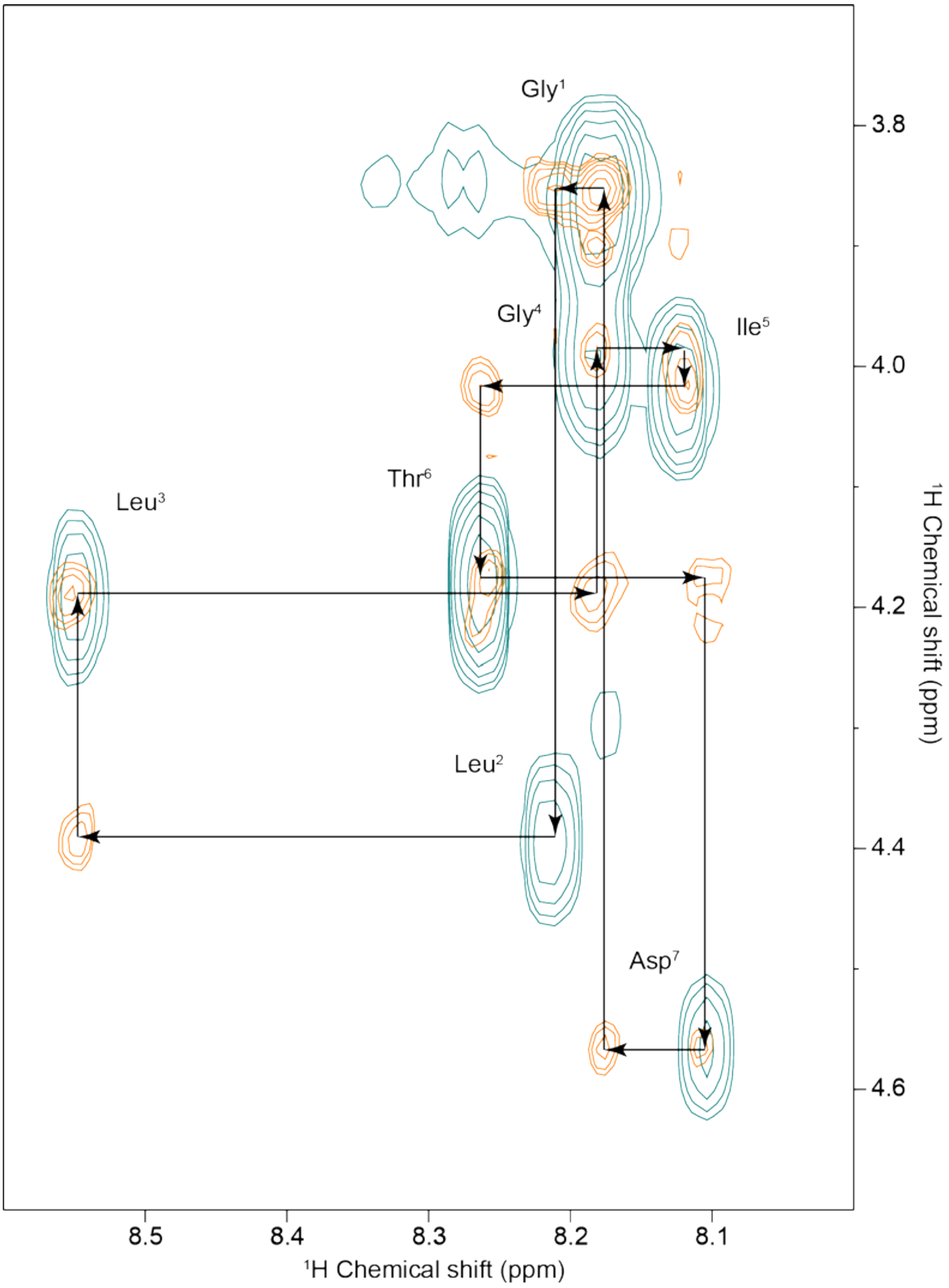

Supplemental Figure 54. Sequential walk of the fingerprint region of ^{1}H-^{1}H TOCSY (blue-green) and ^{1}H-^{1}H ROESY (orange) spectra of PLP-4 (cyclo-GLLGITD), identifying its residue sequence.

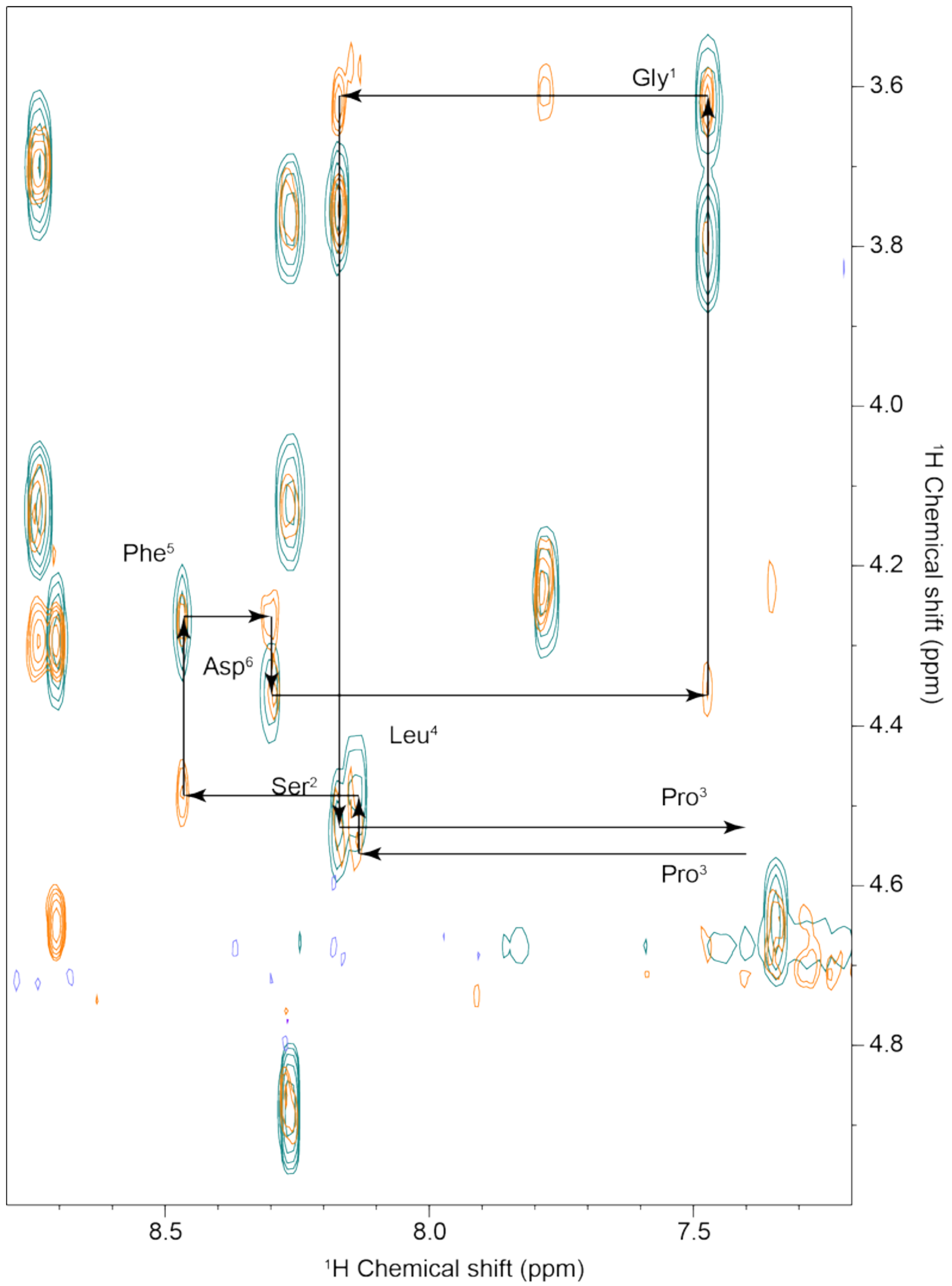

Supplemental Figure 55. Sequential walk of the fingerprint region of ^{1}H-^{1}H TOCSY (blue-green) and ^{1}H-^{1}H ROESY (orange) spectra of PLP-10 (cyclo-GSPLFD, *cis*-Pro conformation), identifying its residue sequence.

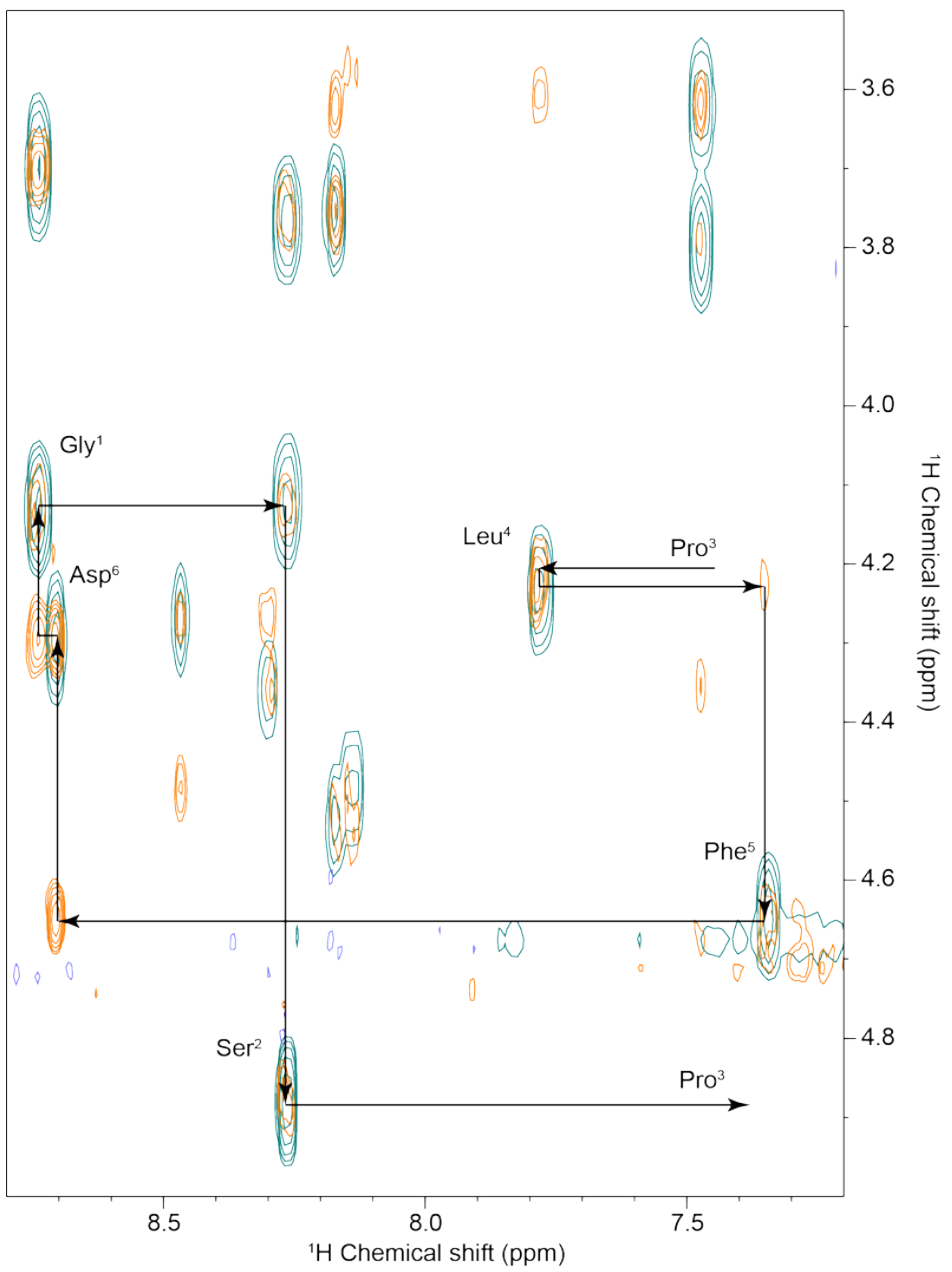

Supplemental Figure 56. Sequential walk of the fingerprint region of ^{1}H-^{1}H TOCSY (blue-green) and ^{1}H-^{1}H ROESY (orange) spectra of PLP-10 (cyclo-GSPLFD, *trans*-Pro conformation), identifying its residue sequence.

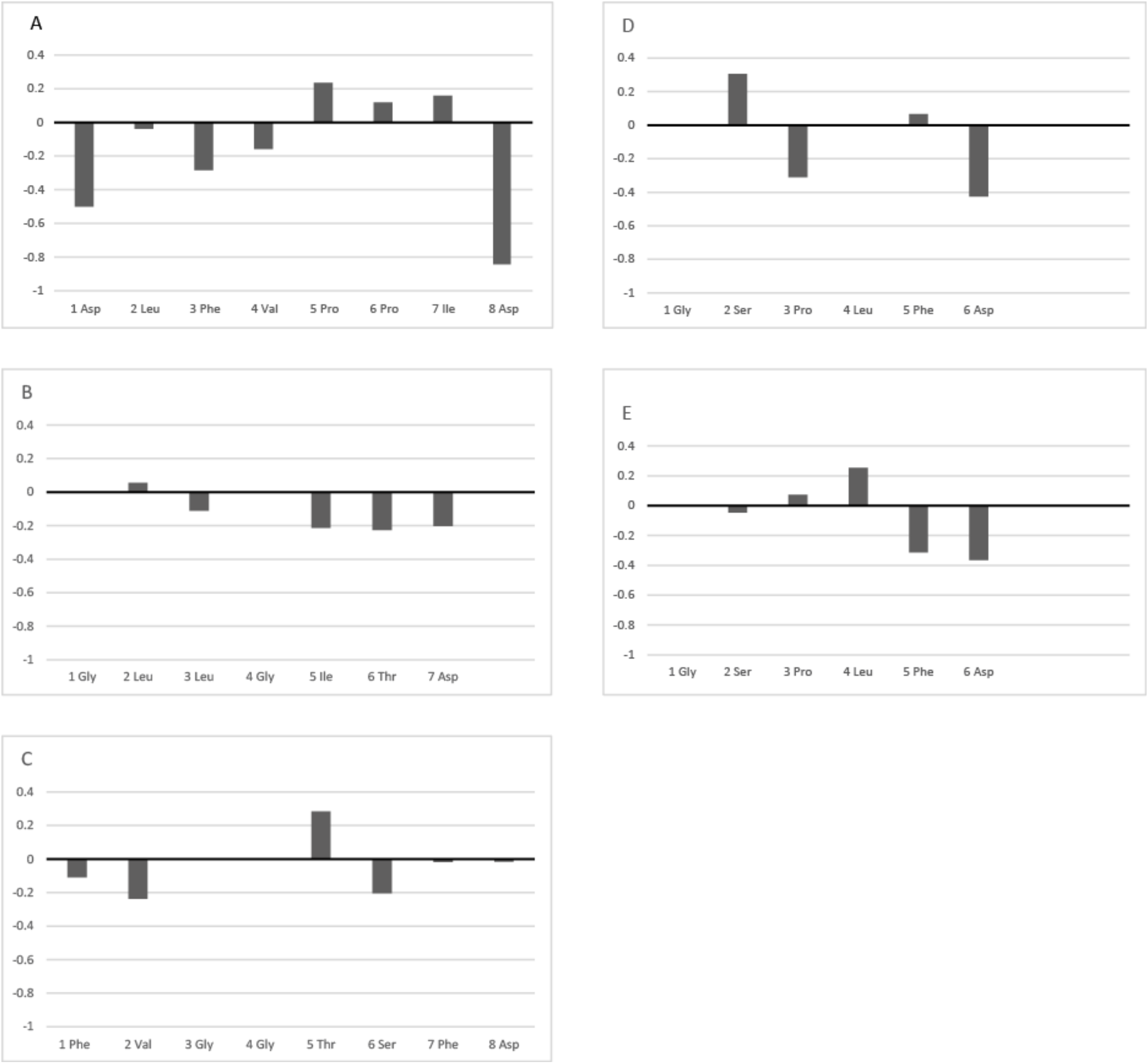

Supplemental Figure 57. Secondary H_α NMR chemical shifts for synthetic PLPs. Zero represents the random coil chemical shift value. (**A**) PLP-2; (**B**) PLP-4; (**C**) PLP-12; (**D**) PLP-10 (*trans* conformer); (**E**) PLP-10 (*cis* conformer).

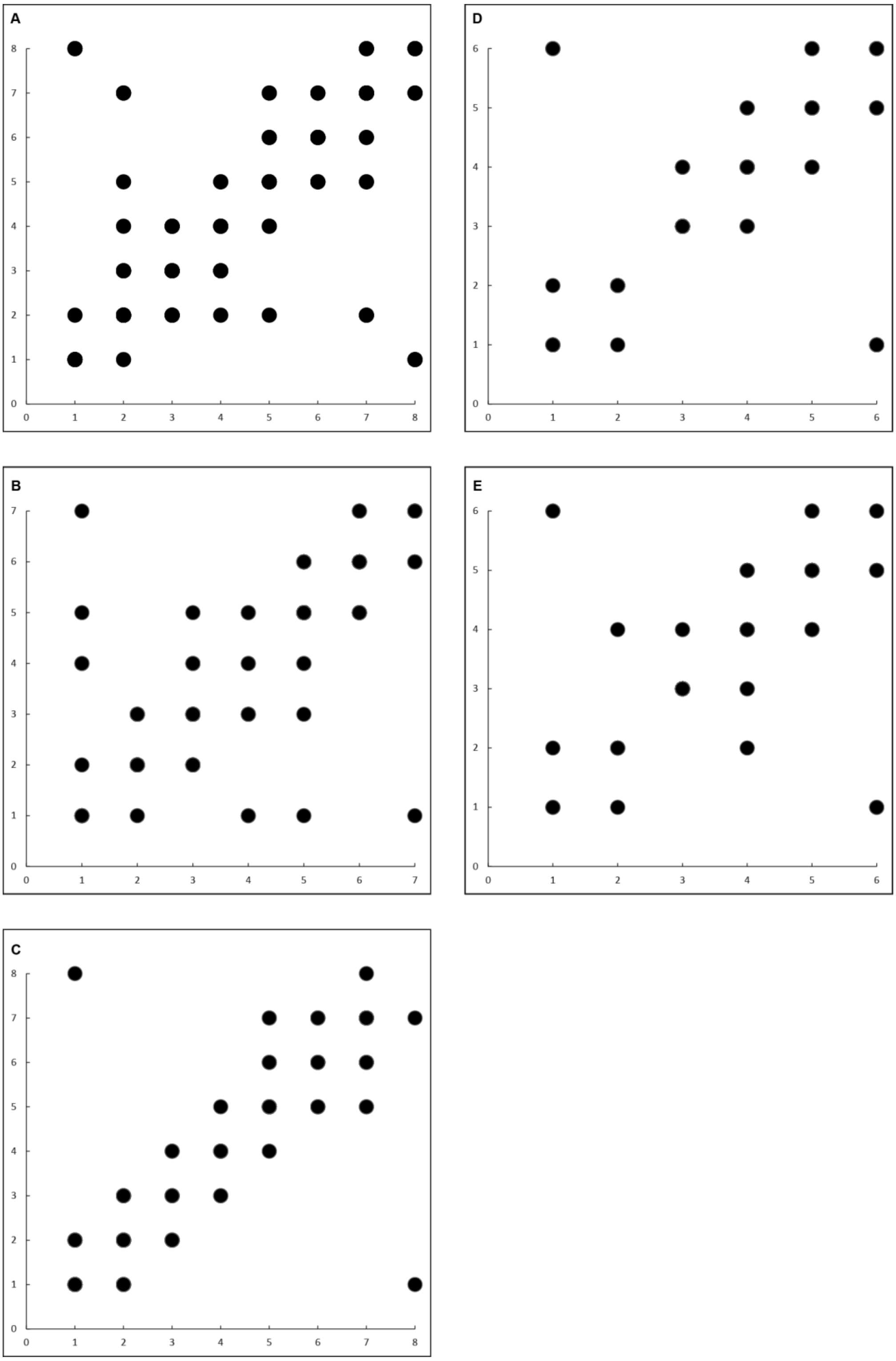

Supplemental Figure 58. NOE/ROE contact maps for synthetic PLPs. Dots are shown when there is a contact between any proton of the *i*th and *j*th amino acid residues, where *i* and *j* are plotted on the x and y axes. The diagonal represents intra-residue contacts. (**A**) PLP-2; (**B**) PLP-4; (**C**) PLP-12; (**D**) PLP-10 (*trans* conformation); (**E**) PLP-10 (*cis* conformation).

.

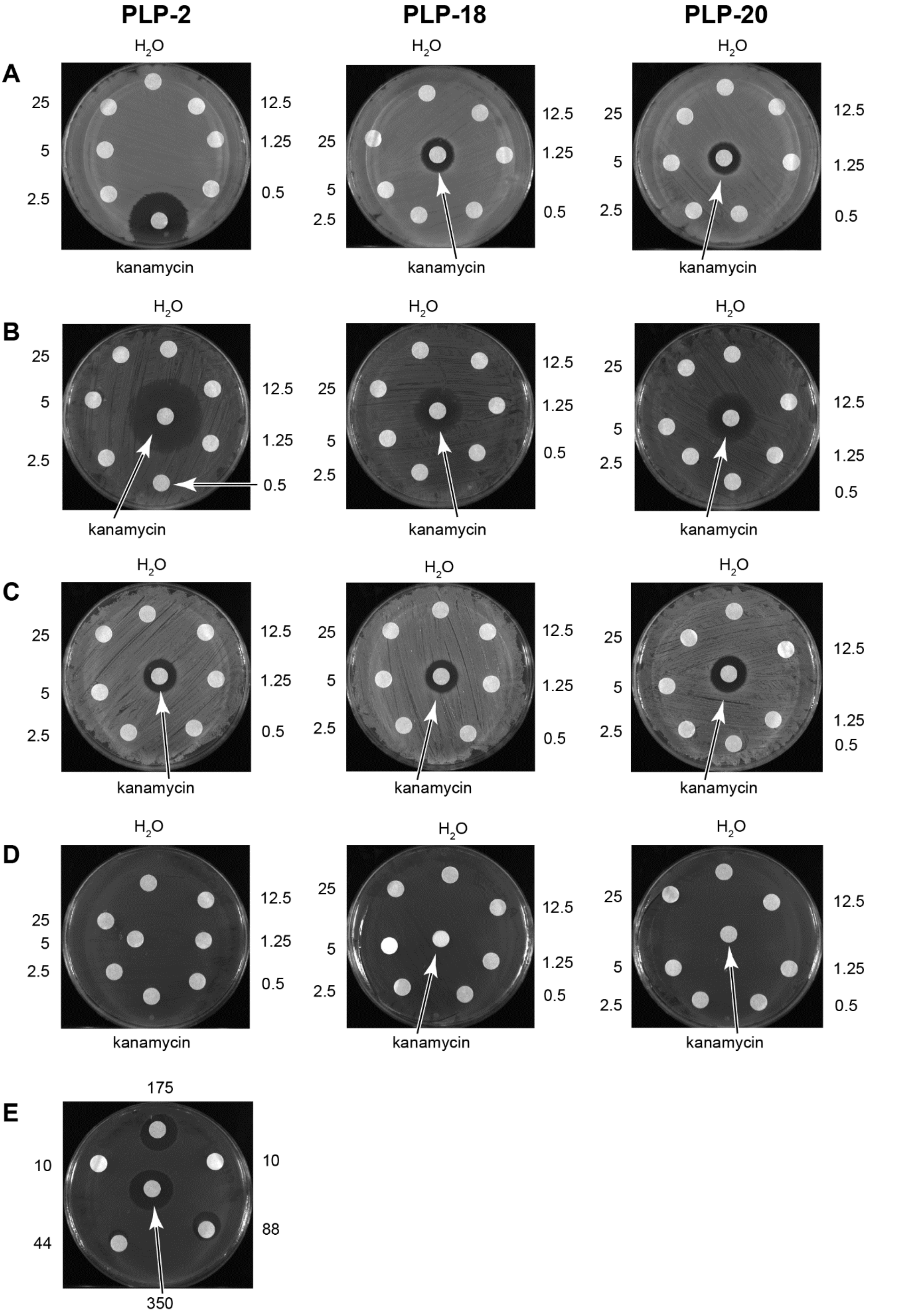

Supplemental Figure 59. Results of disc diffusion assay. (**A-D**) show bacterial plates with discs prepared with differing quantities of each peptide (from left to right, PLP-2, PLP-18 and PLP-20). Numbers represent quantites in micrograms. Discs prepared with water and 10 µg kanamycin act as negative and positive controls, respectively. (**A**) *Bacillus cereus*; (**B**) *Escherichia coli* B (**C**) *Staphylococcus aureus*; (**D**) *Pseudomonas aeruginosa*; (**E**) is a further set of controls for *P. aeruginosa* using larger quantities of kanamycin (shown in micrograms).
