# Supplementary material for: A family of small, cyclic peptides buried in preproalbumin since the Eocene epoch
Source: Plant Direct. 2018 Feb 28;2(2):e00042. doi: 10.1002/pld3.42 (PMC6223261; doi:10.1002/pld3.42)
Supplement: Supplementary file 2 [file PLD3-2-e00042-s002.pdf]

We thank the editor for asking us to make minor revisions so that the paper might be accepted for publication in *Plant Direct*.

We are grateful to all three reviewers for the time taken to provide us with their valuable comments, which have allowed us to improve our manuscript. Please find below a point-by-point response to all comments, with action taken in the manuscript highlighted in bold font. New or modified text is in italics. The full reports from both reviewers are included at the end of this document for your convenience.

We have uploaded a PDF file in which all new text in the main and supplementary is blue colour (Fisher_PLP_v22_NEW_IS_BLUE.pdf).

We look forward to your response to our revised manuscript.

POINT-BY-POINT RESPONSE TO REVIEWERS:

Reviewer #1

No changes were suggested by Reviewer #1

Reviewer #2

[COMMENT #1] "While the introduction is well-written and informative, I think it would benefit from an overview figure that summarizes the different classes of peptides the authors describe here, alongside their conserved regions and mode of processing"

[RESPONSE #1] **We have added an overview figure (Figure 1)** and new caption that summarizes the five different classes of plant cyclic peptides mentioned in the introduction and referred to it at appropriate points in the text.

[COMMENT #2] "All the PLPs identified in this study end with an Asp residue and one starts with a Lys residue. While I believe that indeed the cyclization occurs exclusively between N- and C-terminus (especially because the authors did a careful comparison between some extracted PLPs with their synthetic analogs), I wonder if the authors have any other experimental evidence that it is not the side chain of the C-terminal Asp (or in one case the N-terminal Lys) that is involved in the macrocyclization. As the C-terminal Asp is apparently universal in these peptides, this might merit further discussion by the authors."

[RESPONSE #2] The evidence we have of head-to-tail cyclisation and not side chain is that for at least 4 of the PLPs we compared retention time and MS/MS fragmentation of four native and synthetic PLPs. It is possible, but unlikely those with amino-containing side chains could be side chain cyclised by AEP (would be same mass as backbone cyclic) as this reaction has not been observed to be performed by AEP. In the Discussion section titled "Sequencing of PLPs by LC-MS/MS" we have added a paragraph to mention this possibility:

Although we matched four synthetic, head-to-tail cyclized PLPs to the native PLP in LC-MS and MS/MS, it has been assumed, based on conservation that all PLPs are head-to-tail cyclic. Some PLPs contain residues with amino groups in their side-chain that could conceivably take the place of the proto-amino terminus in the transpeptidation reaction performed by AEP. Most conspicuous of these is PLP-24, which has a Lys residue at its proto-N-terminus. PLP-6, PLP-30, and PLP-35 have internal Lys residues that, if side-chain cyclized, would form 'lasso' peptides of the same mass as backbone cyclic. With limited plant material, it was not possible to purify enough native material of these four to confirm they had the conserved, AEP-mediated backbone cyclic structure as the four native PLPs for which a synthetic version was matched (Supplemental Figure 49-52). Several native PDPs have similarly been confirmed as backbone cyclic and for SFTI-1 and SFT-L1 from sunflower, native material was used to solve their solution structures (Luckett et al., 1999; Mylne et al., 2011; Elliott et al., 2014).

[COMMENT #3] "The statement about PLPs not being antibacterial is one of the biggest issues I have with this study. The reason is that the authors only tested against a single E. coli strain, which does not justify such a general statement (with regard to the stated lack of antifungal activity, at least three strains were assessed). The authors should either revise that statement or expand their panel of tested organisms. If they want to do the latter, a variety of gram-positive and gram-negative bacteria should be used, of which some are ideally found in the same environment as the plant species from which the respective PLPs were discovered."

[RESPONSE #3] **We carried out additional antibacterial activity assays** with a wider range of synthetic peptides than before (including some we received recently) and with additional Gram-negative and Gram-positive strains. Consistent with our previous findings, the PLPs tested were not antibacterial. **These data are presented as a Supplemental Figure 59** with photographs of the results of the disc diffusion assays we carried out. **The methods have been rewritten** to show in more detail what we did:

Plates were prepared with 25 mL LB agar and incubated overnight at 37 °C to confirm their sterility. LB medium (5 mL) was inoculated with three to five single colonies from agar plates of the Gram-negative bacteria Escherichia coli B or Pseudomonas aeruginosa (ATCC 19429), or the Gram-positive bacteria Bacillus cereus (ATCC 10876) or Staphylococcus aureus (ATCC 25923), and incubated with shaking at 37 °C overnight. A 50 µL aliquot of each overnight culture was used to inoculate fresh 5 mL aliquots of LB and the cultures were incubated at 37 °C with shaking for 2.5 h. The final cultures were diluted with sterile saline to an OD_{600} of 0.1. This turbidity level was established by colony count to fall within the range of $1x10^7$ to 2×10^7 colony-forming units (CFU)/mL for the Gram-positive strains, and 5×10^7 to 7×10^7 CFU/ml for the Gram-negative strains. Standardized bacterial suspensions were seeded evenly onto sterile LB agar plates using a sterile swab. Synthetic peptides PLP-2, PLP-18 and PLP-20 were dissolved in dimethyl sulfoxide to a concentration of 5 mg/mL; an aliquot of each of these solutions was diluted with water to a concentration of 1 mg/mL. Each solution was dispensed onto sterile 8 mm diameter filter papers in the following amounts: 0.5 µg, 1.25 µg, 2.5 µg, 5 µg, 12.5 µg and 25 µg. Two controls were set up: one containing 5 µL of water, the other 10 µg of kanamycin. All the filter papers were allowed to dry completely before placing them on the inoculated LB plates previously prepared. The plates were incubated overnight at 37 °C, after which they were inspected for bacterial growth.

The **results section has been updated** also:

> Although some studies on orbitides have indicated they possess antibiotic activity, an inspection of LB agar plates after overnight incubation at 37°C showed no inhibition of the growth of *the Gram-negative bacteria, Escherichia coli B and Pseudomonas aeruginosa, nor of the Gram-positive bacteria, Bacillus cereus and Staphylococcus aureus,* by any of the six discs containing between 0.5 µg and 25 µg of synthetic *PLP-2, PLP-18 or PLP-20*, nor did a negative control disc, treated only with water, inhibit growth *(**Supplemental Figure 59A-D**).* By contrast *there was a clear zone of growth inhibition around a positive control disc containing kanamycin, except in the case of P. aeruginosa, which is known to be more resistant to kanamycin than the other bacterial species (Morita et al., 2014). However, a separate control plate with discs containing larger amounts of kanamycin did show growth inhibition of P. aeruginosa (**Supplemental Figure 59E**).*

[COMMENT #4] "The fact that some Asteroideae species produce more than one PLP might suggest that these are multicomponent antimicrobials that are only active in combination, but not on their own. This is something already observed in other RiPP classes, and while it cannot be addressed experimentally in this study, it could at least be discussed in the manuscript.

[RESPONSE #4] We have **added two sentences** to the end of the discussion section on "Anti-Bacterial and Anti-Fungal Assays" **stating**: "*As most Asteroideae species produce more than one PLP, it is possible that they could act as multicomponent antimicrobials that only work in combination, but not on their own. This behaviour has been observed in the two-component bacteriocins produced by lactic acid bacteria (Garneau et al., 2002), in which two peptides act synergistically against other Gram-positive bacteria. They can be divided into two classes: the lantibiotics which comprise two ribosomally-synthesized and heavily post-transationally modified lanthipeptides, and the nonlantibiotics, which contain few post-translational modifications other than disulfide bonds. These bacteriocins differ from the PLPs in that they are not head-to-tail cyclic and tend to be larger in size (30-60 amino acids). With limited seed material, we had to synthesize PLPs to acquire sufficient peptide to characterize them and so were unable to perform these assays with PLPs in combination.*"

[COMMENT #5] Additionally, when discussing in what tribes the authors identified PLPs, they should also highlight which of these species produced more than one PLP."

[RESPONSE #5] We have **expanded the part of the results section** to name which of these species produced more than one PLP. The previous paragraph *"A similar analysis of the LC-MS/MS data from other species of the Asteroideae (Supplemental Figures 1-48) revealed a total of 46 unique PLPs in 17 species (Table 1). PLP-26 and PLP-28 were each found in two different species, Inula helenium and Inula racemosa."*

Now reads:

> *A similar analysis of the LC-MS/MS data from other species of the Asteroideae (Supplemental Figures 1-48) revealed a total of 46 unique PLPs in 17 species (Table 1). In total, 48 PLPs were sequenced as PLP-26 and PLP-28 were each found in two different species, Inula helenium and Inula racemosa. Of the 17 species containing PLPs, most contained more than one. Only five species contained a single detectable PLP, namely Dahlia variabilis, Cosmos bipinnatus, Engelmannia peristenia, Arnica chamissonis and Parthenium argentatum. Of these five species, all except Arnica chamissonis had more than one PawL1 gene so they may well have more than one PLP, but they fall below our limit of detection. Overall, these findings suggest most species contain multiple PawL1 copies and multiple PLPs.*

[COMMENT #6] "In the introduction, the authors mention recent works that traced the evolutionary history of STFI-1 and other bicyclic peptides to orbitide ancestors. As STFI-1 is a protease inhibitor (a feature also found for other cyclic peptides and other RiPPs like e.g. the microviridins), the authors should consider adding assays where they test their four synthetic PLPs for inhibition of commercially available proteases. Such assays could be performed very quickly and, even if they fail, would be interesting for comparison to STFI-1."

[RESPONSE #6] Whilst it is certainly possible, we felt it **improbable that these peptides function as protease inhibitors** as they do not have the beta strand structure that is universally found in substrates (and thus inhibitors) of proteases [1]. Furthermore, Alexander Konarev published work showing no evidence of serine protease inhibitors in the Asteraceae (formerly Compositae) with molecular weights below 1,500 Da [2] and many of these specific species either have or (based on the age of the *PawL* gene) are likely to have *PawL1* genes. Finally, as there are many different proteases, it is hard to make any definitive statement without testing many of them against PLPs for inhibitory activity. We would point out that even among the PDPs (the peptide group to which SFTI-1 belongs) very few inhibit trypsin like SFTI-1 does [3]. So, especially due to their lack of beta strand structure found universally in proteases inhibitors, we are reluctant to carry out protease inhibition assays on PLPs as they are likely to be unsuccessful.

[1] Tyndall, Nall, and Fairlie. "Proteases universally recognize beta strands in their active sites." *Chemical Reviews* 105.3 (2005): 973-1000.

[2] Konarev et al. "Serine proteinase inhibitors in the Compositae: distribution, polymorphism and properties." *Phytochemistry* 59.3 (2002): 279-291.

[3] Elliott et al. "Evolutionary origins of a bioactive peptide buried within preproalbumin." *Plant Cell* 26.3 (2014): 981-995.

[COMMENT #7] "At the beginning of their discussion section, the authors mention that 22 of the undetected peptide sequences had no C-terminal Asp and thus were not being processed by the respective organisms. I would welcome if they could also discuss, if these peptides contain the conserved flanking regions described in Figure 4 or lack them as well."

[RESPONSE #7] To address this comment we analysed all the precursor sequences, whether they made a detectable PLP or not. We found all the precursor sequences, whether a matching PLP was detectable or not, had similar flanking sequences. With this more thorough analysis, **we added two new panels to Figure 5** and **added the following text to the results**:

> *"The flanking sequences for putative PLPs we could not detect still showed similar conservation to regions in transcripts for PLPs we could detect (Figure 5D,E)."*

[COMMENT #8] "Furthermore, the authors should comment if there is any evidence of the according preproteins being either evolutionary ancestors from which the PLP-containing preproteins were derived or if they look more like they were PLP-containing proteins at one point, but then got inactivated by random mutation of the C-terminal Asp residue."

[RESPONSE #8] We would suggest that the peptide sequences without the C-terminal Asp have been inactivated by random mutation, as their sequences often differ from typical, expressed PLPs in other ways. They often lack the N-terminal Gly and contain residues that are uncommon in PLPs, such as Ser,

His, Met, Trp and Gln. We **have added more discussion**, speculating that these 22 are derived from PLP-making *PawL1* genes, not ancestors.

The previous text:

> *"Of the undetected peptide sequences, 22 did not include a proto-C-terminal Asp, whereas this Asp was absolutely conserved in all the observed peptides; this supports the hypothesis that PLPs, like PDPs, are processed by an AEP that cleaves only when an Asp is present in the P1 position."*

Now reads:

> *"Of the undetected peptide sequences, 22 did not include a proto-C-terminal Asp, whereas this Asp was absolutely conserved in all the observed peptides; this supports the hypothesis that PLPs, like PDPs, are processed by an AEP that cleaves only when an Asp is present in the P1 position. The rarity of these sequences and their confinement within a phylogenetic span of species with PLPs lead us to hypothesize that these genes lacking a proto-C-terminal Asp were likely to have been PLP-containing proteins at one point, but have lost their PLP-coding capability by random mutation in or near the sequence encoding the proto-C-terminal Asp. Most are still open reading frames and may well still make functional seed storage albumins."*

[COMMENT #9] "In the first paragraph of the "LC-MS/MS and Peptide Sequencing" section in the Materials & Methods the authors should mention which column they used."

[RESPONSE #9] We used a "chip" from Agilent Technologies containing a trapping column and an analytical column (Large Capacity Chip (II), Part No. G4240-62010). **We have added this information to the M&M** as follows**:**

> "Briefly, a gradient elution was run with HPLC-grade solvents (Honeywell) from 5% solvent B to 95% solvent B over 15 min at a flow rate of 300 nL/min *using a C18 high capacity nano-LC chip (Part No. G4240-62010, Agilent Technologies) comprising a trapping column (160 nL) and an analytical column (75 µm x 150 mm)*"

[COMMENT #10] "The MS2 spectra containing figures are pretty packed with information to a point where they become a bit confusing. While it would be too much effort to change all of these, the authors could at least add some color to Figure 2 and 3 in the main text, to make them more accessible to the reader."

[RESPONSE #10] We did as suggested and in these two figures **we have highlighted each ion series using a different colour**.

[COMMENT #11] "Figure 4 could benefit from highlighting the bond newly formed during the macrocyclization."

[RESPONSE #11] We have **added the head-to-tail bond** in the figure as requested (now Figure 5).

Reviewer #3

[COMMENT #12] "The methods should contain at least brief details about peptide isolation from seeds. What mass of seeds was extracted? What is an estimate of the peptide yield? It is unclear why the reader is directed to a previous paper when other sections are more clearly described."

[RESPONSE #12] We have **added a brief description of the extraction method**. The **text is now expanded** as follows:

> Seed peptides were extracted as described by Jayasena et al. (2017). *Briefly, about 1 mL of seeds was ground with mortar and pestle and peptides extracted with a 4:4:1 mixture of methanol, dichloromethane and 0.05% (v/v) trifluoroacetic acid. Phases were separated by alternate addition of chloroform and 0.05% trifluoroacetic acid followed by centrifugation. The upper (aqueous) phase was dried in a vacuum centrifuge (Labconco), then the dried peptide extracts were dissolved in 50 – 100 µL of HPLC-grade water containing 5% (v/v) acetonitrile and 0.1% (v/v) formic acid (Honeywell).*

It is hard to say what the peptide yield was as we did not carry out quantitative mass spectrometry experiments because of the difficulty and expense of finding suitable calibration standards. The mass spectrometry signal strength varied enormously from seed to seed and peptide to peptide. As a comparison, we can say that the synthetic peptides were diluted to a concentration of approximately 5 ng/µL and gave a strong MS signal when 2 µL were injected for LC-MS. From this, we would say the yields of the native peptides were in the nanogram range or possibly below.

[COMMENT #13] "While the NMR structural data has been deposited to the PDB and BMRB, I still think there is value in seeing the NOESY and ROESY data in the Supporting information."

[RESPONSE #13] We **have added these spectra** to the manuscript. We put one example spectral pair as a figure (Figure 6) and put the rest in the Supporting Information (Supplemental Figures 53-56). We added appropriate captions and new text in the manuscript results section as follows:

> *The amino acid residues in synthetic PLP-12 were identified by a sequential walk of the H_α and H_N shifts of its ^1H-^1H TOCSY and ROESY spectra (Figure 6). The same procedure was followed for synthetic PLP-2, PLP-4 and PLP-10 (Supplemental Figures 53-56). Following this, ROESY peaks (NOESY in the case of PLP-2) were identified and used as restraints for structural modelling.*

Appendix/For reference: ORIGINAL FULL REPORT / REVIEWERS' COMMENTS:

Reviewer #1

The authors conducted a systematic analysis of the orbitides to elucidate protein family evolution scenario. The description and explanation of the result is thorough and to the point. The whole structure and organization of the paper is compactly organized. This is an excellent research paper on the whole.

Reviewer #2

The manuscript "A family of small, cyclic peptides buried in preproalbumin since the Eocene epoch" by Fisher et al. is about the isolation and characterization of new orbitides from the plant subfamily Asteroideae. These peptides were shown to belong to the natural product family of RiPPs and their precursor peptides are located at the N-terminal end of seed storage albumins and are released (and possibly cyclized) by the activities of asparaginyl endopeptidases. The compounds described in this work are exclusively PLPs, which are small, hydrophobic, cyclic peptides that, in contrast to PDPs, do not feature any disulfide bridges. By extensive transcriptome and peptide extract analysis the authors were able to identify 44 new peptides, whose primary structure was then confirmed by tandem MS analysis. From these, a subset of four peptides was synthesized and their 3D structures were solved by NMR spectroscopy. Additionally, analysis of the precursor proteins revealed total conservation of the C-terminal Asp residue in all observed peptides and conserved motifs flanking the core peptide on both ends.

Taken together, this is a well-written manuscript and a thoroughly performed study, where a plethora of new peptides from a young class of RiPPs that appear to be confined to Asteroideae were discovered and characterized. In my opinion, most of this work can be published as it is and I only have a few minor comments for the authors to address and consider.

Minor Comments:

- While the introduction is well-written and informative, I think it would benefit from an overview figure that summarizes the different classes of peptides the authors describe here, alongside their conserved regions and mode of processing.

- All the PLPs identified in this study end with an Asp residue and one starts with a Lys residue. While I believe that indeed the cyclization occurs exclusively between N- and C-terminus (especially because the authors did a careful comparison between some extracted PLPs with their synthetic analogs), I wonder if the authors have any other experimental evidence that it is not the side chain of the C-terminal Asp (or in one case the N-terminal Lys) that is involved in the macrocyclization. As the C-terminal Asp is apparently universal in these peptides, this might merit further discussion by the authors.

- The statement about PLPs not being antibacterial is one of the biggest issues I have with this study. The reason is that the authors only tested against a single E. coli strain, which does not justify such a general statement (with regard to the stated lack of antifungal activity, at least three strains were assessed). The authors should either revise that statement or expand their panel of tested organisms. If they want to do the latter, a variety of gram-positive and gram-negative bacteria should be used, of which some are ideally found in the same environment as the plant species from which the respective PLPs were discovered.

- The fact that some Asteroideae species produce more than one PLP might suggest that these are multicomponent antimicrobials that are only active in combination, but not on their own. This is something already observed in other RiPP classes, and while it cannot be addressed experimentally in this study, it could at least be discussed in the manuscript. Additionally, when discussing in what tribes the authors identified PLPs, they should also highlight which of these species produced more than one PLP.

- In the introduction, the authors mention recent works that traced the evolutionary history of STFI-1 and other bicyclic peptides to orbitide ancestors. As STFI-1 is a protease inhibitor (a feature also found for other cyclic peptides and other RiPPs like e.g. the microviridins), the authors should consider adding assays where they test their four synthetic PLPs for inhibition of commercially available proteases. Such assays could be performed very quickly and, even if they fail, would be interesting for comparison to STFI-1.

- At the beginning of their discussion section, the authors mention that 22 of the undetected peptide sequences had no C-terminal Asp and thus were not being processed by the respective organisms. I would welcome if they could also discuss, if these peptides contain the conserved flanking regions described in Figure 4 or lack them as well. Furthermore, the authors should comment if there is any evidence of the according preproteins being either evolutionary ancestors from which the PLP-containing preproteins were derived or if they look more like they were PLP-containing proteins at one point, but then got inactivated by random mutation of the C-terminal Asp residue.

- In the first paragraph of the "LC-MS/MS and Peptide Sequencing" section in the Materials & Methods the authors should mention which column they used.

- The MS2 spectra containing figures are pretty packed with information to a point where they become a bit confusing. While it would be too much effort to change all of these, the authors could at least add some color to Figure 2 and 3 in the main text, to make them more accessible to the reader.

- Figure 4 could benefit from highlighting the bond newly formed during the macrocylization.

Reviewer #3

This study focuses on the isolation of orbitides, short head-to-tail cyclized peptides, from plant seeds. Sequencing of the transcriptome of 110 species led to the discovery of putative orbitides in 50 species. Mass spectrometry confirmed the presence of 46 different peptides in 17 species. The authors also present the NMR structures 4 of the peptides, one of which exists in two different conformers. One of the peptides was not soluble in water, so the structure was solved instead in DMSO. There have been previous reports of orbitides with antimicrobial/antifungal activity, but none of the peptides tested here exhibited these activities. Overall this is an impressive body of work that greatly expands the list of experimentally confirmed orbitides. I would be in support of publication if several minor points can be addressed.

1) The methods should contain at least brief details about peptide isolation from seeds. What mass of seeds was extracted? What is an estimate of the peptide yield? It is unclear why the reader is directed to a previous paper when other sections are more clearly described.

2) While the NMR structural data has been deposited to the PDB and BMRB, I still think there is value in seeing the NOESY and ROESY data in the Supporting information.
